# Supplementary figures and images for: Comparative Transcriptome and sRNAome Analyses Reveal the Regulatory Mechanisms of Fruit Ripening in a Spontaneous Early-Ripening Navel Orange Mutant and Its Wild Type (part 2 of 2)
Source: Genes (Basel). 2022 Sep 22;13(10):1706. doi: 10.3390/genes13101706 (PMC9601947; doi:10.3390/genes13101706)

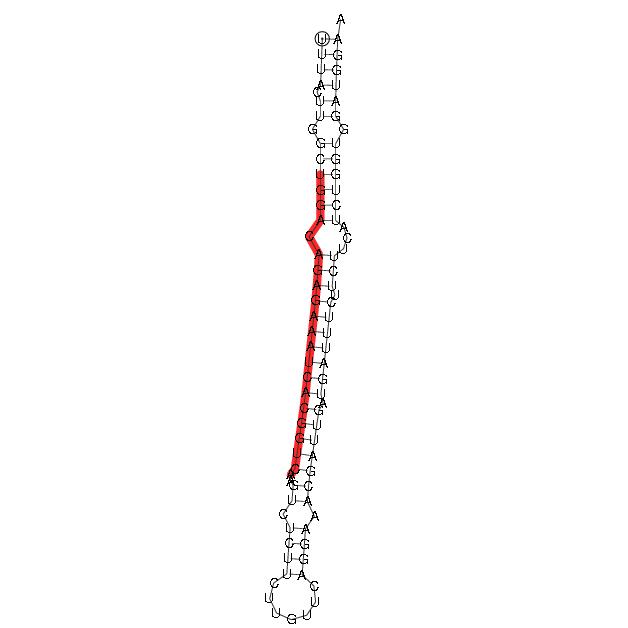

Supplement: Supplementary file 1 [file genes-13-01706-s001.zip › Figure S1. Known miRNAs Structure/csi-miR3954_csi-MIR3954.jpg]

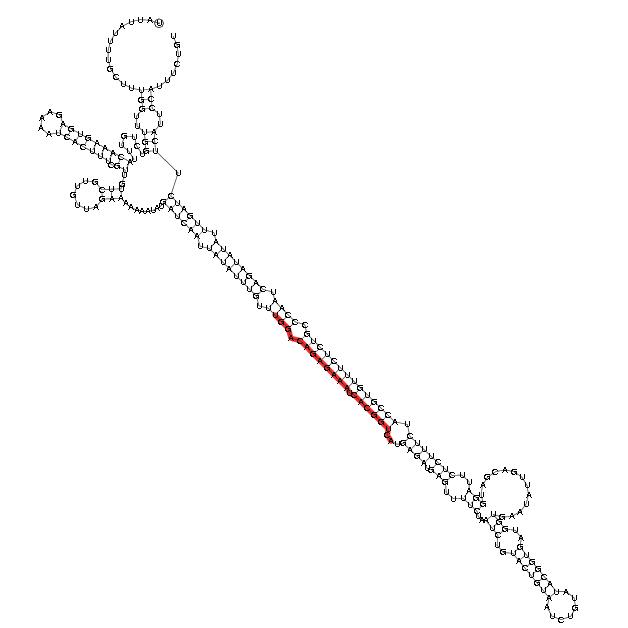

Supplement: Supplementary file 1 [file genes-13-01706-s001.zip › Figure S1. Known miRNAs Structure/csi-miR3954_csi-MIR3954b.jpg]

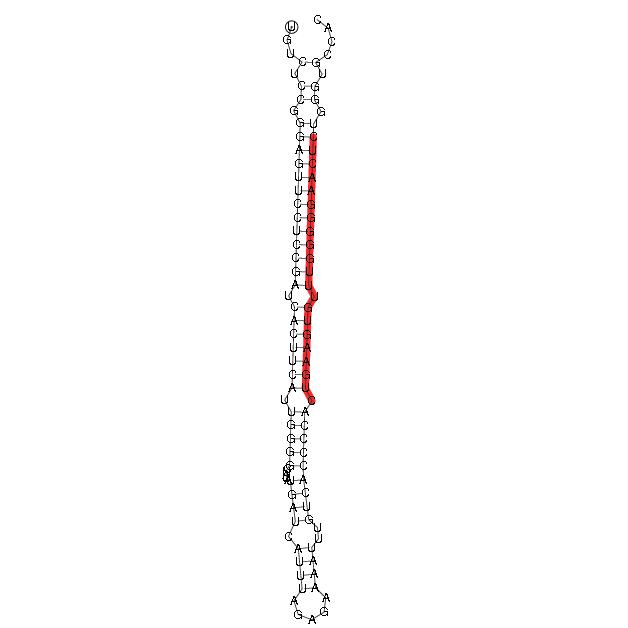

Supplement: Supplementary file 1 [file genes-13-01706-s001.zip › Figure S1. Known miRNAs Structure/csi-miR395a_csi-MIR395a.jpg]

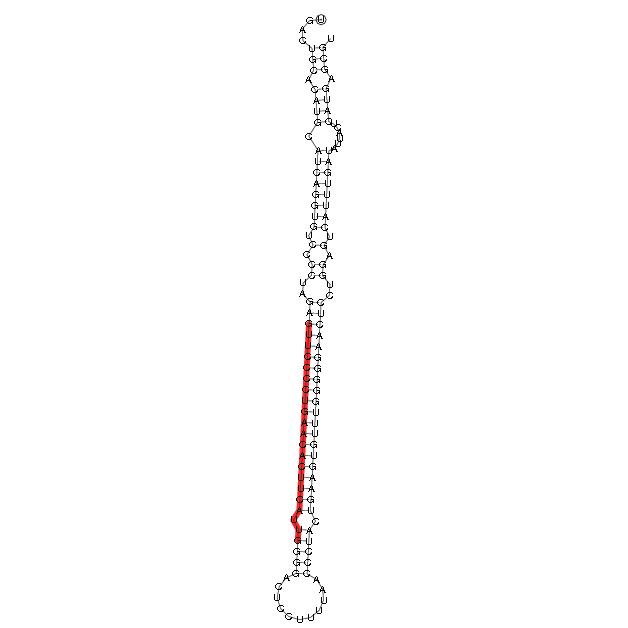

Supplement: Supplementary file 1 [file genes-13-01706-s001.zip › Figure S1. Known miRNAs Structure/csi-miR395b-5p_csi-MIR395b.jpg]

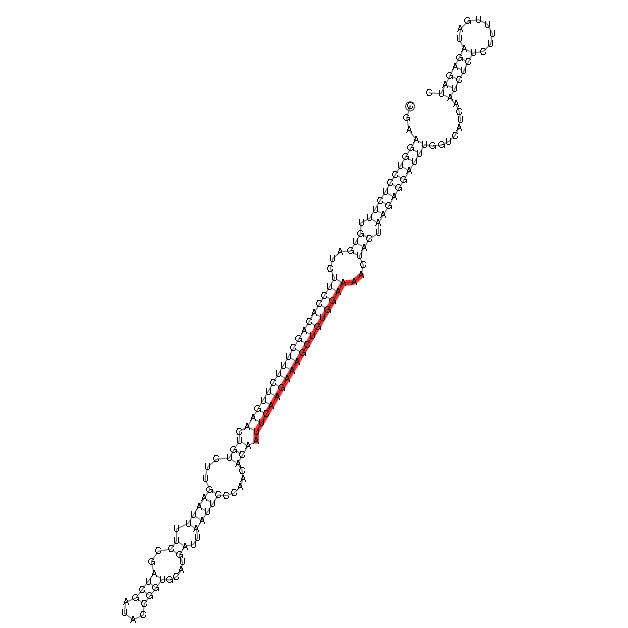

Supplement: Supplementary file 1 [file genes-13-01706-s001.zip › Figure S1. Known miRNAs Structure/csi-miR396a-3p_csi-MIR396a.jpg]

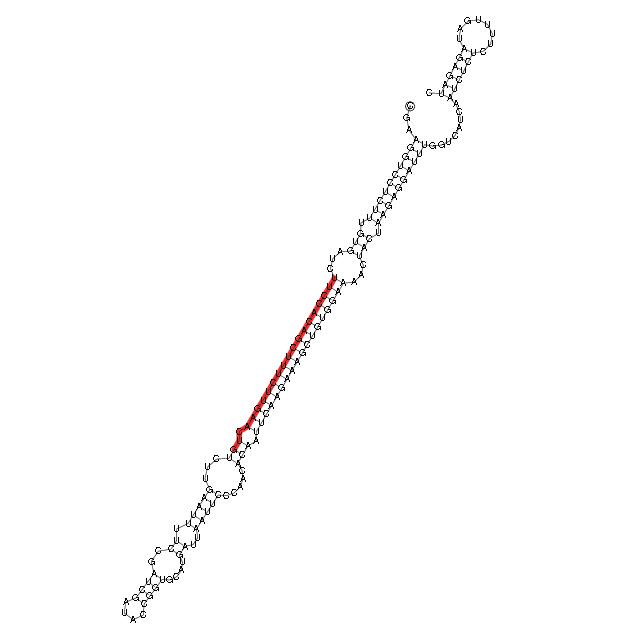

Supplement: Supplementary file 1 [file genes-13-01706-s001.zip › Figure S1. Known miRNAs Structure/csi-miR396a-5p_csi-MIR396a.jpg]

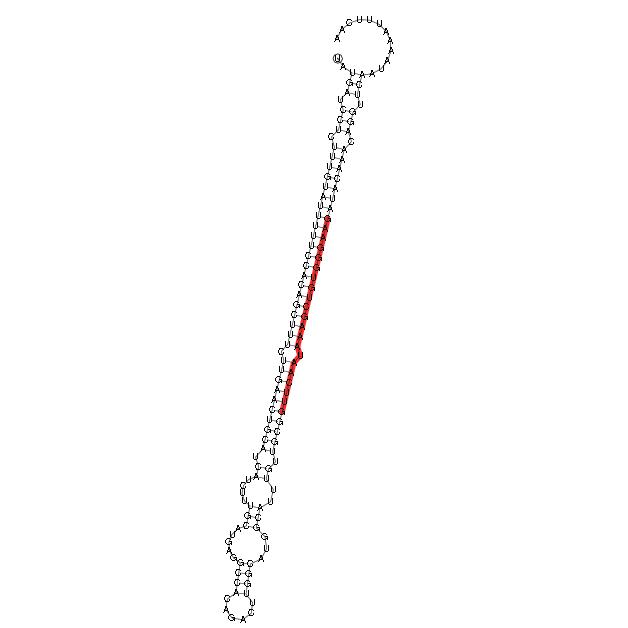

Supplement: Supplementary file 1 [file genes-13-01706-s001.zip › Figure S1. Known miRNAs Structure/csi-miR396b-3p_csi-MIR396b.jpg]

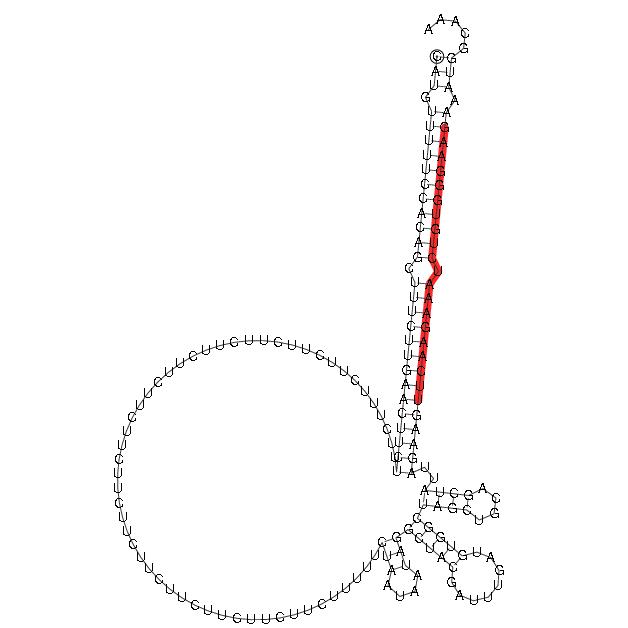

Supplement: Supplementary file 1 [file genes-13-01706-s001.zip › Figure S1. Known miRNAs Structure/csi-miR396c_csi-MIR396c.jpg]

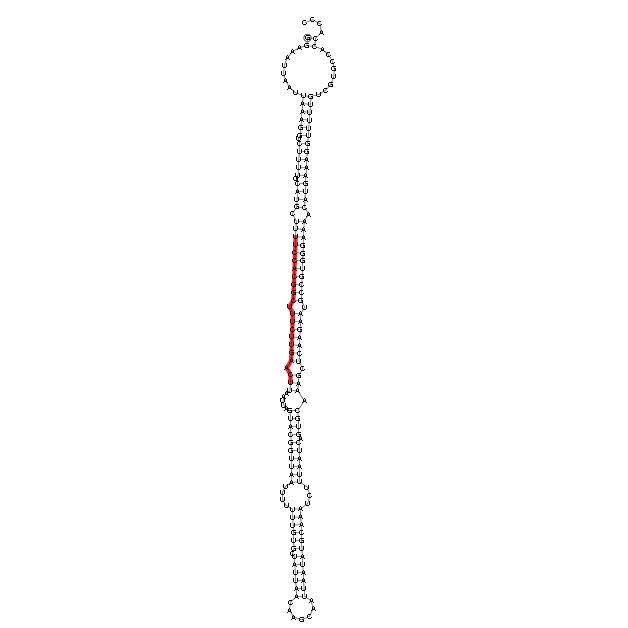

Supplement: Supplementary file 1 [file genes-13-01706-s001.zip › Figure S1. Known miRNAs Structure/csi-miR396e-5p_csi-MIR396e.jpg]

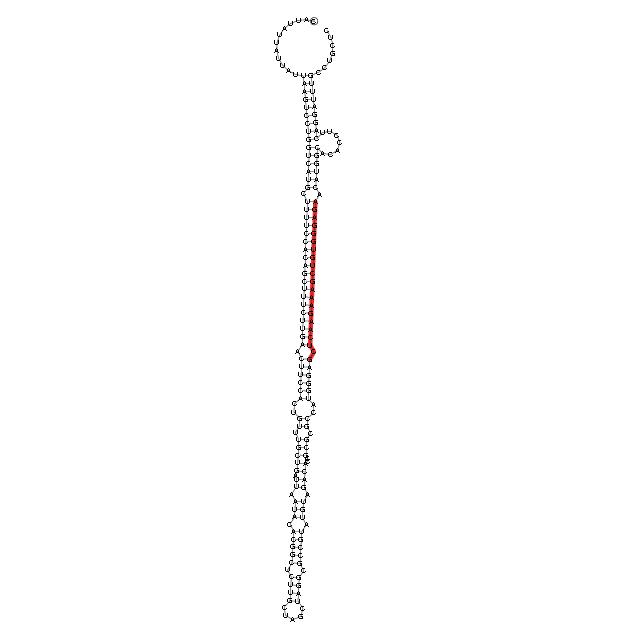

Supplement: Supplementary file 1 [file genes-13-01706-s001.zip › Figure S1. Known miRNAs Structure/csi-miR396f-3p_csi-MIR396f.jpg]

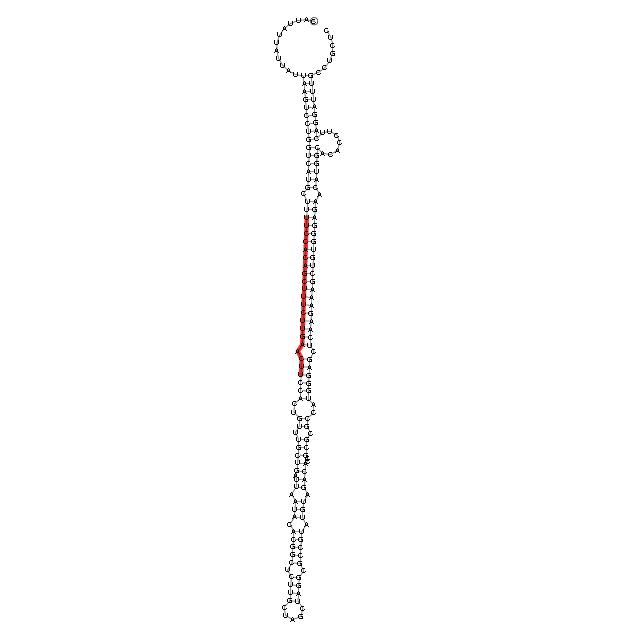

Supplement: Supplementary file 1 [file genes-13-01706-s001.zip › Figure S1. Known miRNAs Structure/csi-miR396f-5p_csi-MIR396f.jpg]

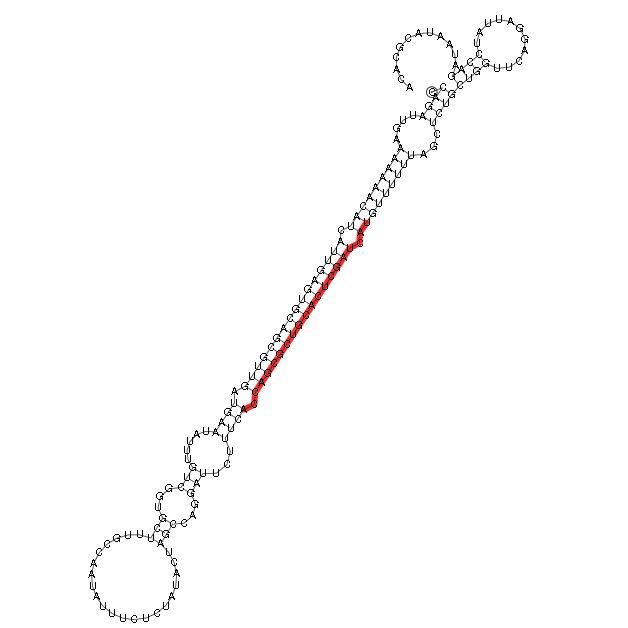

Supplement: Supplementary file 1 [file genes-13-01706-s001.zip › Figure S1. Known miRNAs Structure/csi-miR397-3p_csi-MIR397.jpg]

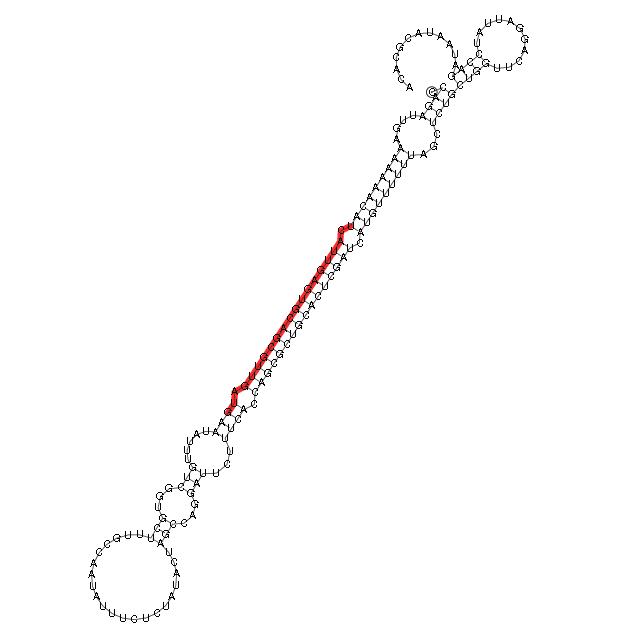

Supplement: Supplementary file 1 [file genes-13-01706-s001.zip › Figure S1. Known miRNAs Structure/csi-miR397-5p_csi-MIR397.jpg]

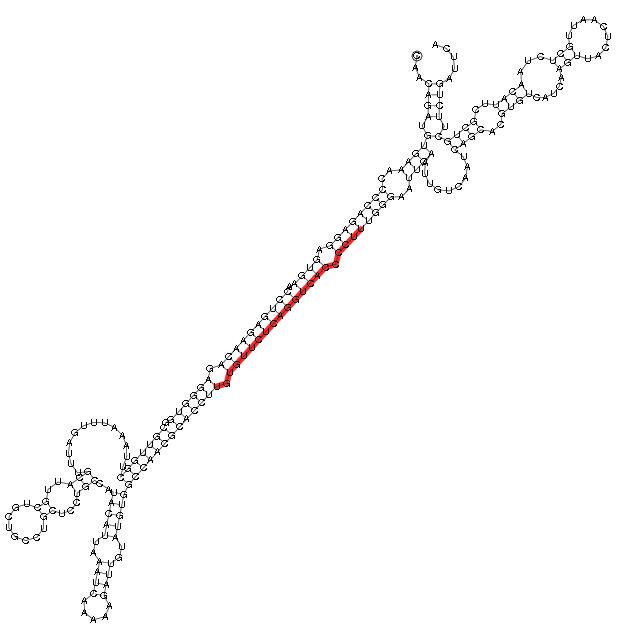

Supplement: Supplementary file 1 [file genes-13-01706-s001.zip › Figure S1. Known miRNAs Structure/csi-miR398a-3p_csi-MIR398a.jpg]

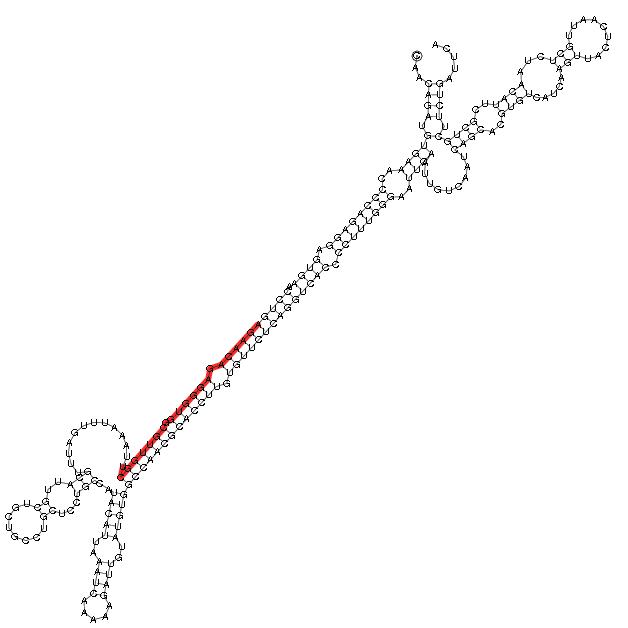

Supplement: Supplementary file 1 [file genes-13-01706-s001.zip › Figure S1. Known miRNAs Structure/csi-miR398a-5p_csi-MIR398a.jpg]

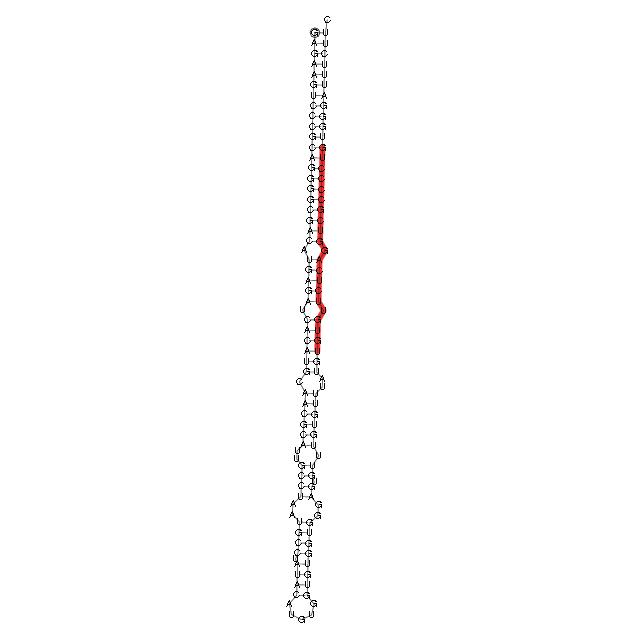

Supplement: Supplementary file 1 [file genes-13-01706-s001.zip › Figure S1. Known miRNAs Structure/csi-miR398b-3p_csi-MIR398b.jpg]

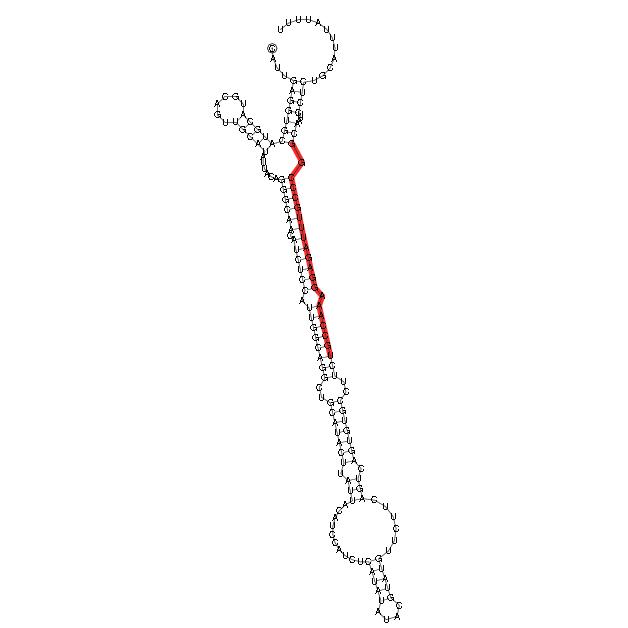

Supplement: Supplementary file 1 [file genes-13-01706-s001.zip › Figure S1. Known miRNAs Structure/csi-miR399a-3p_csi-MIR399a.jpg]

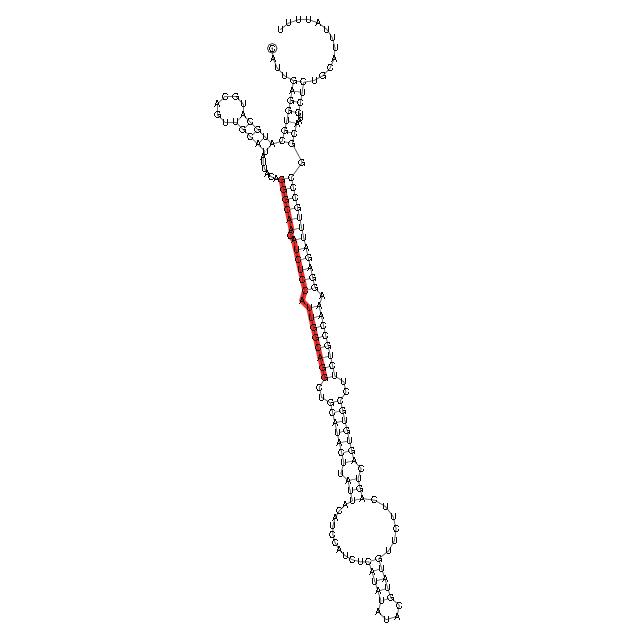

Supplement: Supplementary file 1 [file genes-13-01706-s001.zip › Figure S1. Known miRNAs Structure/csi-miR399a-5p_csi-MIR399a.jpg]

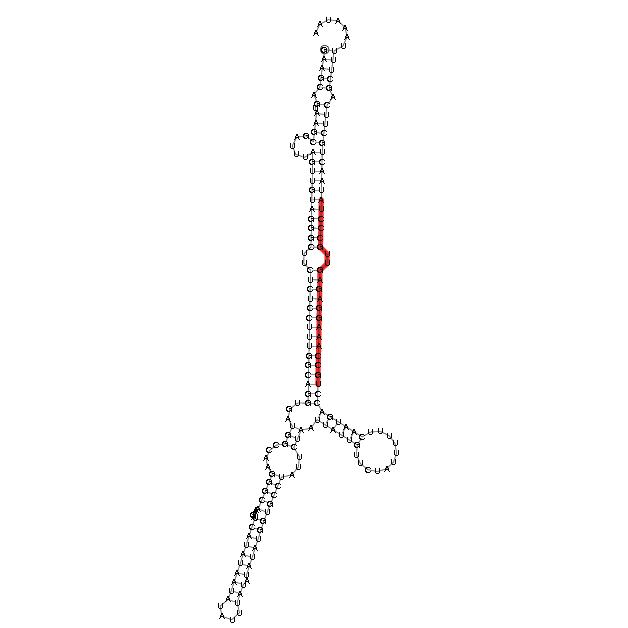

Supplement: Supplementary file 1 [file genes-13-01706-s001.zip › Figure S1. Known miRNAs Structure/csi-miR399b-3p_csi-MIR399b.jpg]

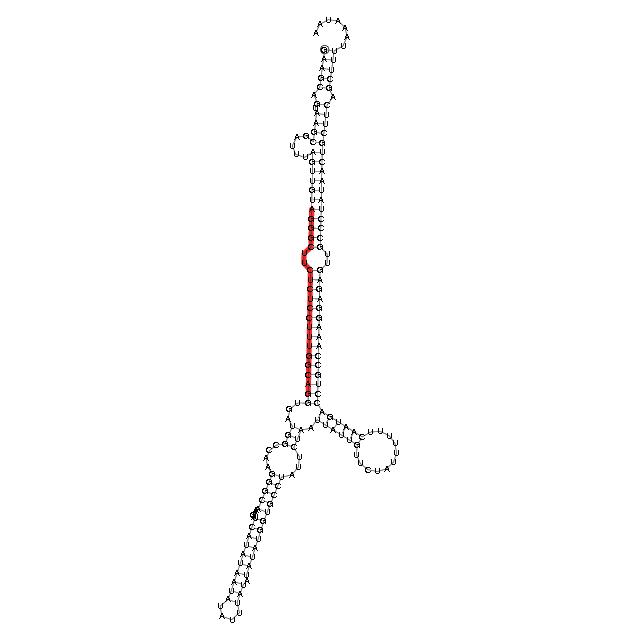

Supplement: Supplementary file 1 [file genes-13-01706-s001.zip › Figure S1. Known miRNAs Structure/csi-miR399b-5p_csi-MIR399b.jpg]

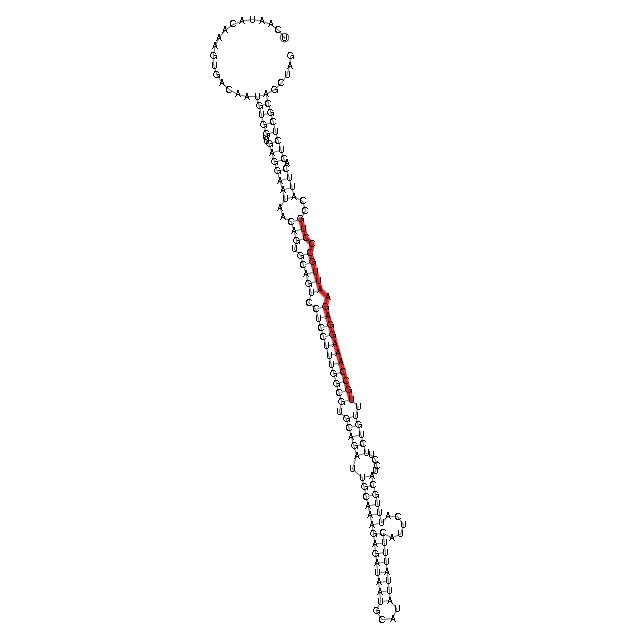

Supplement: Supplementary file 1 [file genes-13-01706-s001.zip › Figure S1. Known miRNAs Structure/csi-miR399c-3p_csi-MIR399c.jpg]

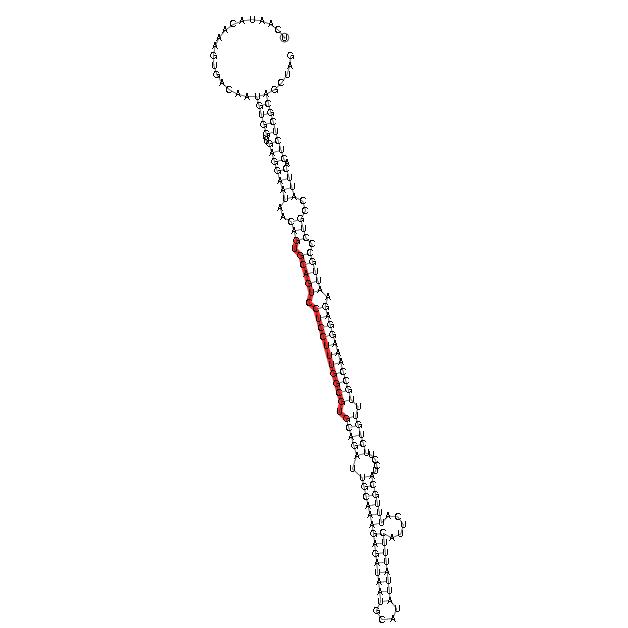

Supplement: Supplementary file 1 [file genes-13-01706-s001.zip › Figure S1. Known miRNAs Structure/csi-miR399c-5p_csi-MIR399c.jpg]

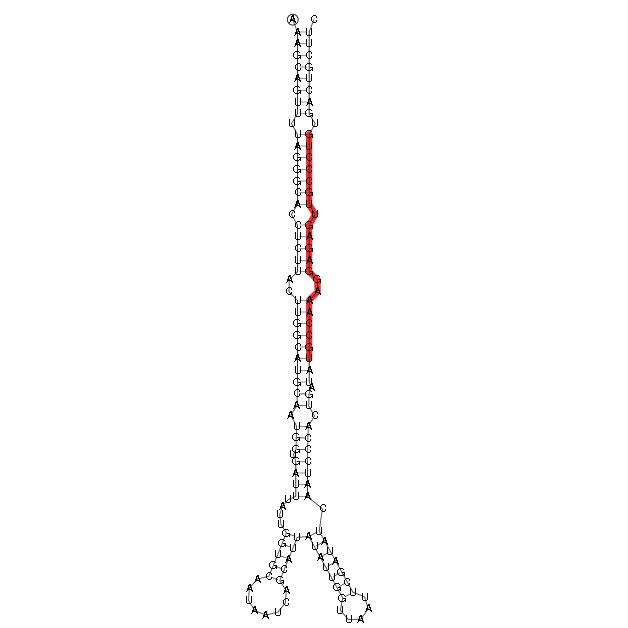

Supplement: Supplementary file 1 [file genes-13-01706-s001.zip › Figure S1. Known miRNAs Structure/csi-miR399d-3p_csi-MIR399d.jpg]

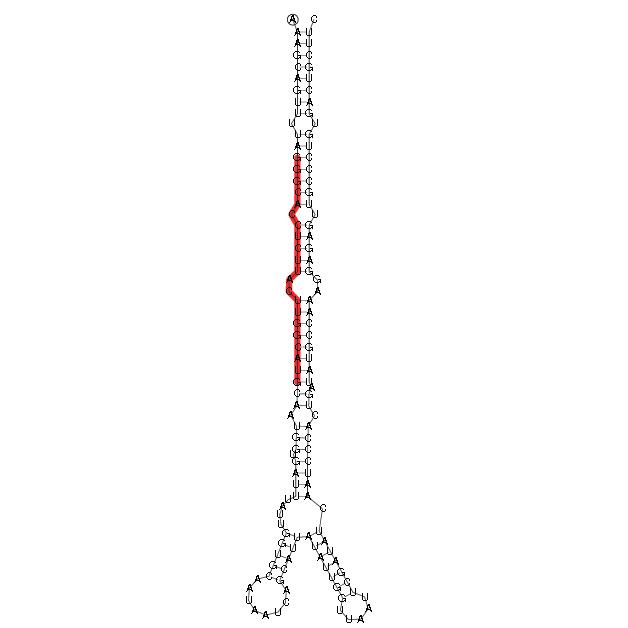

Supplement: Supplementary file 1 [file genes-13-01706-s001.zip › Figure S1. Known miRNAs Structure/csi-miR399d-5p_csi-MIR399d.jpg]

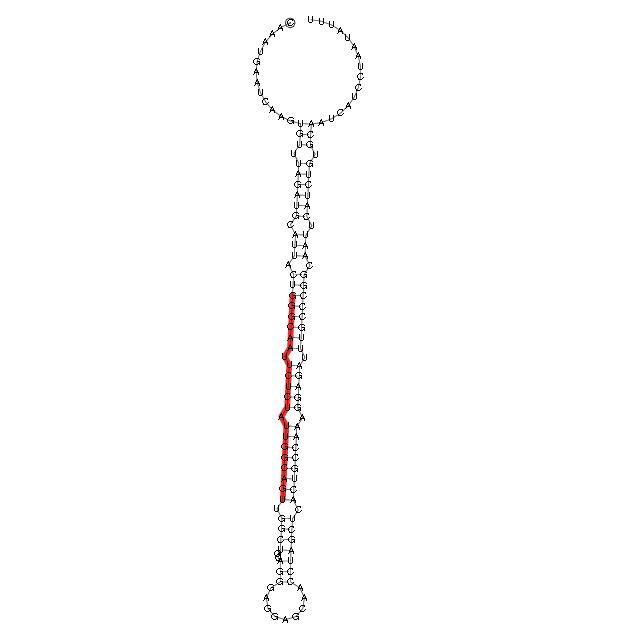

Supplement: Supplementary file 1 [file genes-13-01706-s001.zip › Figure S1. Known miRNAs Structure/csi-miR399e-5p_csi-MIR399e.jpg]

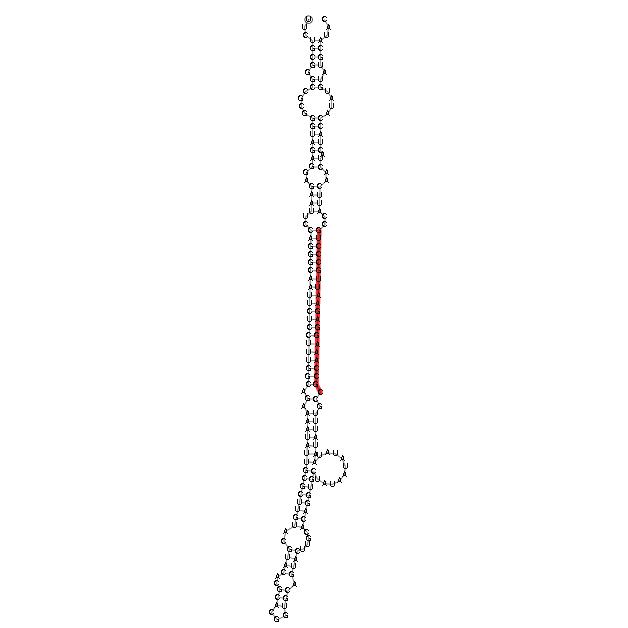

Supplement: Supplementary file 1 [file genes-13-01706-s001.zip › Figure S1. Known miRNAs Structure/csi-miR399f-3p_csi-MIR399f.jpg]

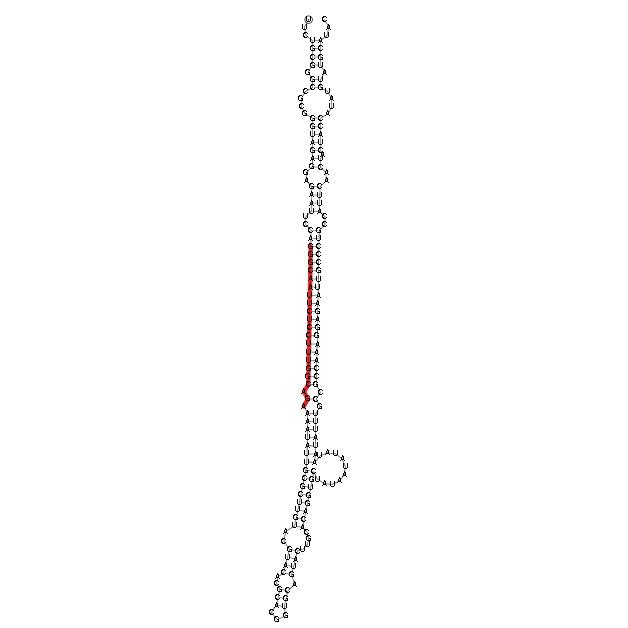

Supplement: Supplementary file 1 [file genes-13-01706-s001.zip › Figure S1. Known miRNAs Structure/csi-miR399f-5p_csi-MIR399f.jpg]

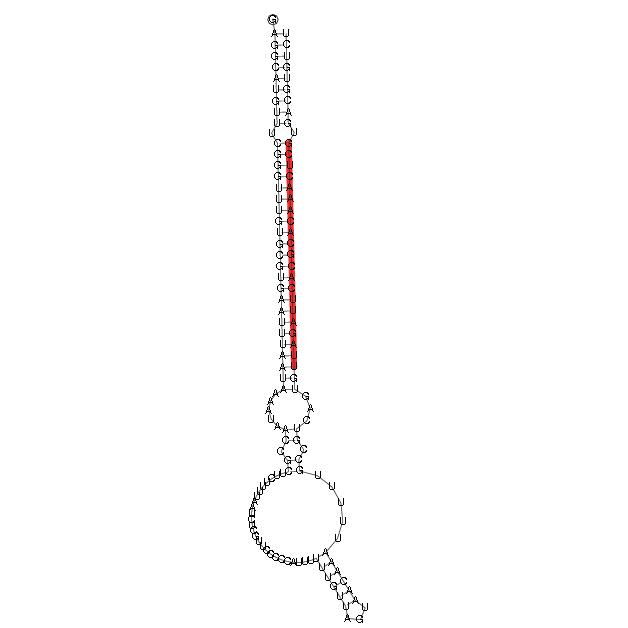

Supplement: Supplementary file 1 [file genes-13-01706-s001.zip › Figure S1. Known miRNAs Structure/csi-miR403a-3p_csi-MIR403a.jpg]

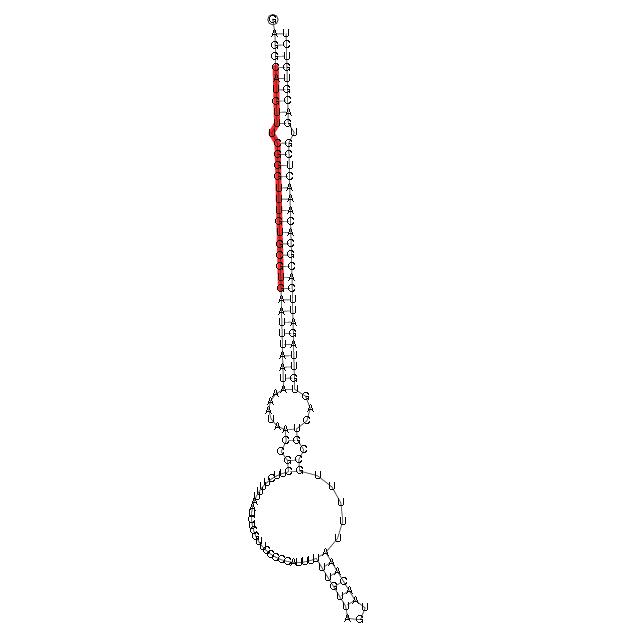

Supplement: Supplementary file 1 [file genes-13-01706-s001.zip › Figure S1. Known miRNAs Structure/csi-miR403a-5p_csi-MIR403a.jpg]

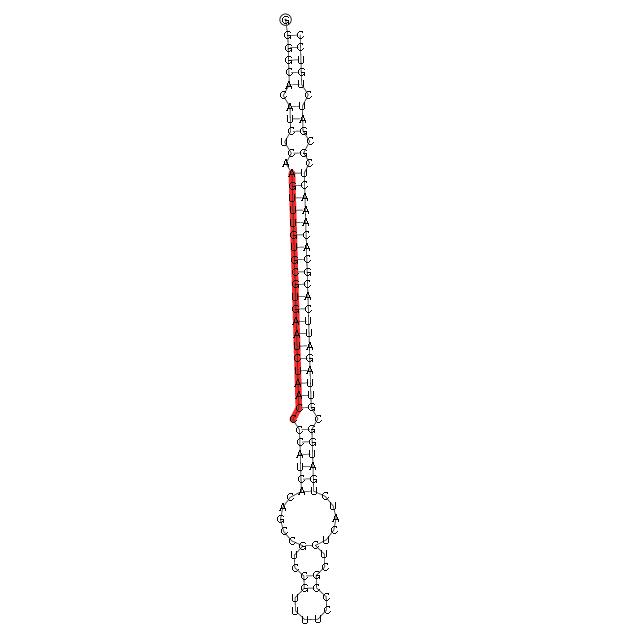

Supplement: Supplementary file 1 [file genes-13-01706-s001.zip › Figure S1. Known miRNAs Structure/csi-miR403b-5p_csi-MIR403b.jpg]

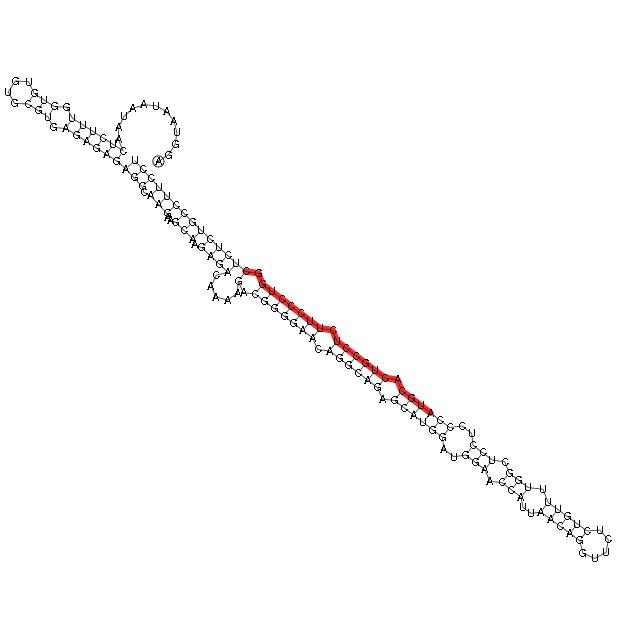

Supplement: Supplementary file 1 [file genes-13-01706-s001.zip › Figure S1. Known miRNAs Structure/csi-miR408-3p_csi-MIR408.jpg]

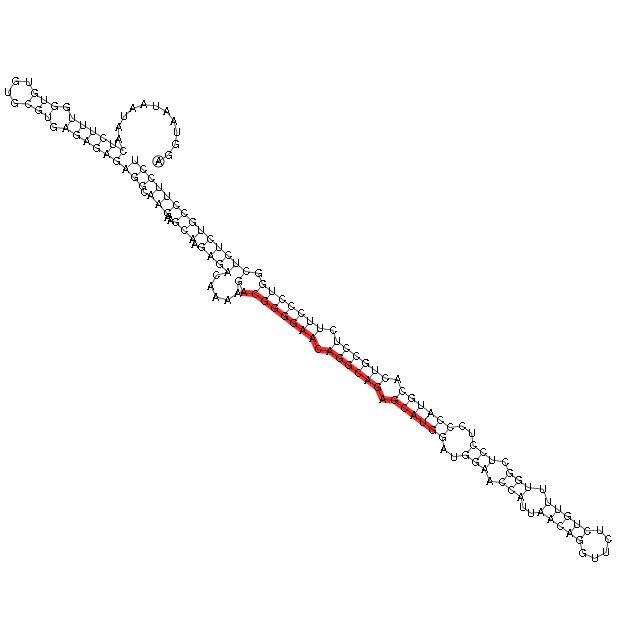

Supplement: Supplementary file 1 [file genes-13-01706-s001.zip › Figure S1. Known miRNAs Structure/csi-miR408-5p_csi-MIR408.jpg]

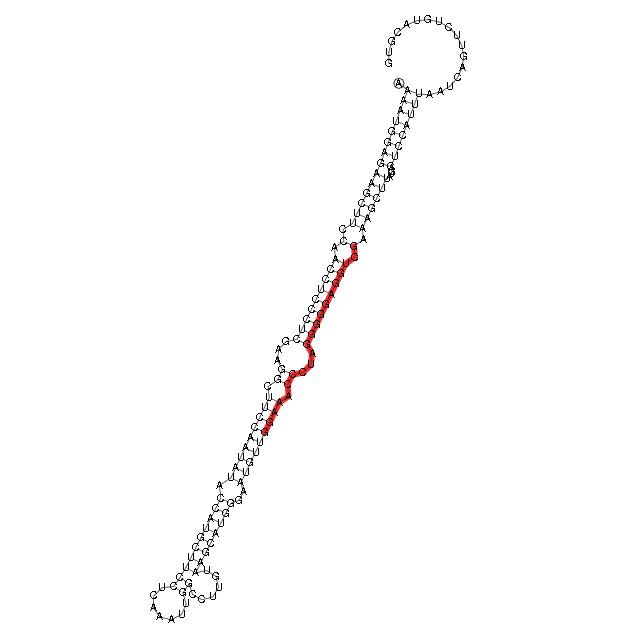

Supplement: Supplementary file 1 [file genes-13-01706-s001.zip › Figure S1. Known miRNAs Structure/csi-miR477a-3p_csi-MIR477a.jpg]

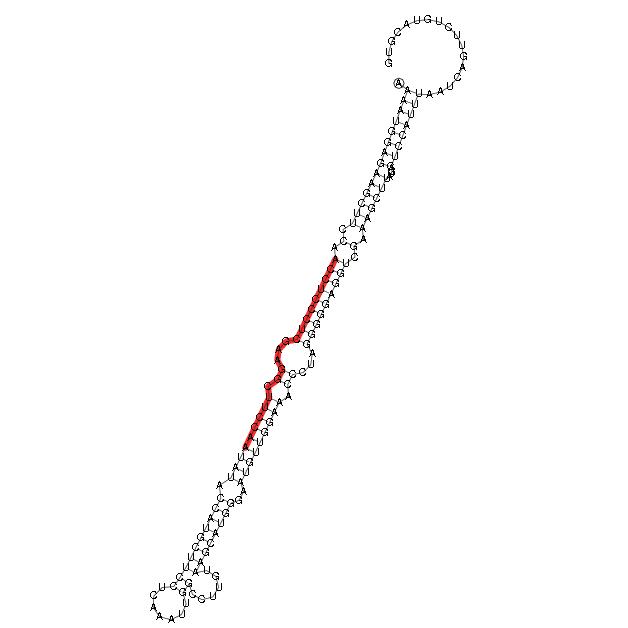

Supplement: Supplementary file 1 [file genes-13-01706-s001.zip › Figure S1. Known miRNAs Structure/csi-miR477a-5p_csi-MIR477a.jpg]

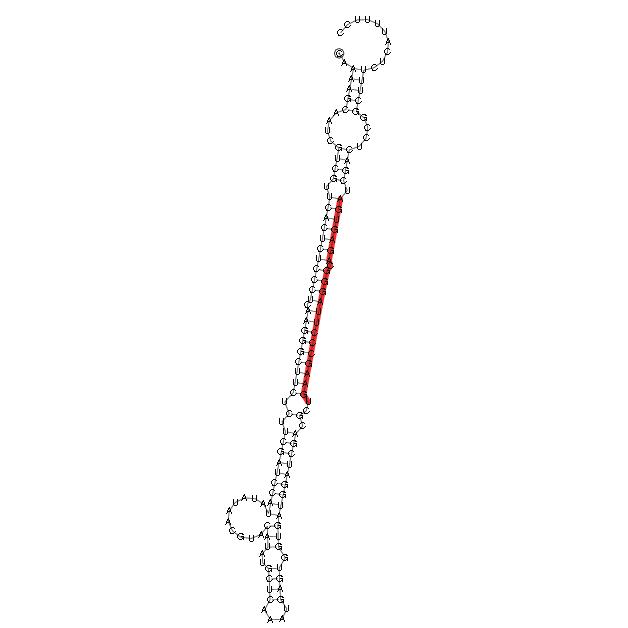

Supplement: Supplementary file 1 [file genes-13-01706-s001.zip › Figure S1. Known miRNAs Structure/csi-miR477b-3p_csi-MIR477b.jpg]

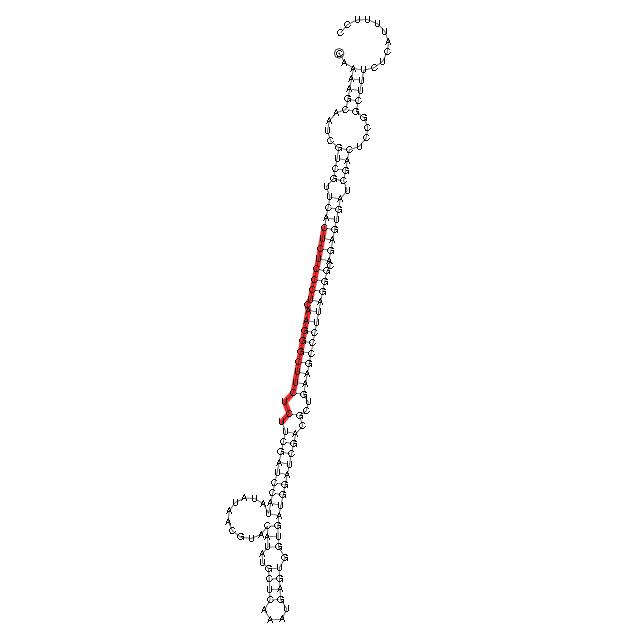

Supplement: Supplementary file 1 [file genes-13-01706-s001.zip › Figure S1. Known miRNAs Structure/csi-miR477b-5p_csi-MIR477b.jpg]

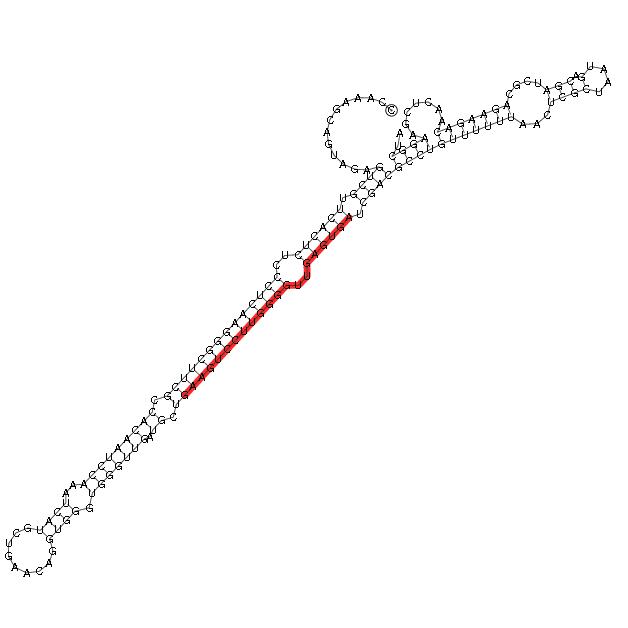

Supplement: Supplementary file 1 [file genes-13-01706-s001.zip › Figure S1. Known miRNAs Structure/csi-miR477c-3p_csi-MIR477c.jpg]

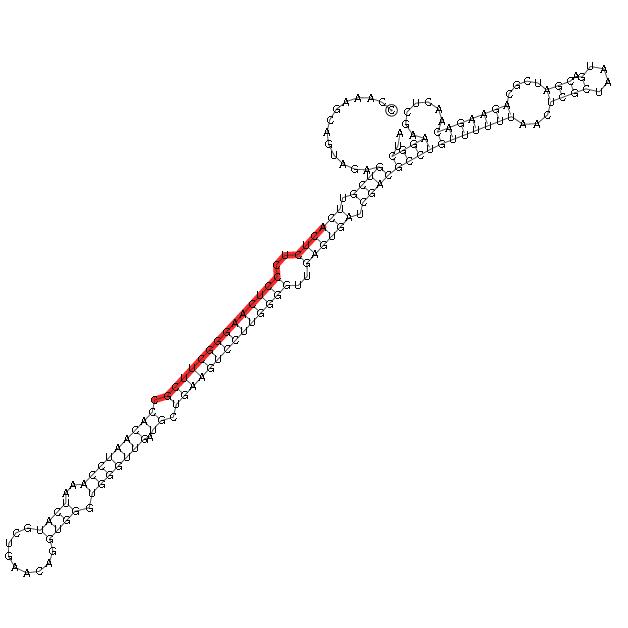

Supplement: Supplementary file 1 [file genes-13-01706-s001.zip › Figure S1. Known miRNAs Structure/csi-miR477c-5p_csi-MIR477c.jpg]

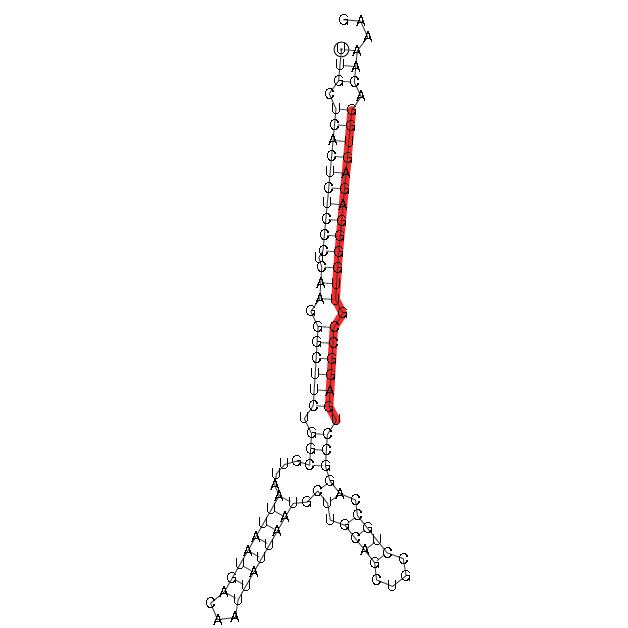

Supplement: Supplementary file 1 [file genes-13-01706-s001.zip › Figure S1. Known miRNAs Structure/csi-miR477d-3p_csi-MIR477d.jpg]

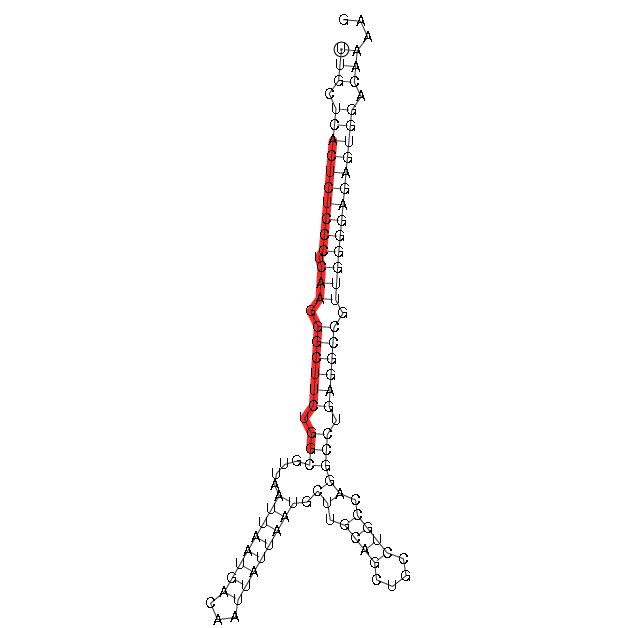

Supplement: Supplementary file 1 [file genes-13-01706-s001.zip › Figure S1. Known miRNAs Structure/csi-miR477d-5p_csi-MIR477d.jpg]

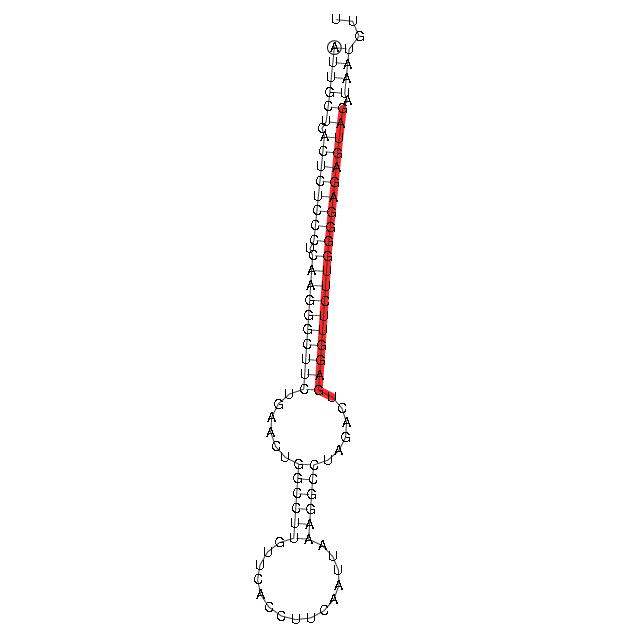

Supplement: Supplementary file 1 [file genes-13-01706-s001.zip › Figure S1. Known miRNAs Structure/csi-miR477e-3p_csi-MIR477e.jpg]

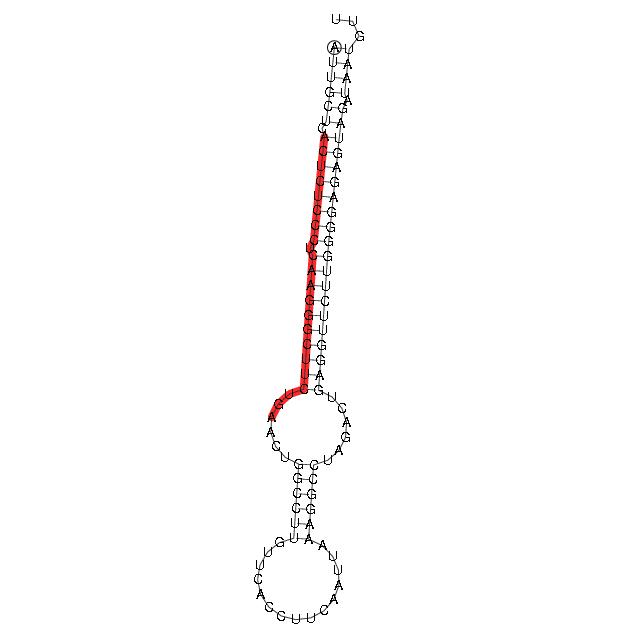

Supplement: Supplementary file 1 [file genes-13-01706-s001.zip › Figure S1. Known miRNAs Structure/csi-miR477e-5p_csi-MIR477e.jpg]

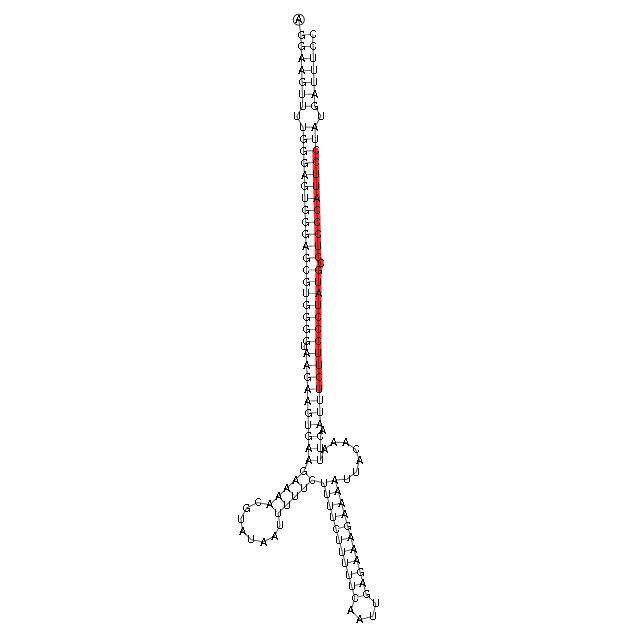

Supplement: Supplementary file 1 [file genes-13-01706-s001.zip › Figure S1. Known miRNAs Structure/csi-miR482a-3p_csi-MIR482a.jpg]

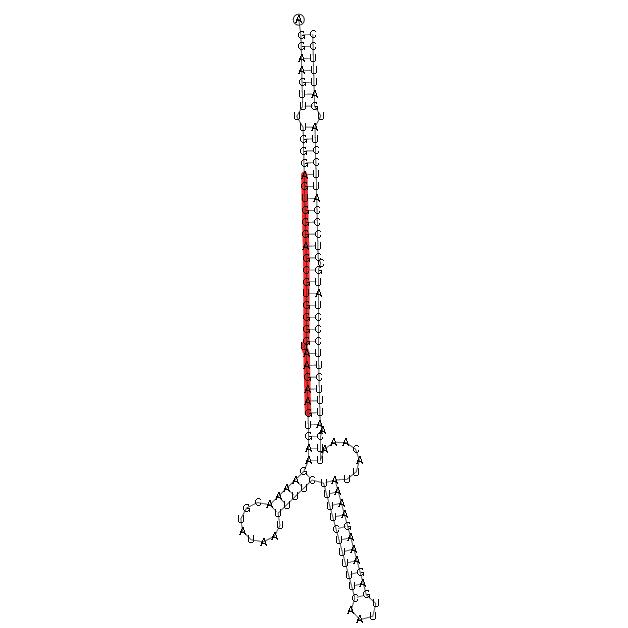

Supplement: Supplementary file 1 [file genes-13-01706-s001.zip › Figure S1. Known miRNAs Structure/csi-miR482a-5p_csi-MIR482a.jpg]

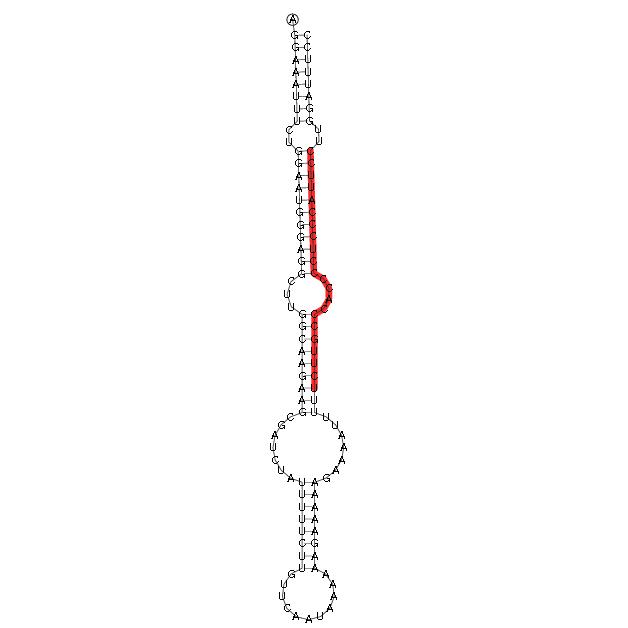

Supplement: Supplementary file 1 [file genes-13-01706-s001.zip › Figure S1. Known miRNAs Structure/csi-miR482b-3p_csi-MIR482b.jpg]

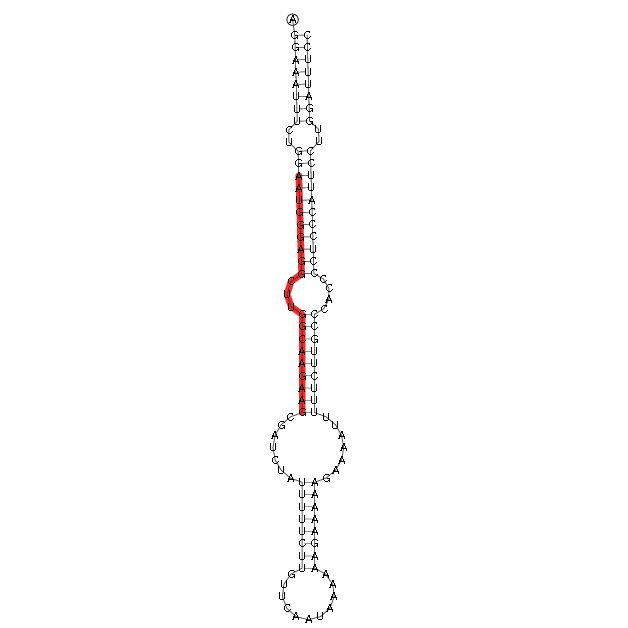

Supplement: Supplementary file 1 [file genes-13-01706-s001.zip › Figure S1. Known miRNAs Structure/csi-miR482b-5p_csi-MIR482b.jpg]

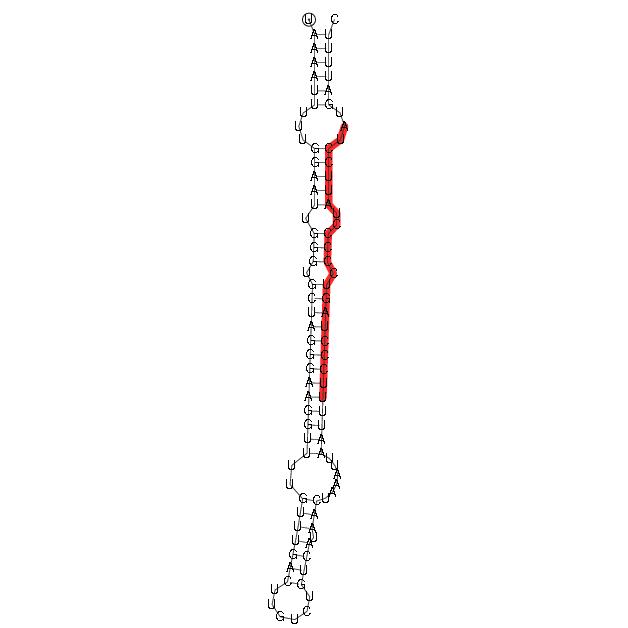

Supplement: Supplementary file 1 [file genes-13-01706-s001.zip › Figure S1. Known miRNAs Structure/csi-miR482c-3p_csi-MIR482c.jpg]

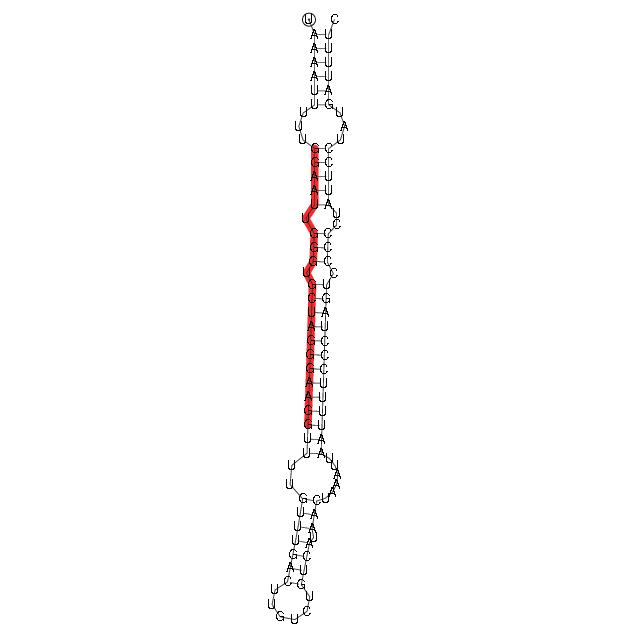

Supplement: Supplementary file 1 [file genes-13-01706-s001.zip › Figure S1. Known miRNAs Structure/csi-miR482c-5p_csi-MIR482c.jpg]

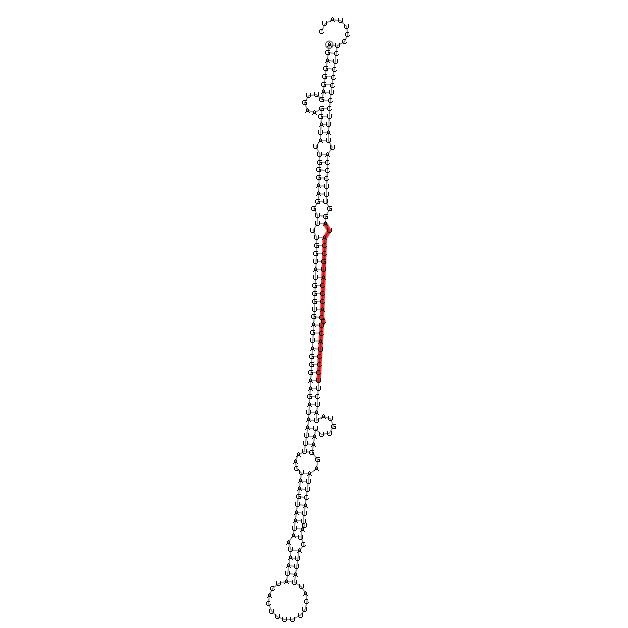

Supplement: Supplementary file 1 [file genes-13-01706-s001.zip › Figure S1. Known miRNAs Structure/csi-miR482d-3p_csi-MIR482d.jpg]

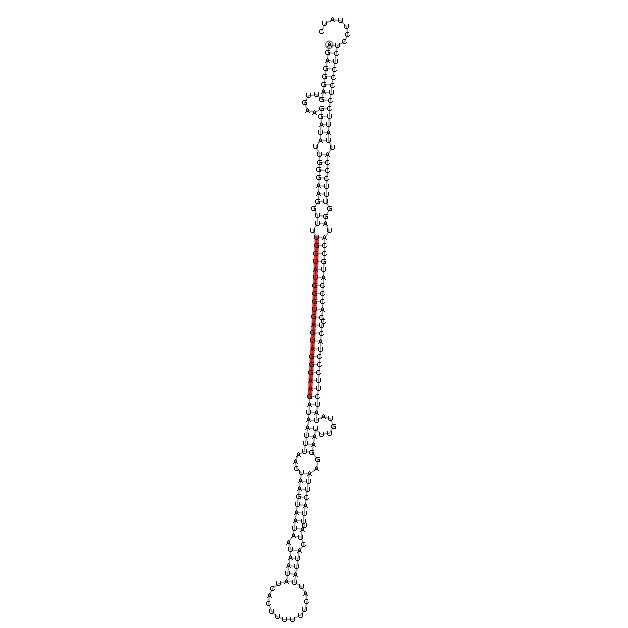

Supplement: Supplementary file 1 [file genes-13-01706-s001.zip › Figure S1. Known miRNAs Structure/csi-miR482d-5p_csi-MIR482d.jpg]

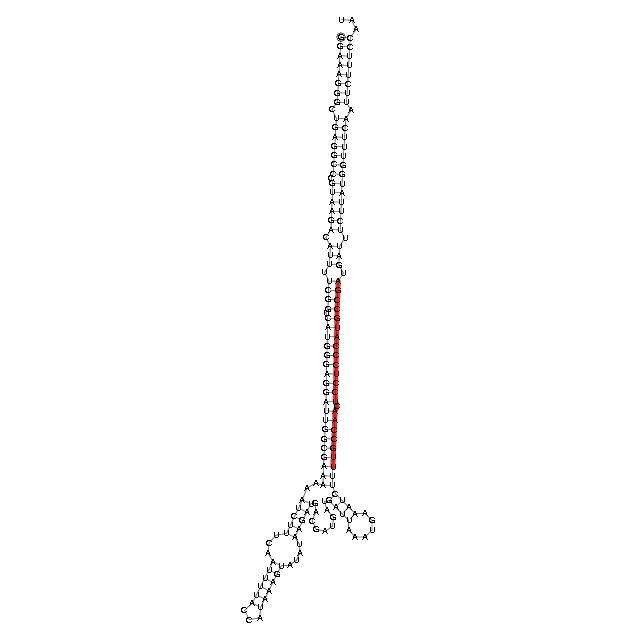

Supplement: Supplementary file 1 [file genes-13-01706-s001.zip › Figure S1. Known miRNAs Structure/csi-miR482e-3p_csi-MIR482e.jpg]

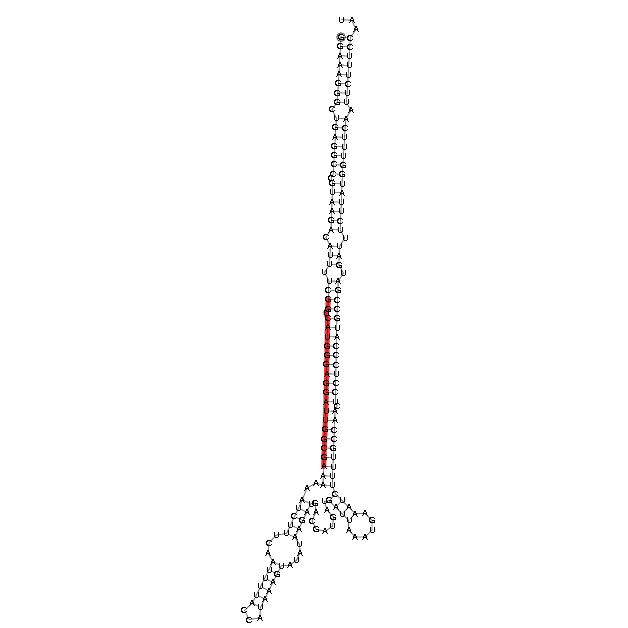

Supplement: Supplementary file 1 [file genes-13-01706-s001.zip › Figure S1. Known miRNAs Structure/csi-miR482e-5p_csi-MIR482e.jpg]

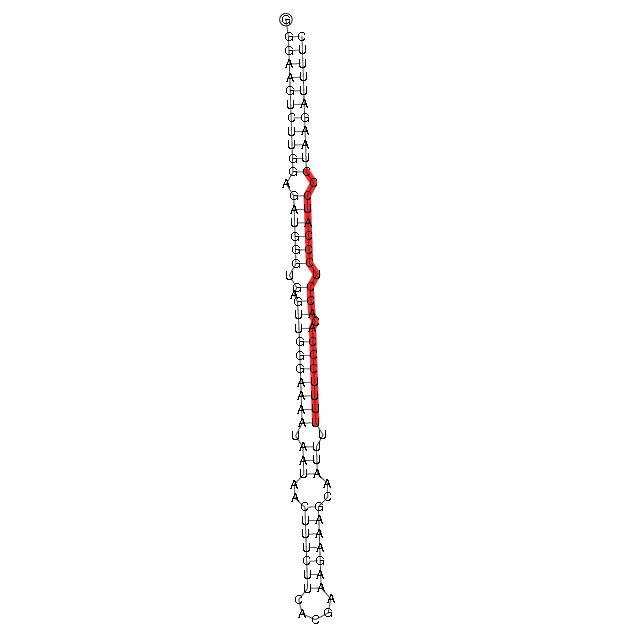

Supplement: Supplementary file 1 [file genes-13-01706-s001.zip › Figure S1. Known miRNAs Structure/csi-miR482f-3p_csi-MIR482f.jpg]

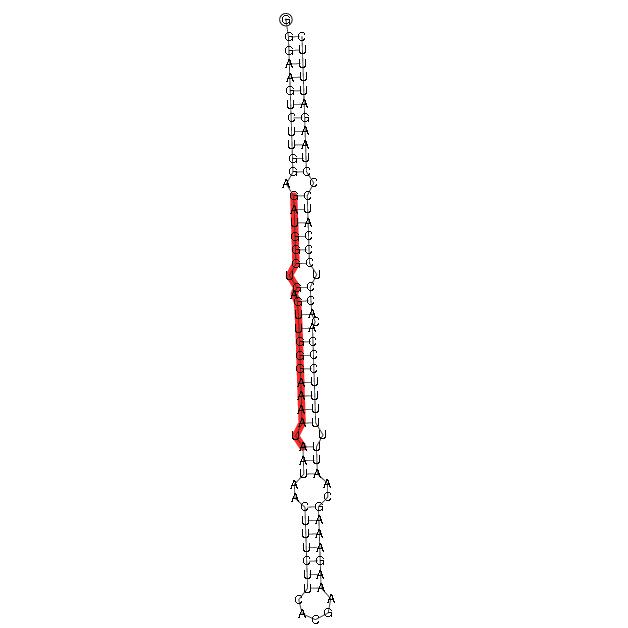

Supplement: Supplementary file 1 [file genes-13-01706-s001.zip › Figure S1. Known miRNAs Structure/csi-miR482f-5p_csi-MIR482f.jpg]

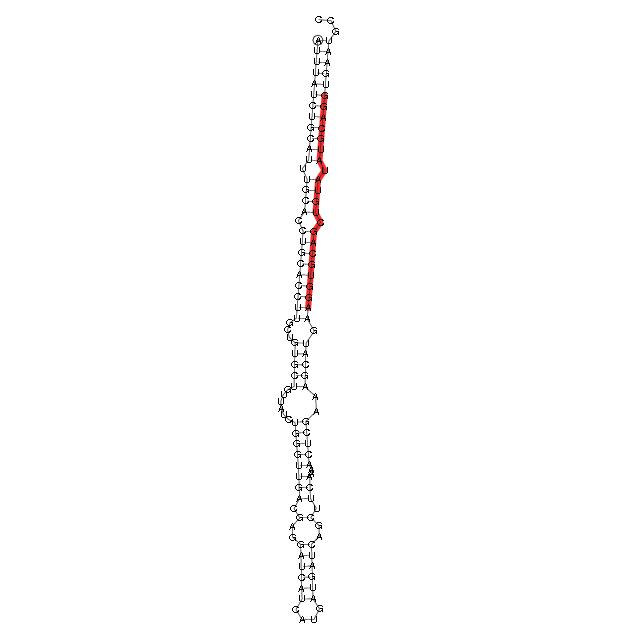

Supplement: Supplementary file 1 [file genes-13-01706-s001.zip › Figure S1. Known miRNAs Structure/csi-miR530a-3p_csi-MIR530a.jpg]

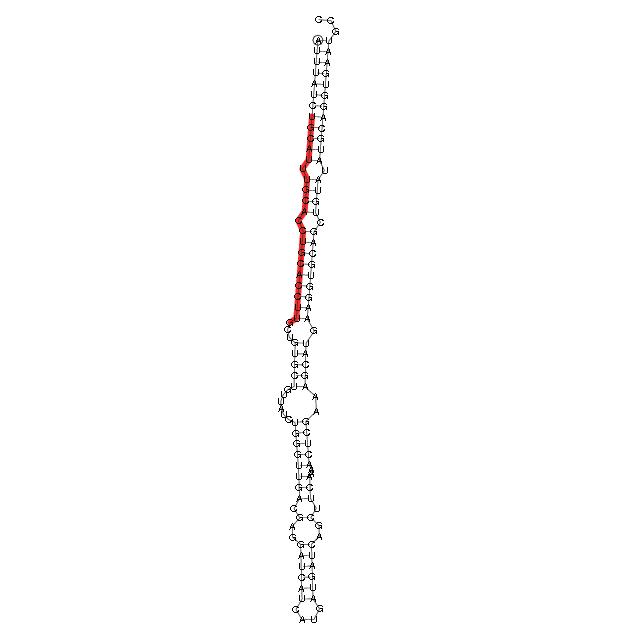

Supplement: Supplementary file 1 [file genes-13-01706-s001.zip › Figure S1. Known miRNAs Structure/csi-miR530a-5p_csi-MIR530a.jpg]

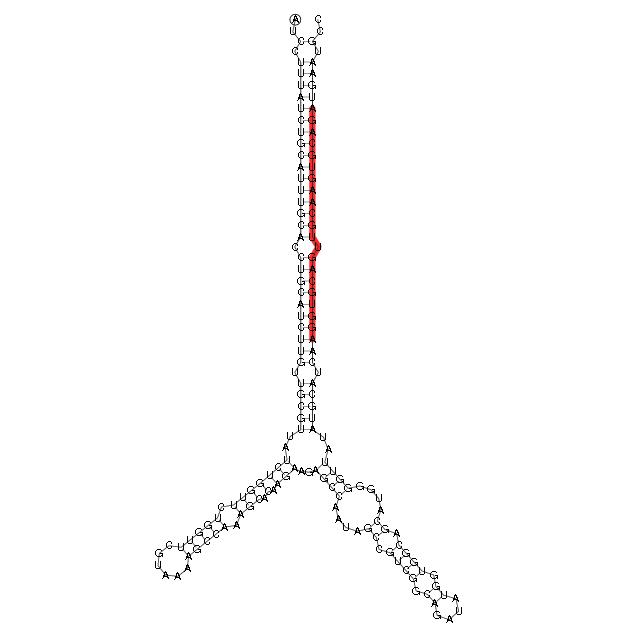

Supplement: Supplementary file 1 [file genes-13-01706-s001.zip › Figure S1. Known miRNAs Structure/csi-miR530b-3p_csi-MIR530b.jpg]

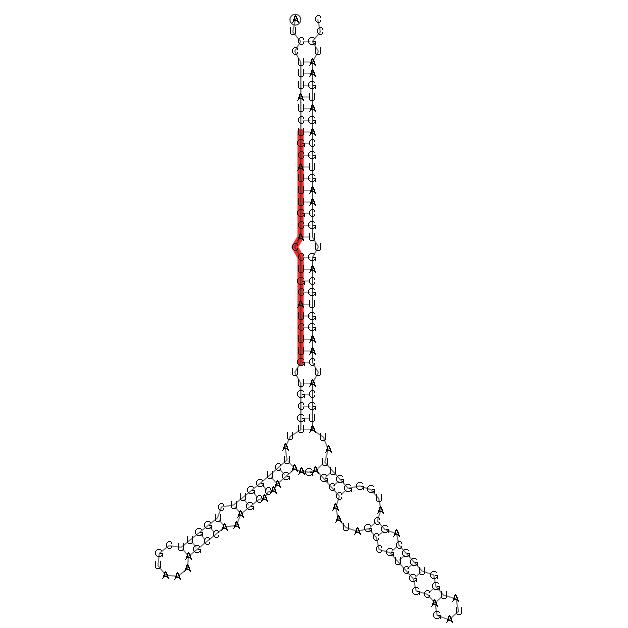

Supplement: Supplementary file 1 [file genes-13-01706-s001.zip › Figure S1. Known miRNAs Structure/csi-miR530b-5p_csi-MIR530b.jpg]

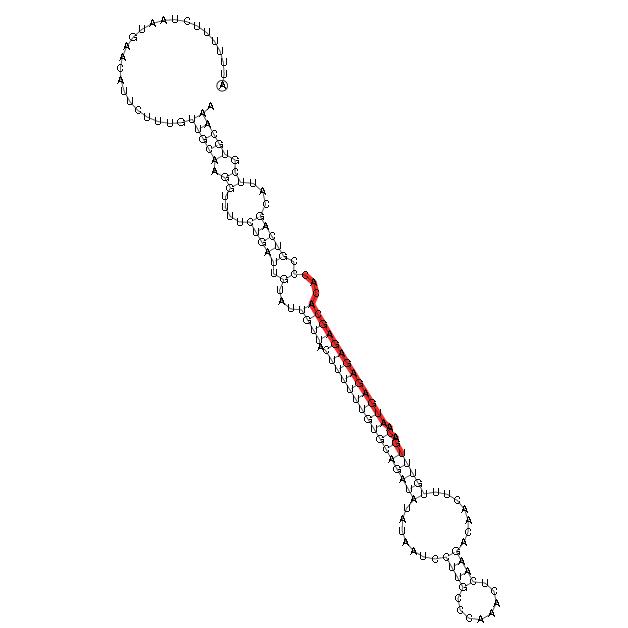

Supplement: Supplementary file 1 [file genes-13-01706-s001.zip › Figure S1. Known miRNAs Structure/csi-miR535a_csi-MIR535a.jpg]

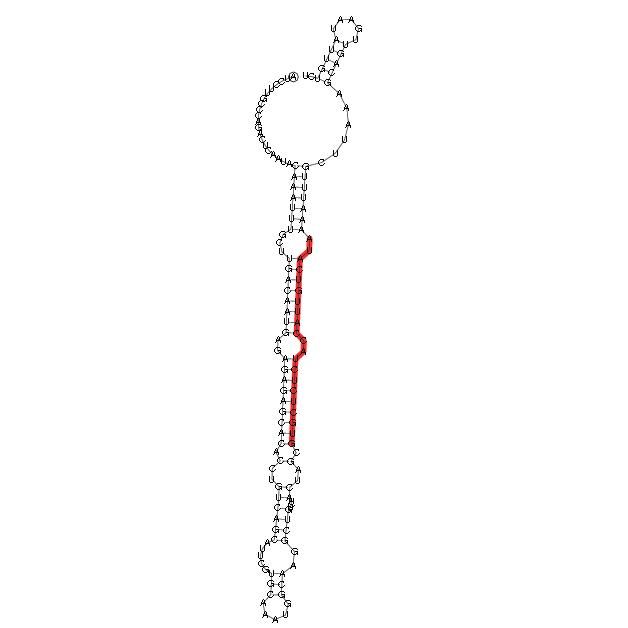

Supplement: Supplementary file 1 [file genes-13-01706-s001.zip › Figure S1. Known miRNAs Structure/csi-miR535b-3p_csi-MIR535b.jpg]

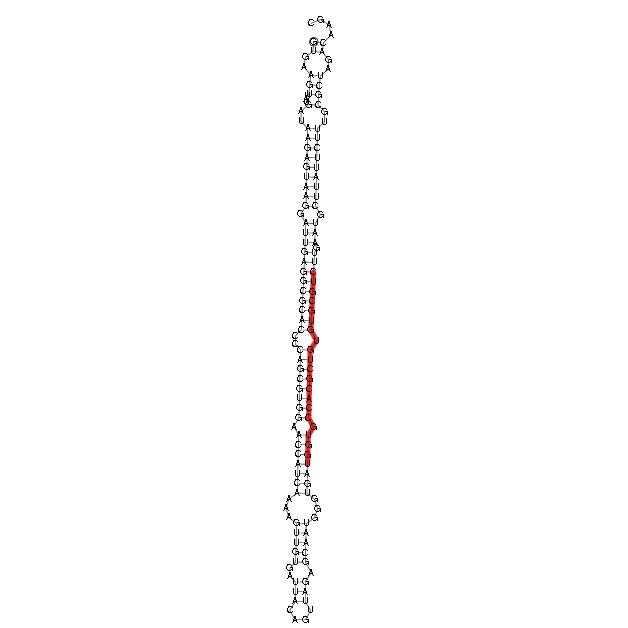

Supplement: Supplementary file 1 [file genes-13-01706-s001.zip › Figure S1. Known miRNAs Structure/csi-miR536-3p_csi-MIR536.jpg]

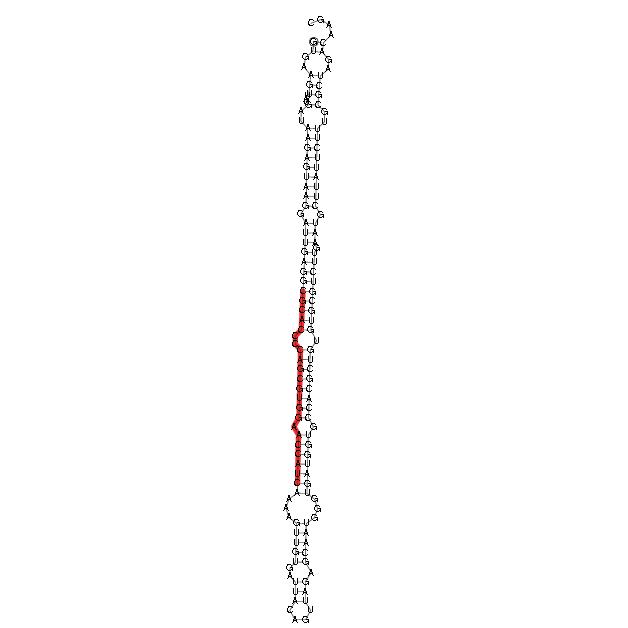

Supplement: Supplementary file 1 [file genes-13-01706-s001.zip › Figure S1. Known miRNAs Structure/csi-miR536-5p_csi-MIR536.jpg]

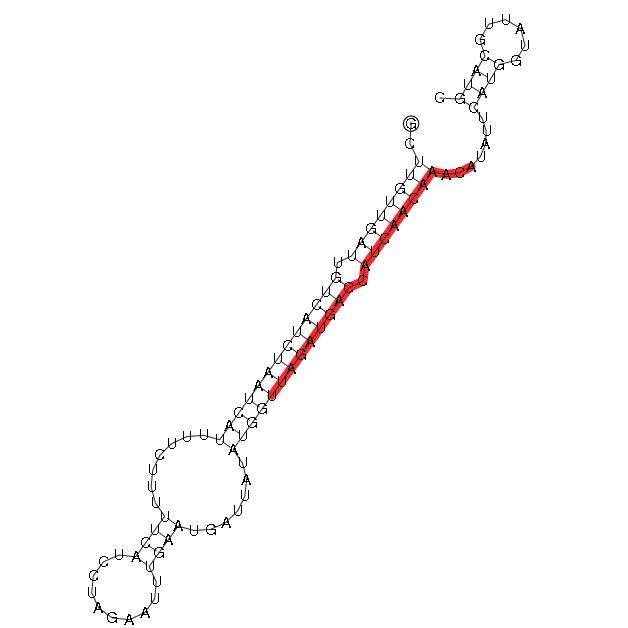

Supplement: Supplementary file 1 [file genes-13-01706-s001.zip › Figure S1. Known miRNAs Structure/csi-miR827_csi-MIR827.jpg]

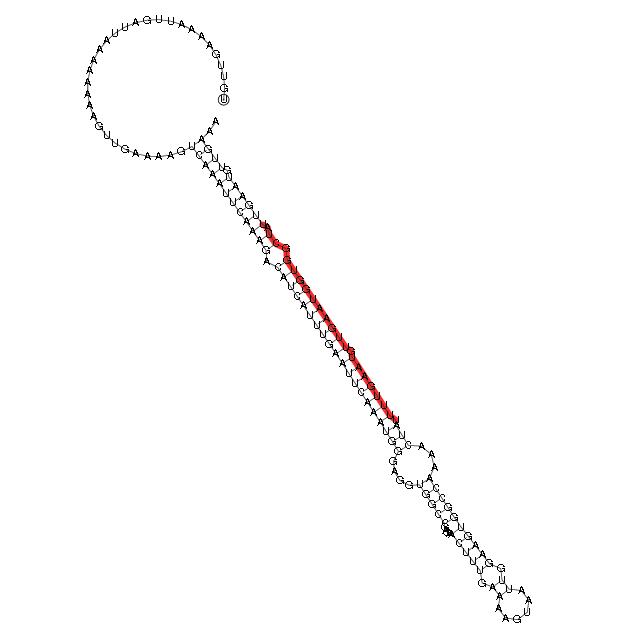

Supplement: Supplementary file 1 [file genes-13-01706-s001.zip › Figure S1. Known miRNAs Structure/csi-miR857_csi-MIR857.jpg]

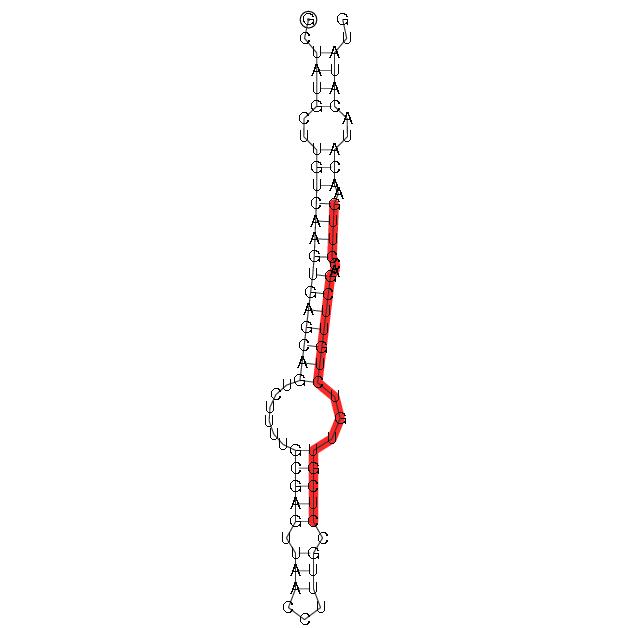

Supplement: Supplementary file 1 [file genes-13-01706-s001.zip › Figure S1. Known miRNAs Structure/csi-miR858-3p_csi-MIR858.jpg]

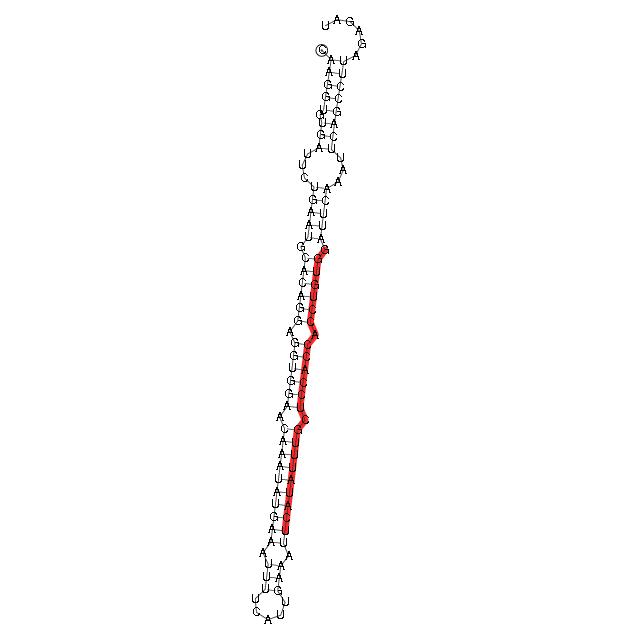

Supplement: Supplementary file 1 [file genes-13-01706-s001.zip › Figure S1. Known miRNAs Structure/csi-miR9560-3p_csi-MIR9560.jpg]

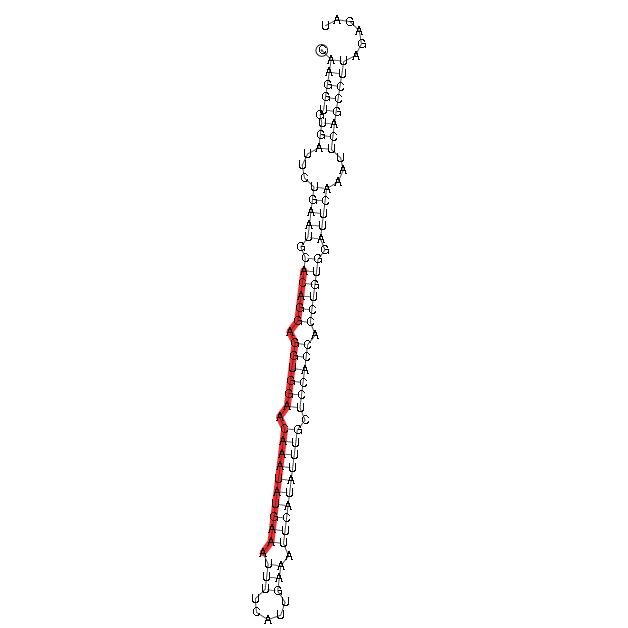

Supplement: Supplementary file 1 [file genes-13-01706-s001.zip › Figure S1. Known miRNAs Structure/csi-miR9560-5p_csi-MIR9560.jpg]

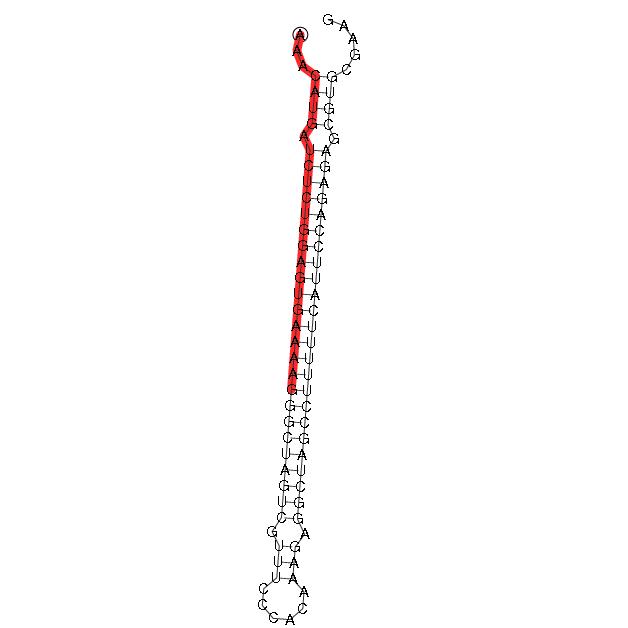

Supplement: Supplementary file 1 [file genes-13-01706-s001.zip › Figure S2. Novel miRNAs Structure/novel_100_novel_100.jpg]

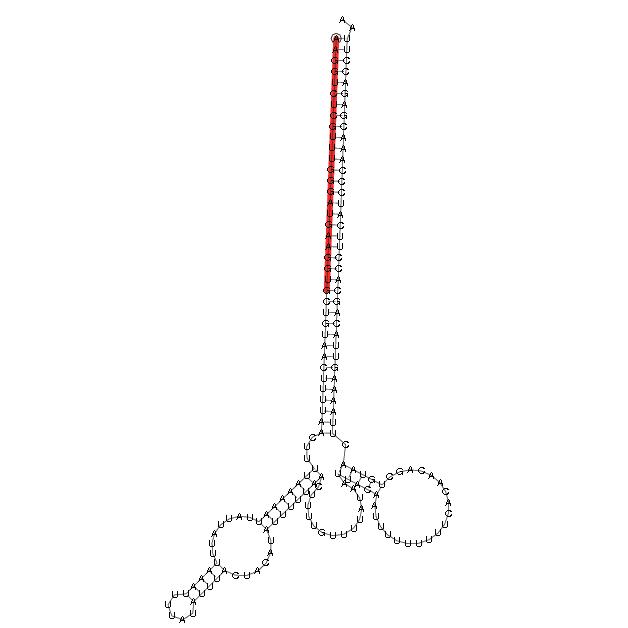

Supplement: Supplementary file 1 [file genes-13-01706-s001.zip › Figure S2. Novel miRNAs Structure/novel_102_novel_102.jpg]

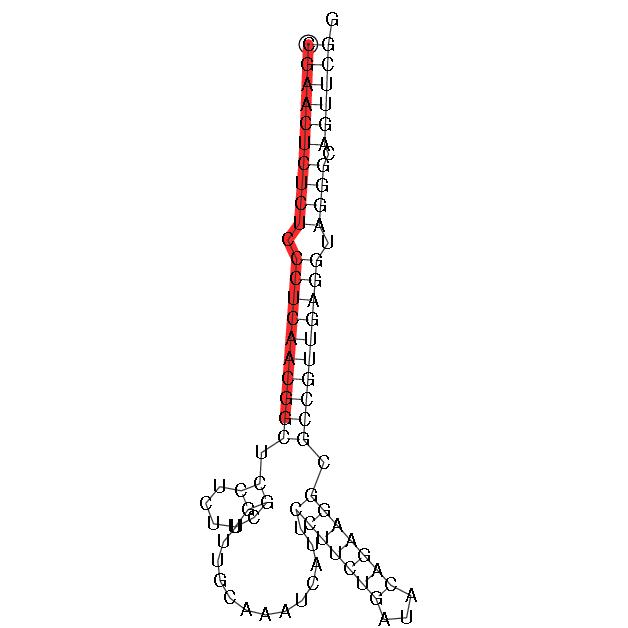

Supplement: Supplementary file 1 [file genes-13-01706-s001.zip › Figure S2. Novel miRNAs Structure/novel_105_novel_105.jpg]

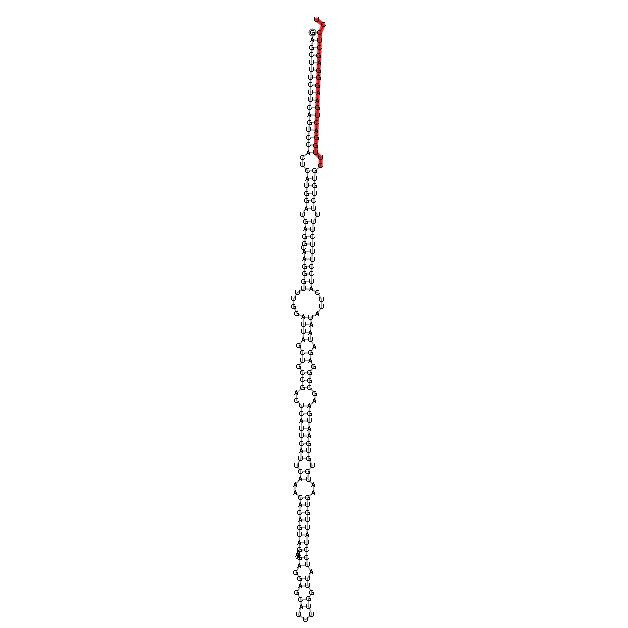

Supplement: Supplementary file 1 [file genes-13-01706-s001.zip › Figure S2. Novel miRNAs Structure/novel_106_novel_106.jpg]

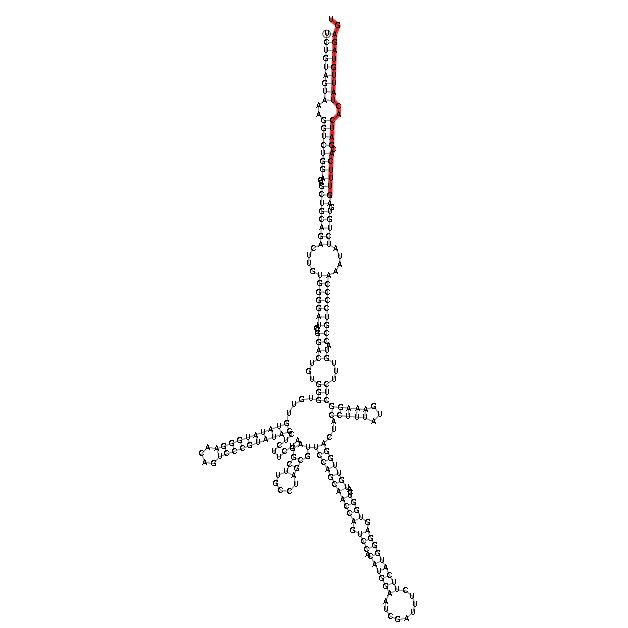

Supplement: Supplementary file 1 [file genes-13-01706-s001.zip › Figure S2. Novel miRNAs Structure/novel_107_novel_107.jpg]

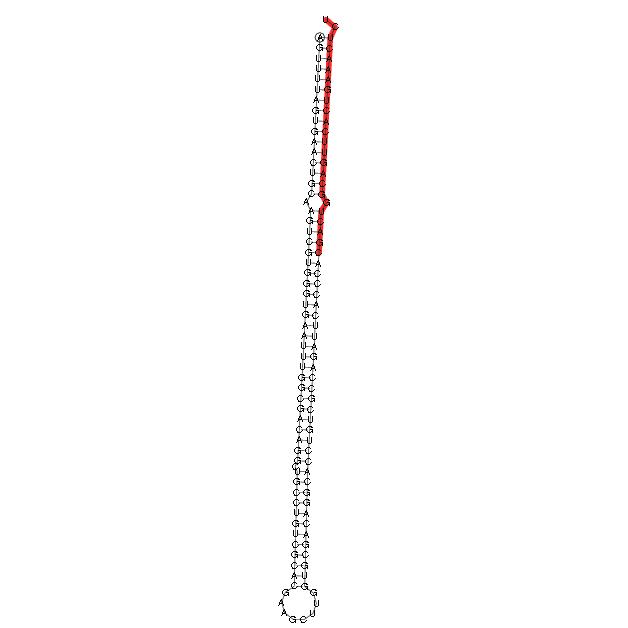

Supplement: Supplementary file 1 [file genes-13-01706-s001.zip › Figure S2. Novel miRNAs Structure/novel_10_novel_10.jpg]

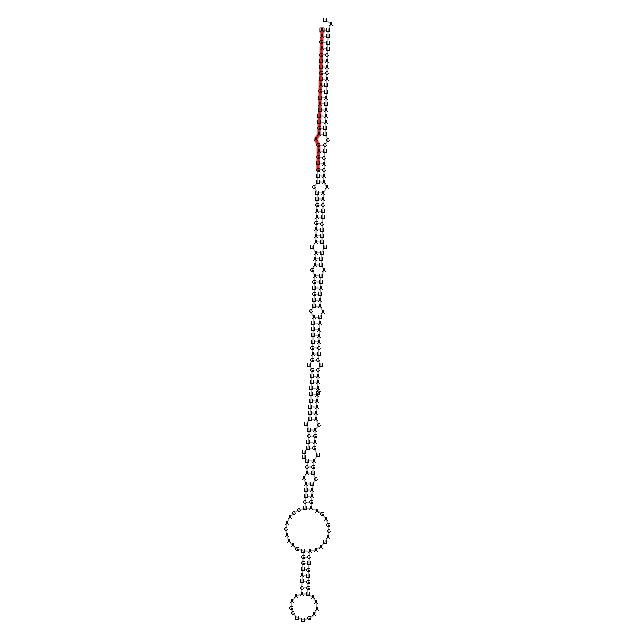

Supplement: Supplementary file 1 [file genes-13-01706-s001.zip › Figure S2. Novel miRNAs Structure/novel_110_novel_110.jpg]

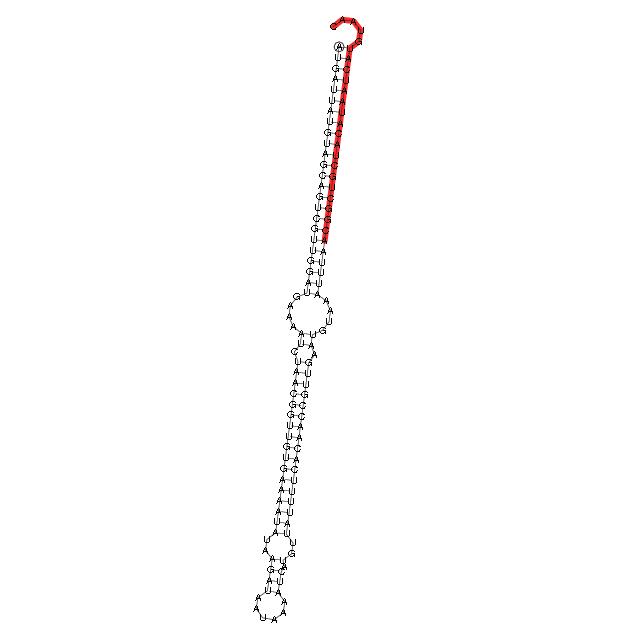

Supplement: Supplementary file 1 [file genes-13-01706-s001.zip › Figure S2. Novel miRNAs Structure/novel_112_novel_112.jpg]

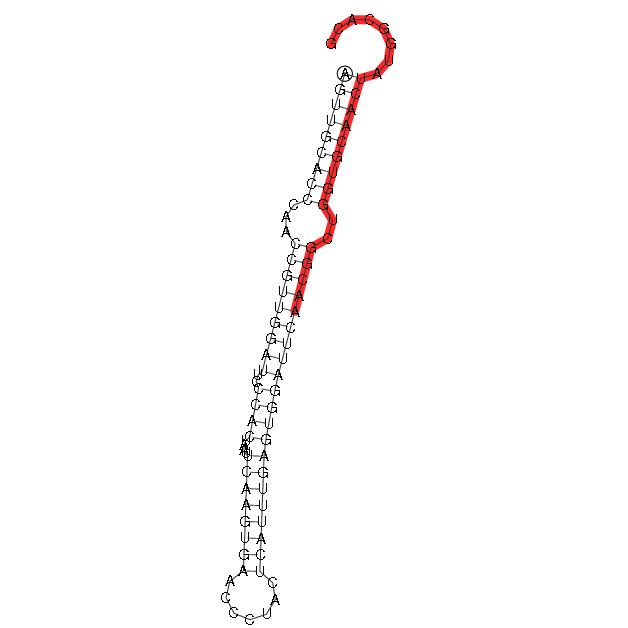

Supplement: Supplementary file 1 [file genes-13-01706-s001.zip › Figure S2. Novel miRNAs Structure/novel_114_novel_114.jpg]

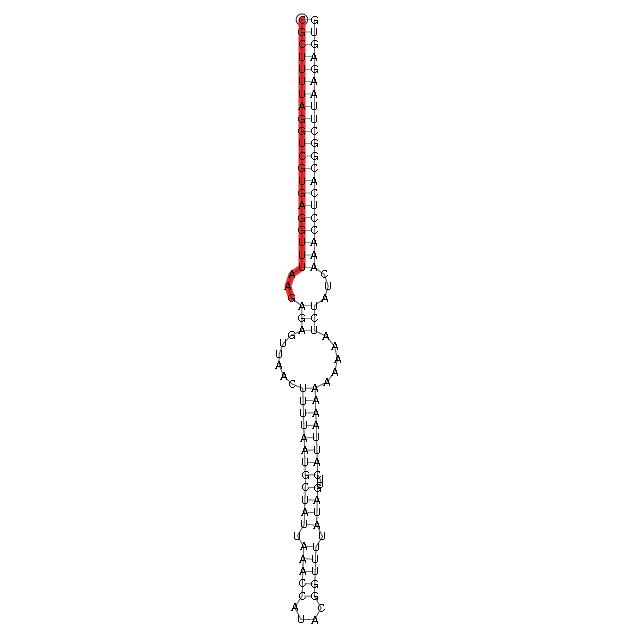

Supplement: Supplementary file 1 [file genes-13-01706-s001.zip › Figure S2. Novel miRNAs Structure/novel_115_novel_115.jpg]

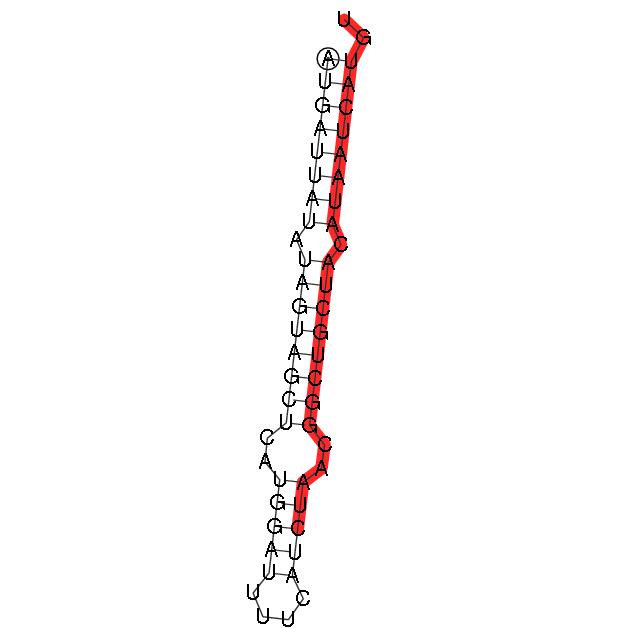

Supplement: Supplementary file 1 [file genes-13-01706-s001.zip › Figure S2. Novel miRNAs Structure/novel_117_novel_117.jpg]

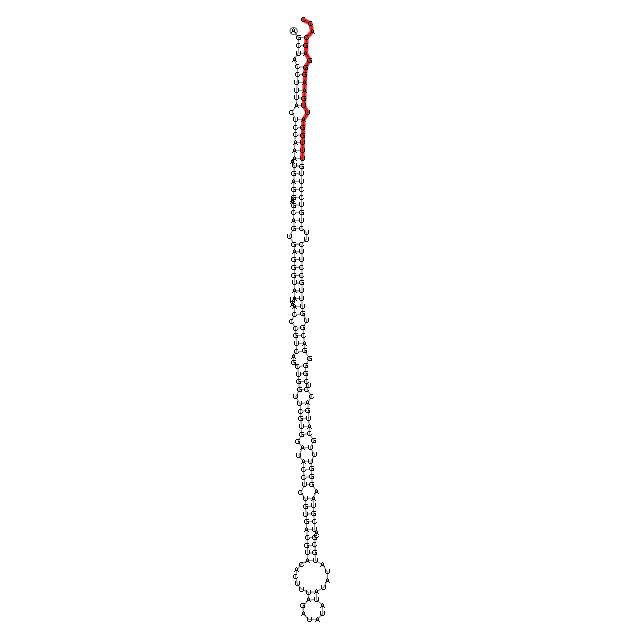

Supplement: Supplementary file 1 [file genes-13-01706-s001.zip › Figure S2. Novel miRNAs Structure/novel_118_novel_118.jpg]

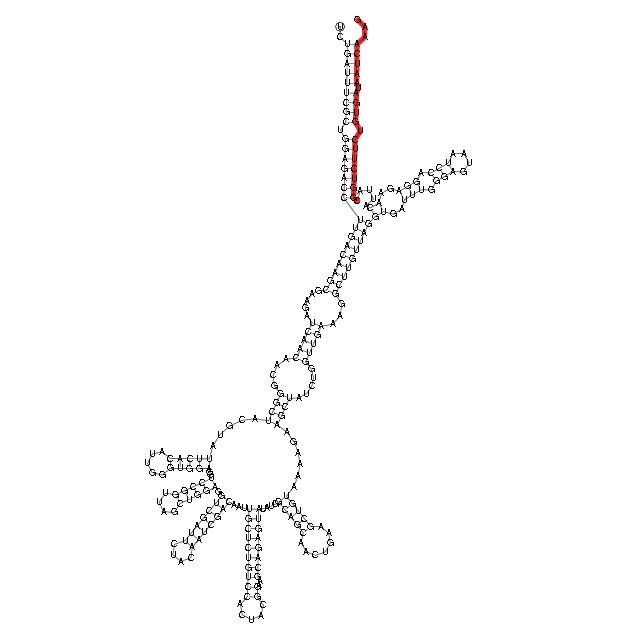

Supplement: Supplementary file 1 [file genes-13-01706-s001.zip › Figure S2. Novel miRNAs Structure/novel_11_novel_11.jpg]

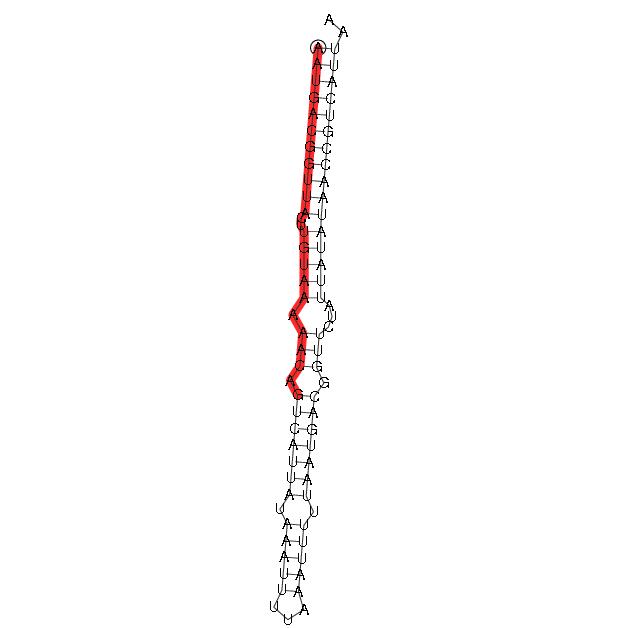

Supplement: Supplementary file 1 [file genes-13-01706-s001.zip › Figure S2. Novel miRNAs Structure/novel_120_novel_120.jpg]

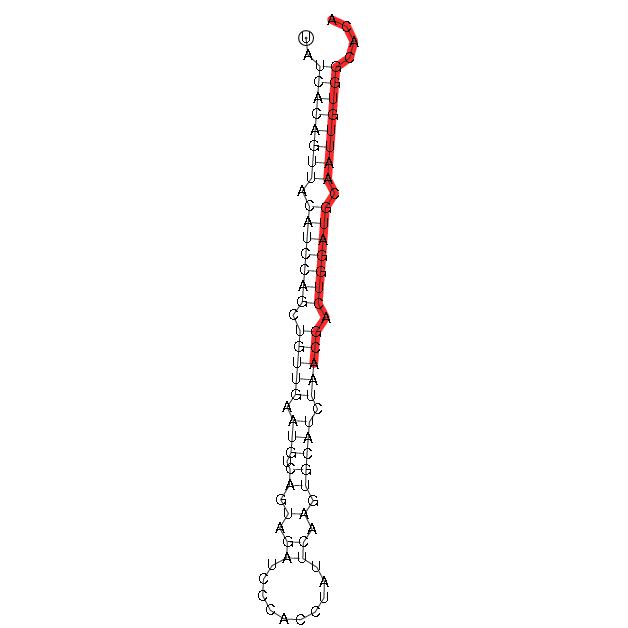

Supplement: Supplementary file 1 [file genes-13-01706-s001.zip › Figure S2. Novel miRNAs Structure/novel_121_novel_121.jpg]

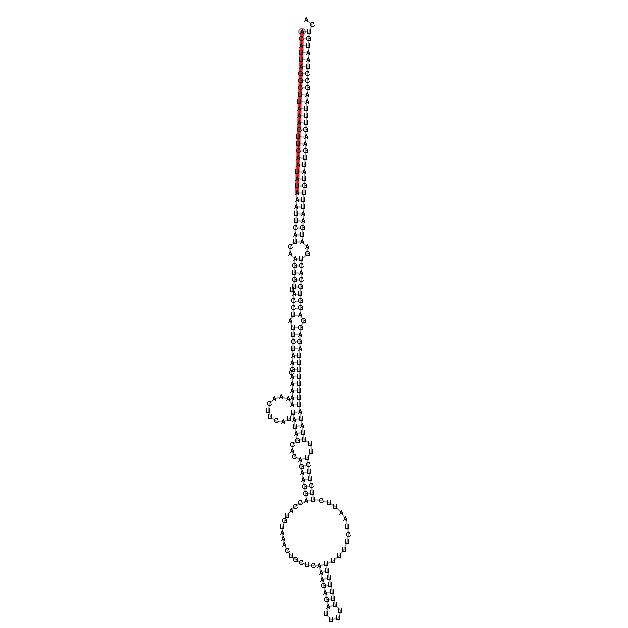

Supplement: Supplementary file 1 [file genes-13-01706-s001.zip › Figure S2. Novel miRNAs Structure/novel_127_novel_127.jpg]

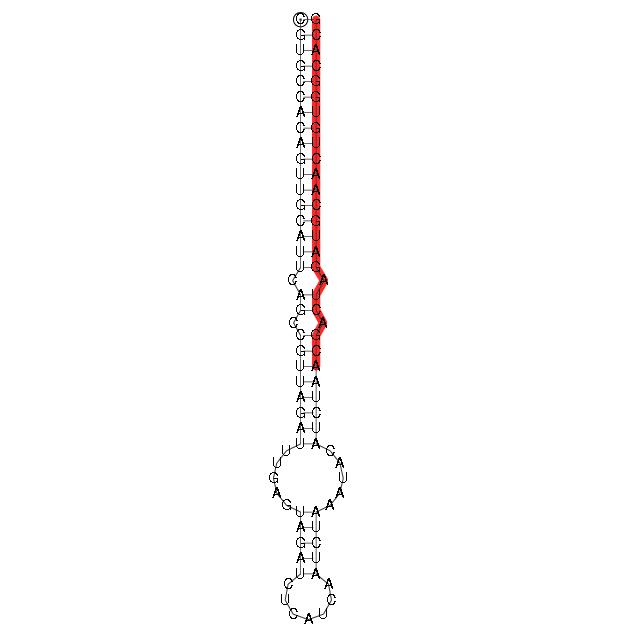

Supplement: Supplementary file 1 [file genes-13-01706-s001.zip › Figure S2. Novel miRNAs Structure/novel_128_novel_128.jpg]

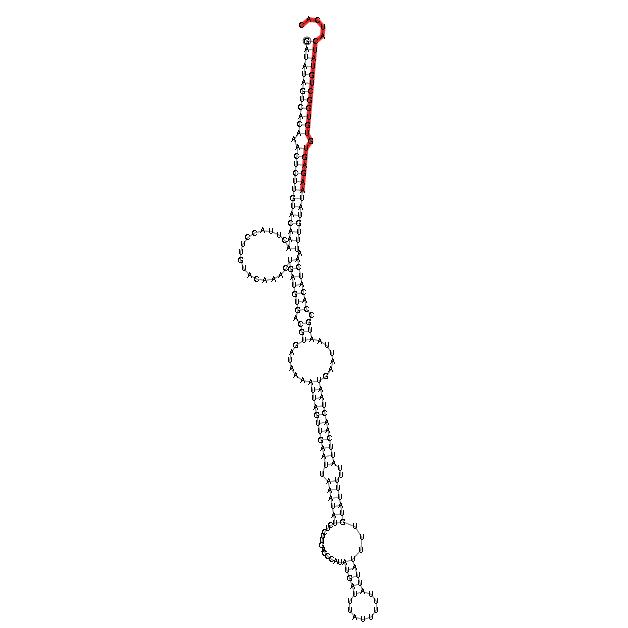

Supplement: Supplementary file 1 [file genes-13-01706-s001.zip › Figure S2. Novel miRNAs Structure/novel_129_novel_129.jpg]

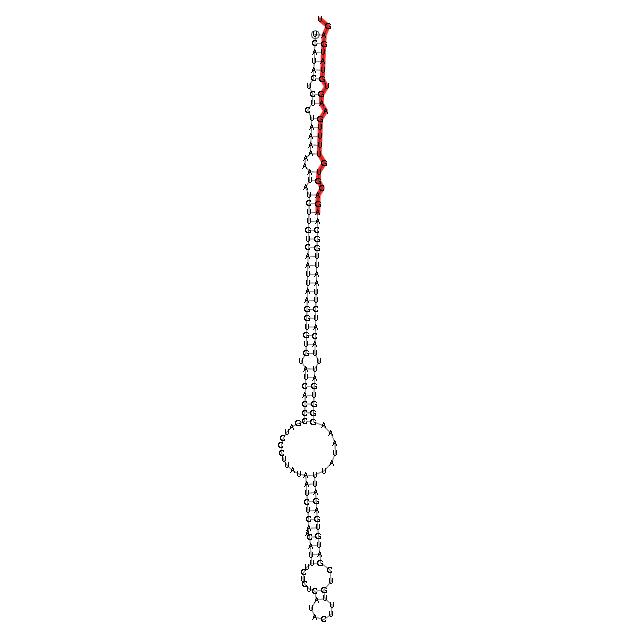

Supplement: Supplementary file 1 [file genes-13-01706-s001.zip › Figure S2. Novel miRNAs Structure/novel_133_novel_133.jpg]

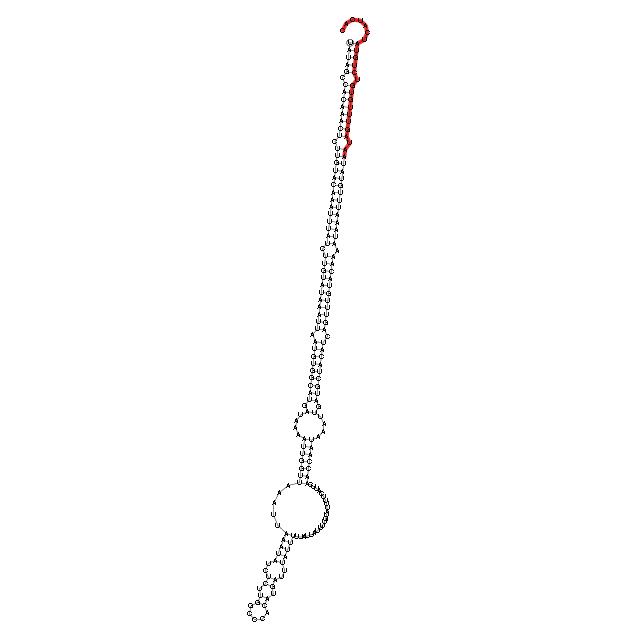

Supplement: Supplementary file 1 [file genes-13-01706-s001.zip › Figure S2. Novel miRNAs Structure/novel_134_novel_134.jpg]

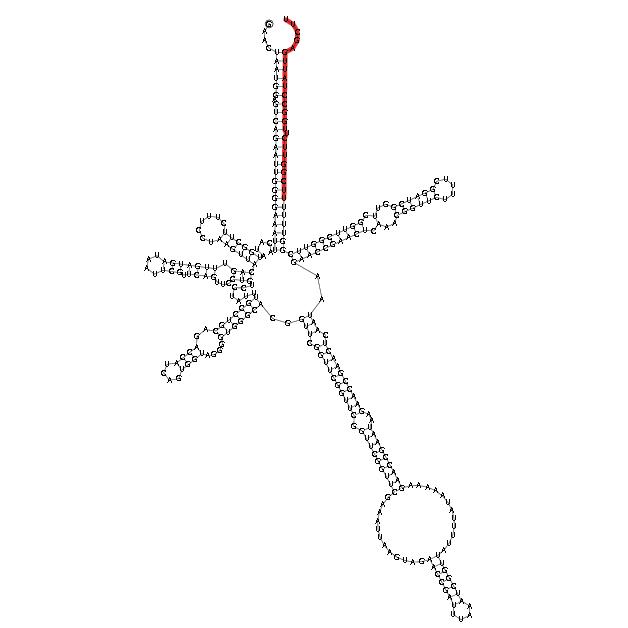

Supplement: Supplementary file 1 [file genes-13-01706-s001.zip › Figure S2. Novel miRNAs Structure/novel_135_novel_135.jpg]

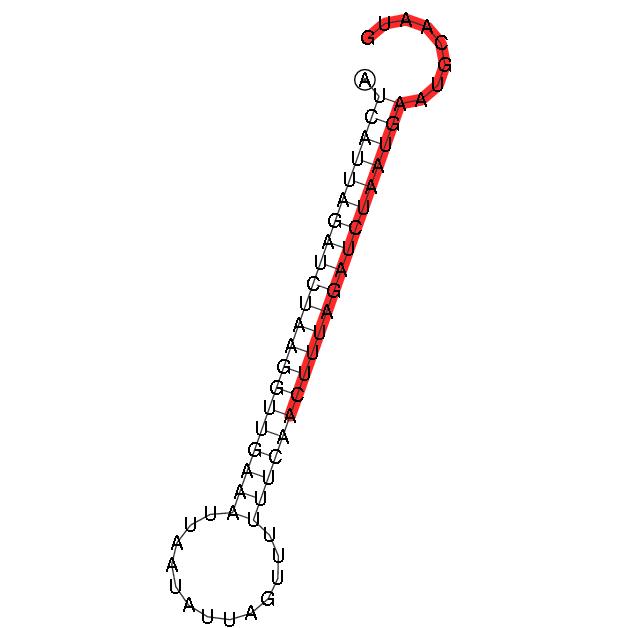

Supplement: Supplementary file 1 [file genes-13-01706-s001.zip › Figure S2. Novel miRNAs Structure/novel_136_novel_136.jpg]

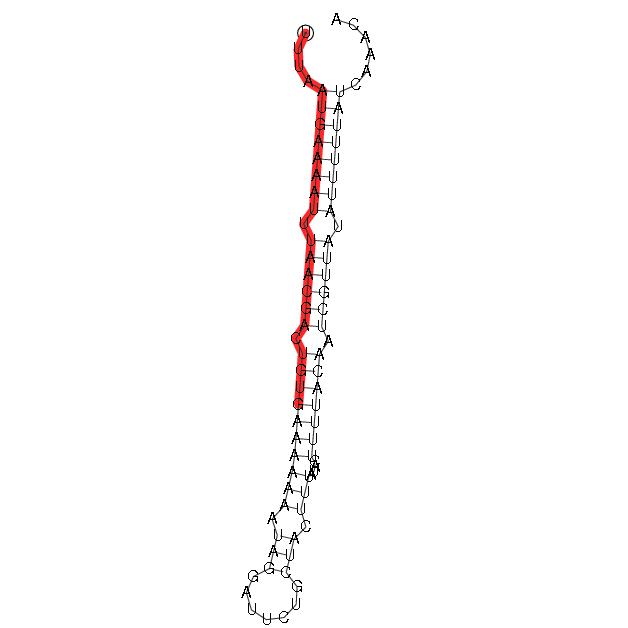

Supplement: Supplementary file 1 [file genes-13-01706-s001.zip › Figure S2. Novel miRNAs Structure/novel_137_novel_137.jpg]

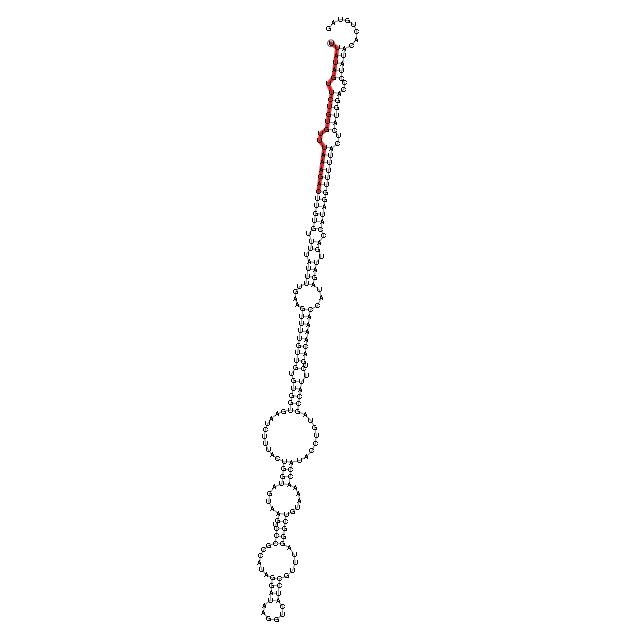

Supplement: Supplementary file 1 [file genes-13-01706-s001.zip › Figure S2. Novel miRNAs Structure/novel_139_novel_139.jpg]

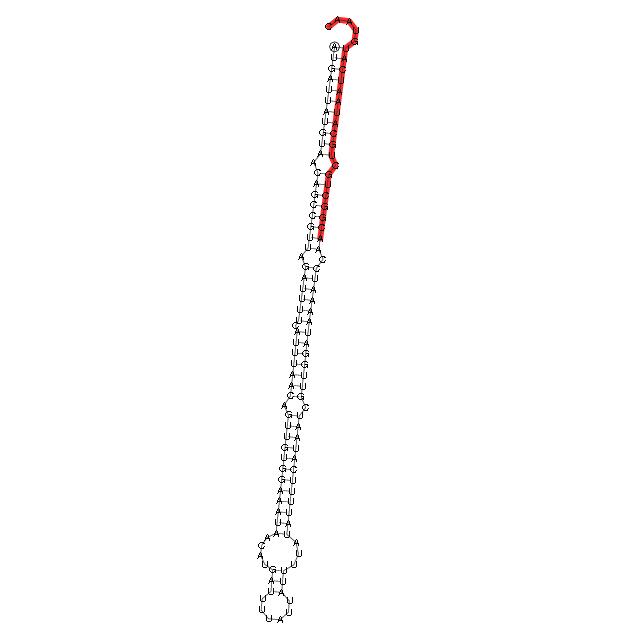

Supplement: Supplementary file 1 [file genes-13-01706-s001.zip › Figure S2. Novel miRNAs Structure/novel_141_novel_141.jpg]

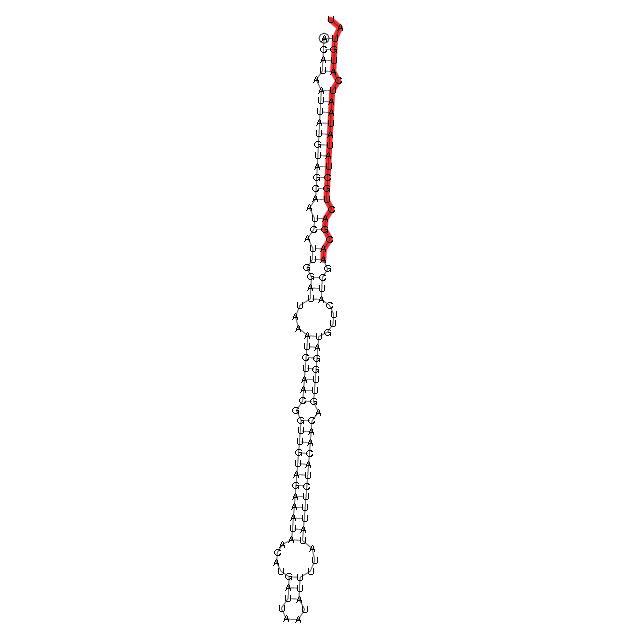

Supplement: Supplementary file 1 [file genes-13-01706-s001.zip › Figure S2. Novel miRNAs Structure/novel_143_novel_143.jpg]

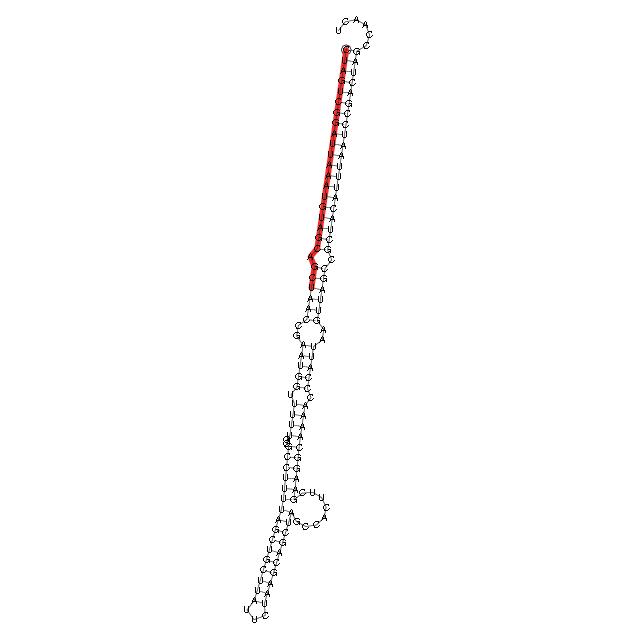

Supplement: Supplementary file 1 [file genes-13-01706-s001.zip › Figure S2. Novel miRNAs Structure/novel_144_novel_144.jpg]

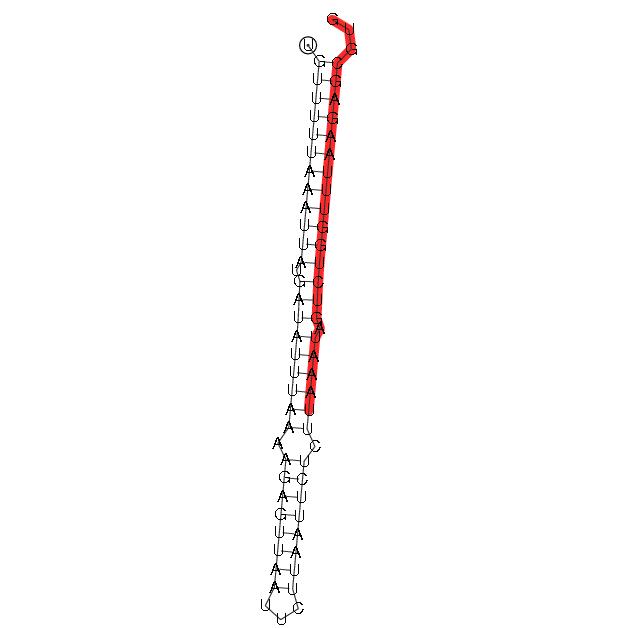

Supplement: Supplementary file 1 [file genes-13-01706-s001.zip › Figure S2. Novel miRNAs Structure/novel_146_novel_146.jpg]

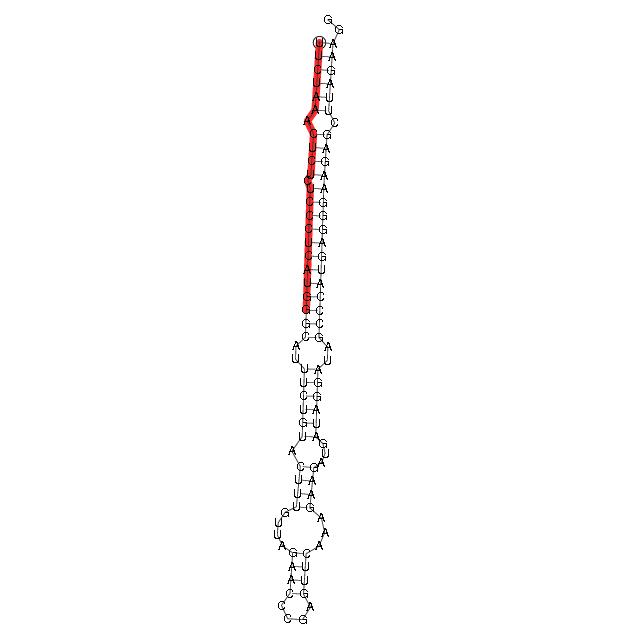

Supplement: Supplementary file 1 [file genes-13-01706-s001.zip › Figure S2. Novel miRNAs Structure/novel_16_novel_16.jpg]

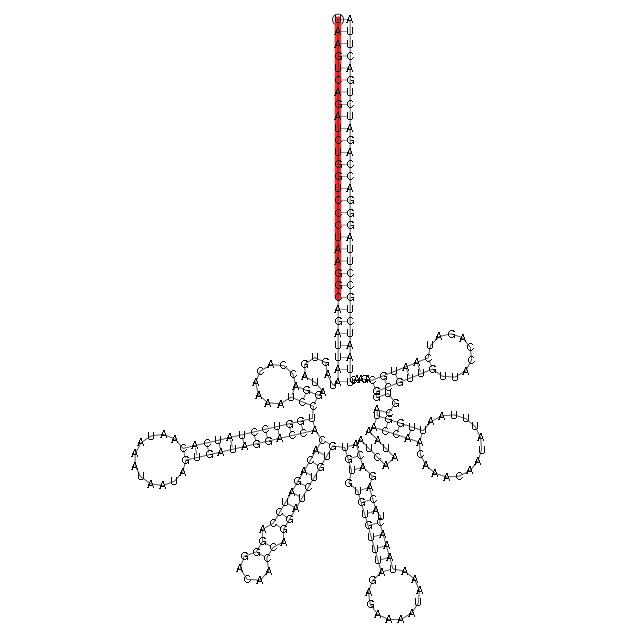

Supplement: Supplementary file 1 [file genes-13-01706-s001.zip › Figure S2. Novel miRNAs Structure/novel_17_novel_17.jpg]

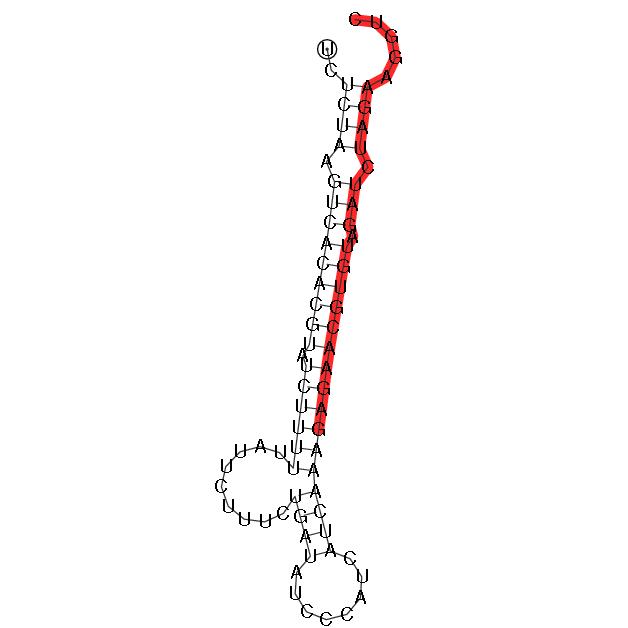

Supplement: Supplementary file 1 [file genes-13-01706-s001.zip › Figure S2. Novel miRNAs Structure/novel_18_novel_18.jpg]

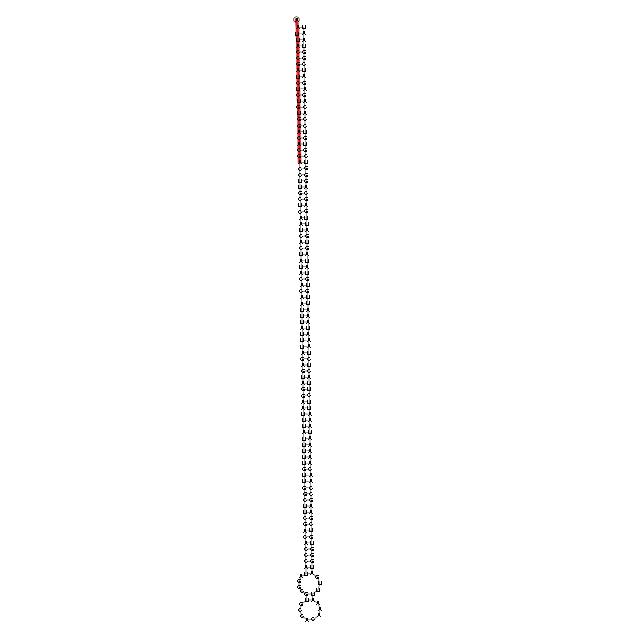

Supplement: Supplementary file 1 [file genes-13-01706-s001.zip › Figure S2. Novel miRNAs Structure/novel_1_novel_1.jpg]

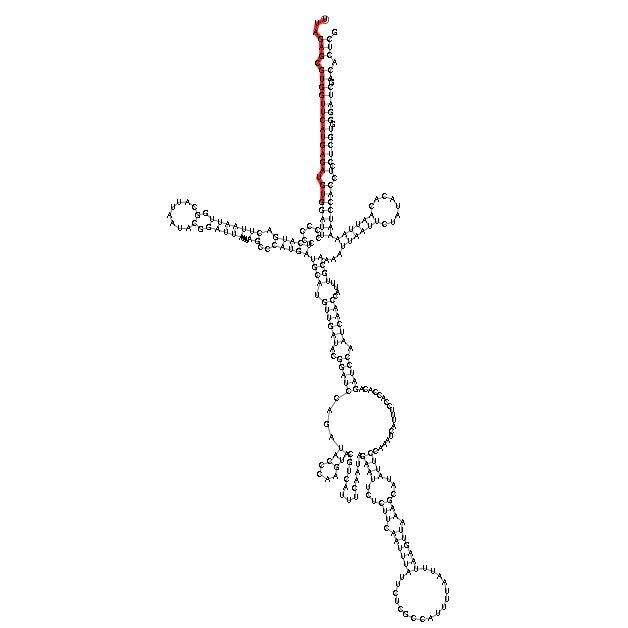

Supplement: Supplementary file 1 [file genes-13-01706-s001.zip › Figure S2. Novel miRNAs Structure/novel_22_novel_22.jpg]
