# Supplementary figures and images for: Comparative Transcriptome and sRNAome Analyses Reveal the Regulatory Mechanisms of Fruit Ripening in a Spontaneous Early-Ripening Navel Orange Mutant and Its Wild Type (part 1 of 2)
Source: Genes (Basel). 2022 Sep 22;13(10):1706. doi: 10.3390/genes13101706 (PMC9601947; doi:10.3390/genes13101706)

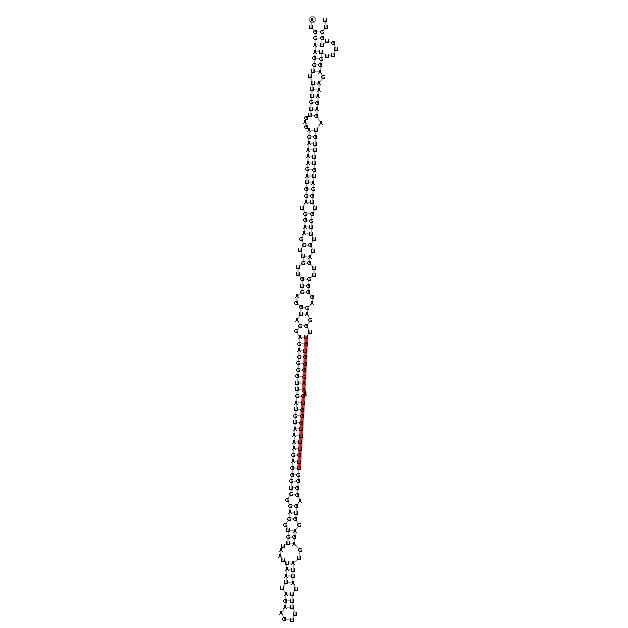

Supplement: Supplementary file 1 [file genes-13-01706-s001.zip › Figure S1. Known miRNAs Structure/csi-miR12105-3p_csi-MIR12105.jpg]

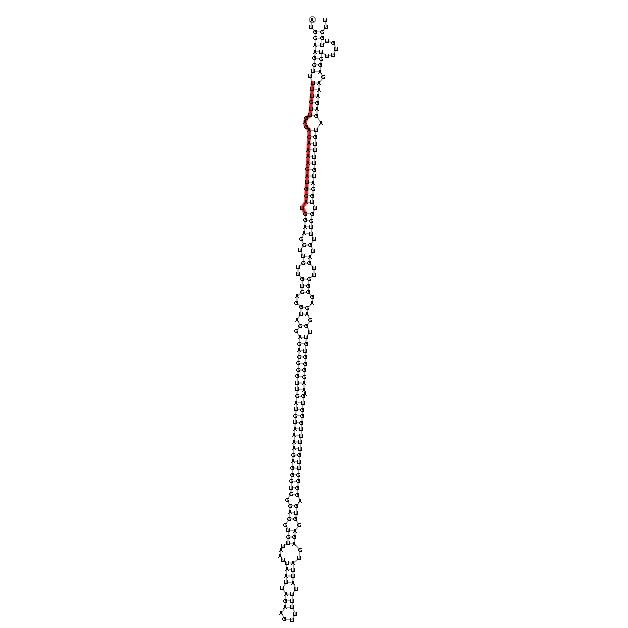

Supplement: Supplementary file 1 [file genes-13-01706-s001.zip › Figure S1. Known miRNAs Structure/csi-miR12105-5p_csi-MIR12105.jpg]

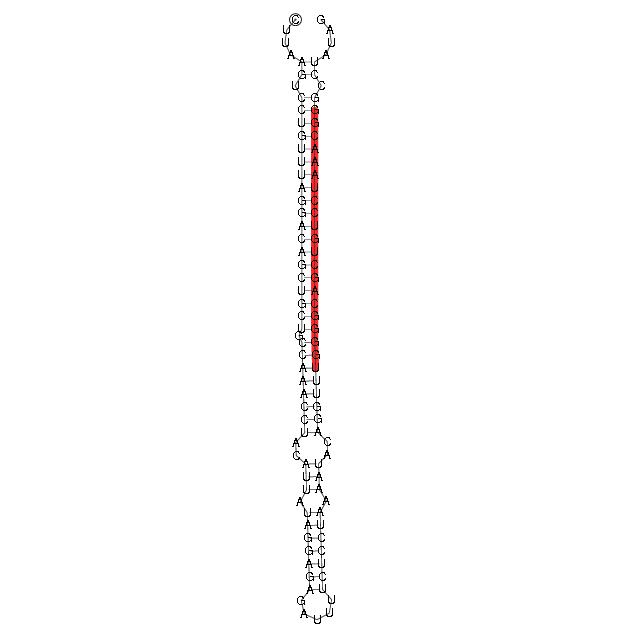

Supplement: Supplementary file 1 [file genes-13-01706-s001.zip › Figure S1. Known miRNAs Structure/csi-miR12106-3p_csi-MIR12106.jpg]

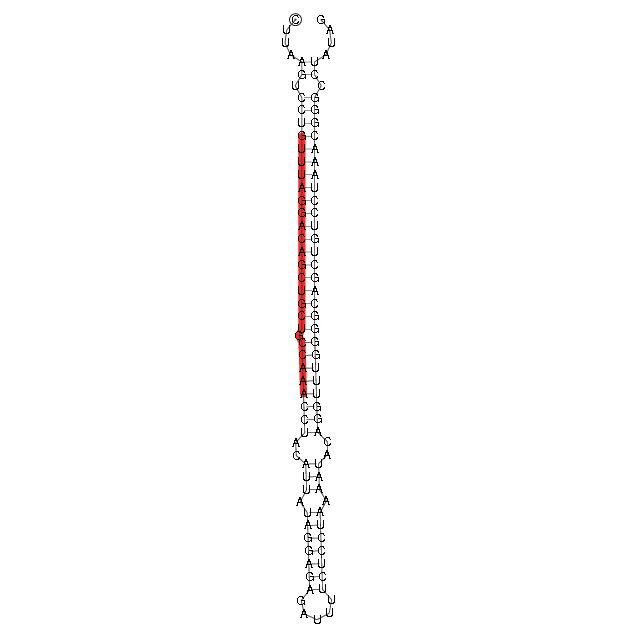

Supplement: Supplementary file 1 [file genes-13-01706-s001.zip › Figure S1. Known miRNAs Structure/csi-miR12106-5p_csi-MIR12106.jpg]

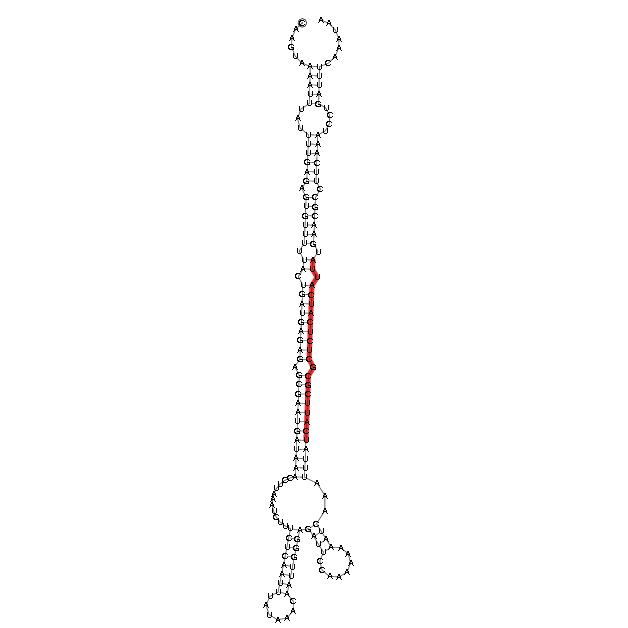

Supplement: Supplementary file 1 [file genes-13-01706-s001.zip › Figure S1. Known miRNAs Structure/csi-miR12107-3p_csi-MIR12107.jpg]

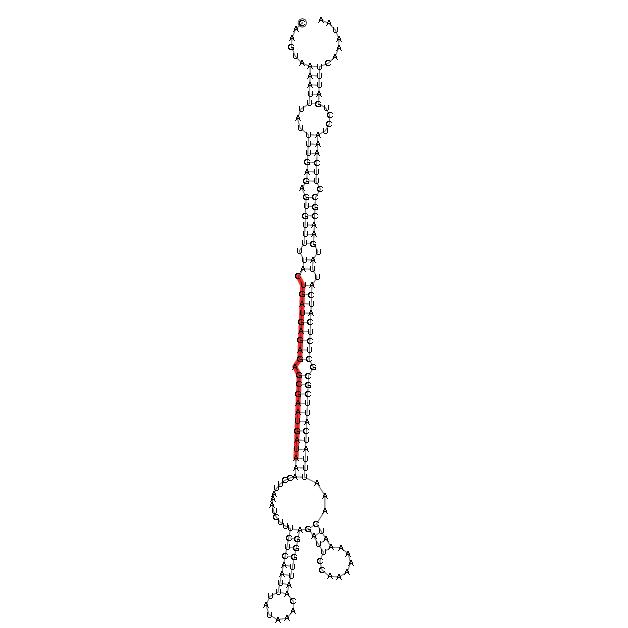

Supplement: Supplementary file 1 [file genes-13-01706-s001.zip › Figure S1. Known miRNAs Structure/csi-miR12107-5p_csi-MIR12107.jpg]

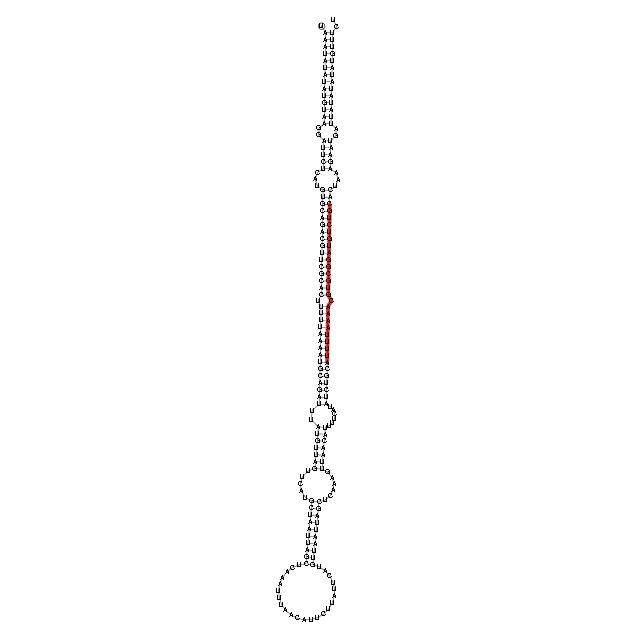

Supplement: Supplementary file 1 [file genes-13-01706-s001.zip › Figure S1. Known miRNAs Structure/csi-miR12108-3p_csi-MIR12108.jpg]

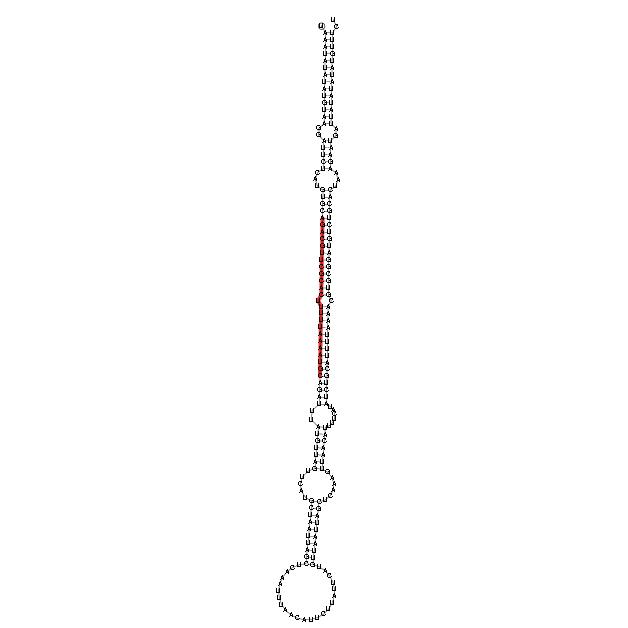

Supplement: Supplementary file 1 [file genes-13-01706-s001.zip › Figure S1. Known miRNAs Structure/csi-miR12108-5p_csi-MIR12108.jpg]

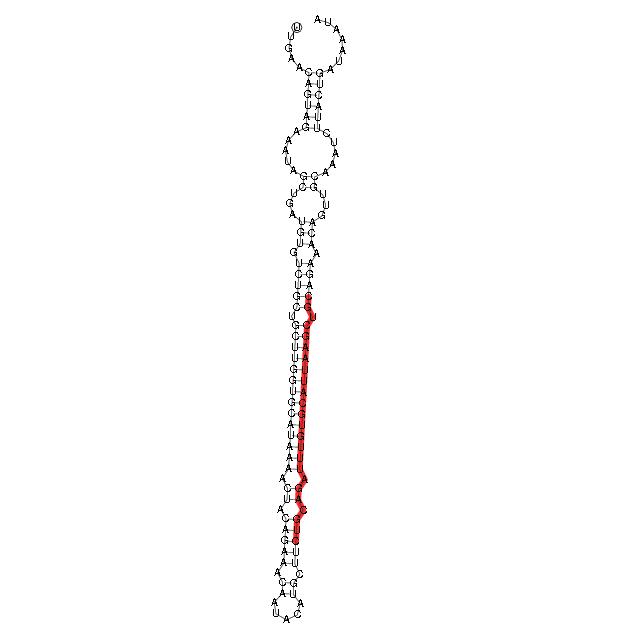

Supplement: Supplementary file 1 [file genes-13-01706-s001.zip › Figure S1. Known miRNAs Structure/csi-miR12109-3p_csi-MIR12109.jpg]

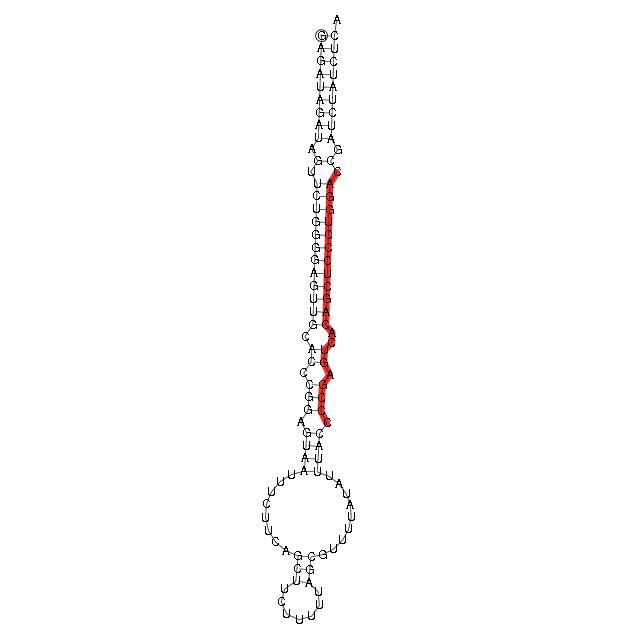

Supplement: Supplementary file 1 [file genes-13-01706-s001.zip › Figure S1. Known miRNAs Structure/csi-miR12110-3p_csi-MIR12110.jpg]

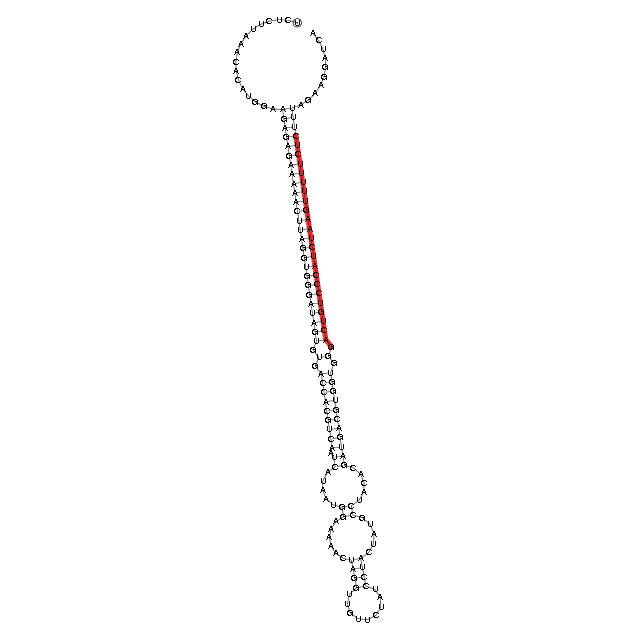

Supplement: Supplementary file 1 [file genes-13-01706-s001.zip › Figure S1. Known miRNAs Structure/csi-miR12111-3p_csi-MIR12111.jpg]

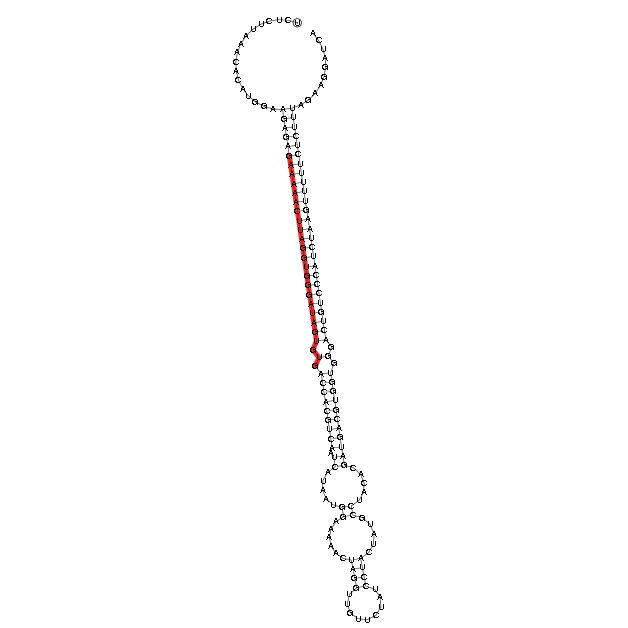

Supplement: Supplementary file 1 [file genes-13-01706-s001.zip › Figure S1. Known miRNAs Structure/csi-miR12111-5p_csi-MIR12111.jpg]

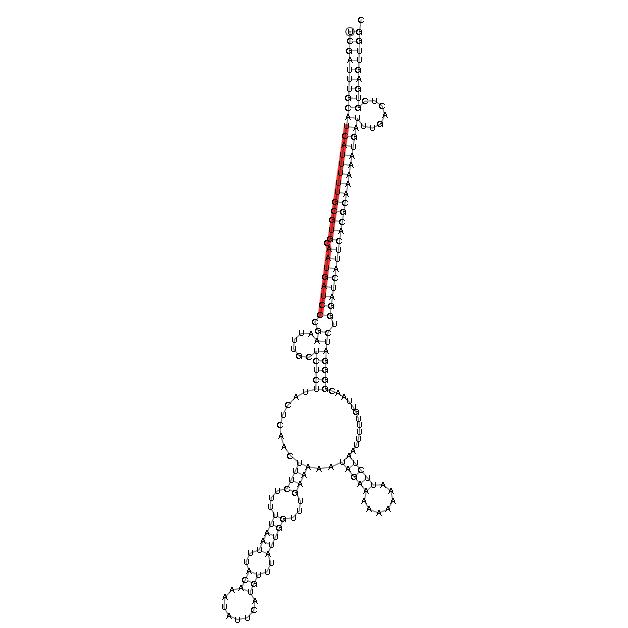

Supplement: Supplementary file 1 [file genes-13-01706-s001.zip › Figure S1. Known miRNAs Structure/csi-miR1515a_csi-MIR1515a.jpg]

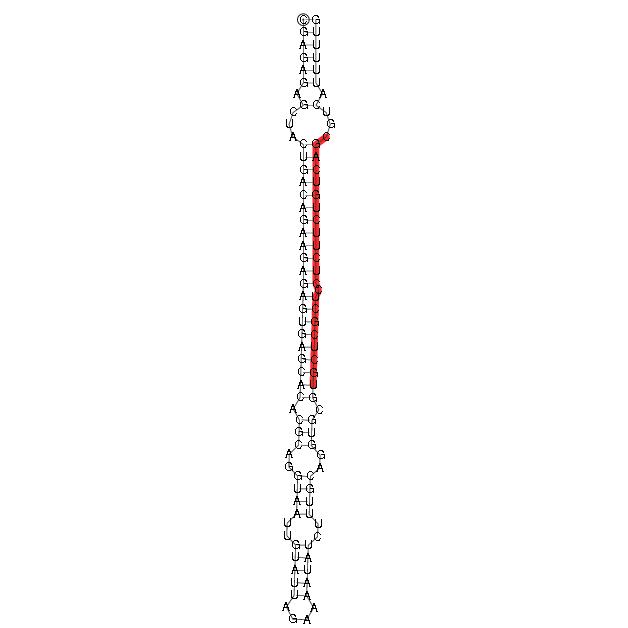

Supplement: Supplementary file 1 [file genes-13-01706-s001.zip › Figure S1. Known miRNAs Structure/csi-miR156a-3p_csi-MIR156a.jpg]

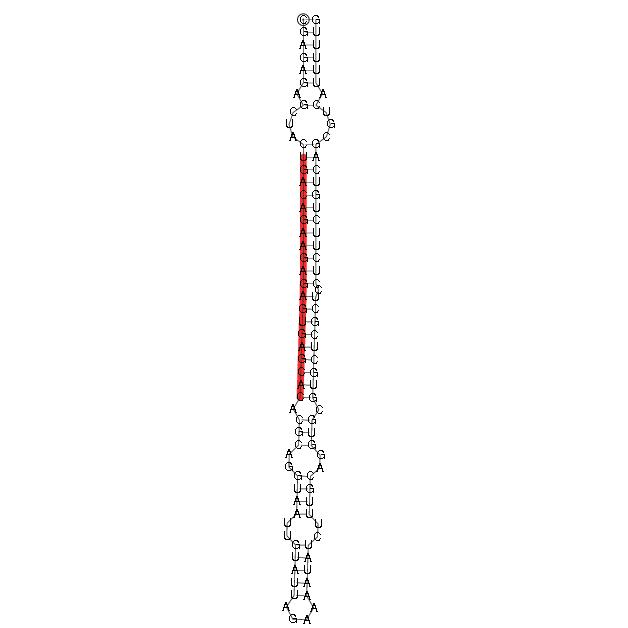

Supplement: Supplementary file 1 [file genes-13-01706-s001.zip › Figure S1. Known miRNAs Structure/csi-miR156a-5p_csi-MIR156a.jpg]

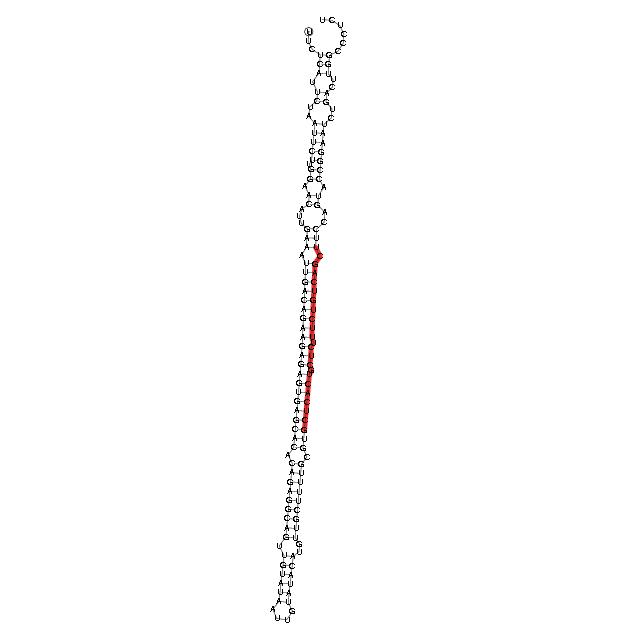

Supplement: Supplementary file 1 [file genes-13-01706-s001.zip › Figure S1. Known miRNAs Structure/csi-miR156b-3p_csi-MIR156b.jpg]

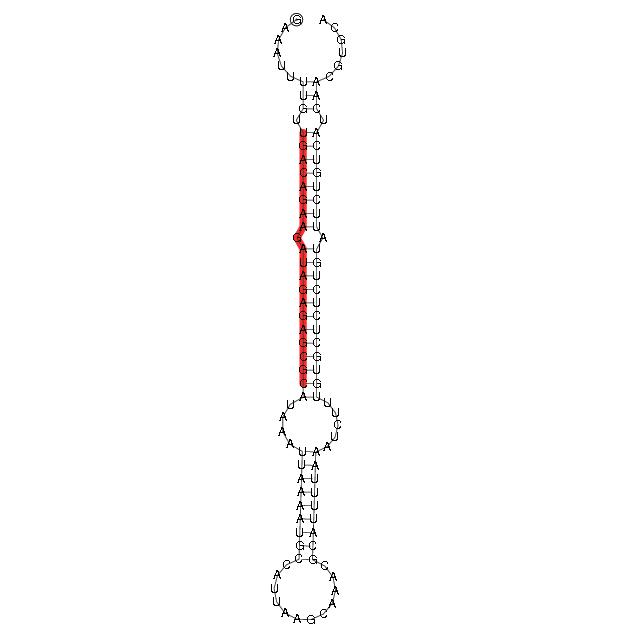

Supplement: Supplementary file 1 [file genes-13-01706-s001.zip › Figure S1. Known miRNAs Structure/csi-miR156d-5p_csi-MIR156d.jpg]

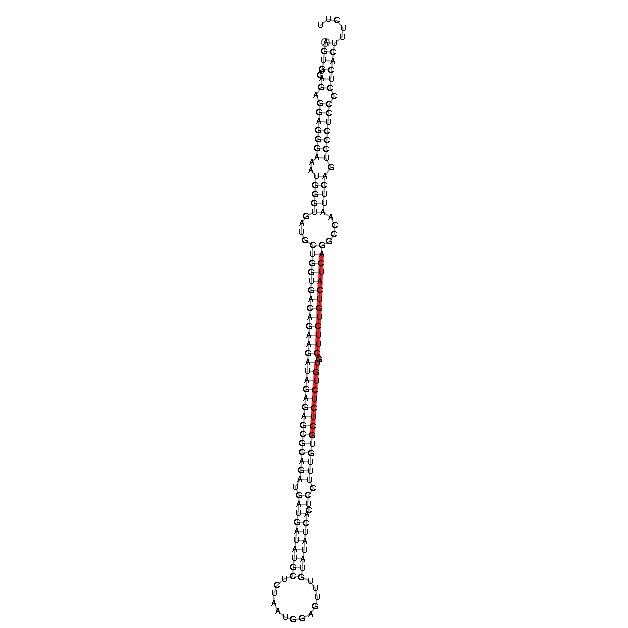

Supplement: Supplementary file 1 [file genes-13-01706-s001.zip › Figure S1. Known miRNAs Structure/csi-miR156e-3p_csi-MIR156e.jpg]

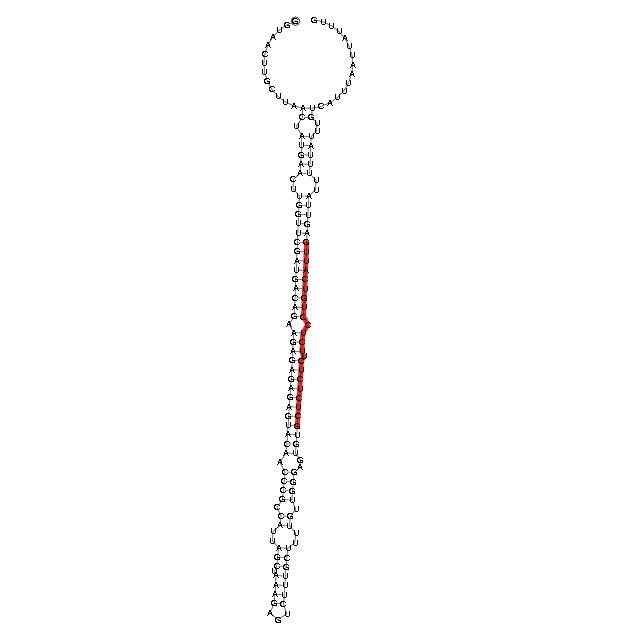

Supplement: Supplementary file 1 [file genes-13-01706-s001.zip › Figure S1. Known miRNAs Structure/csi-miR156f-3p_csi-MIR156f.jpg]

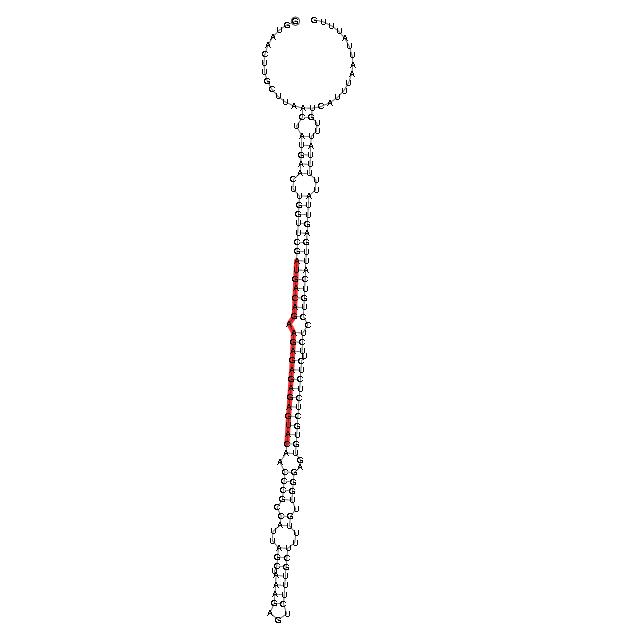

Supplement: Supplementary file 1 [file genes-13-01706-s001.zip › Figure S1. Known miRNAs Structure/csi-miR156f-5p_csi-MIR156f.jpg]

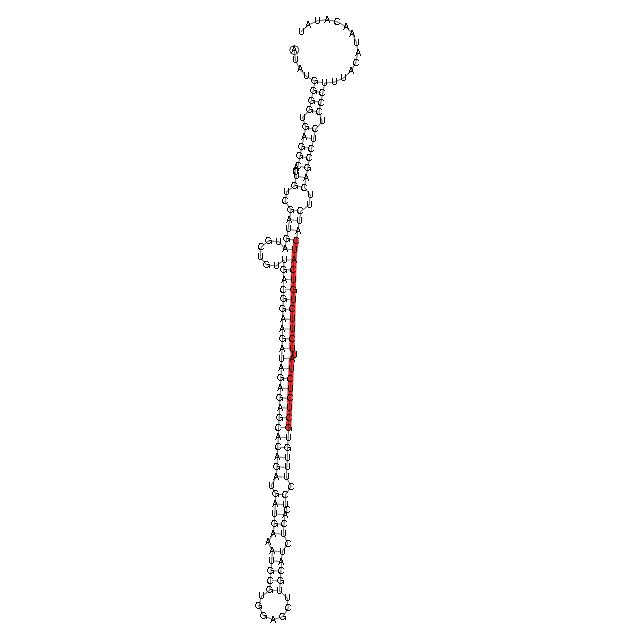

Supplement: Supplementary file 1 [file genes-13-01706-s001.zip › Figure S1. Known miRNAs Structure/csi-miR156g-3p_csi-MIR156g.jpg]

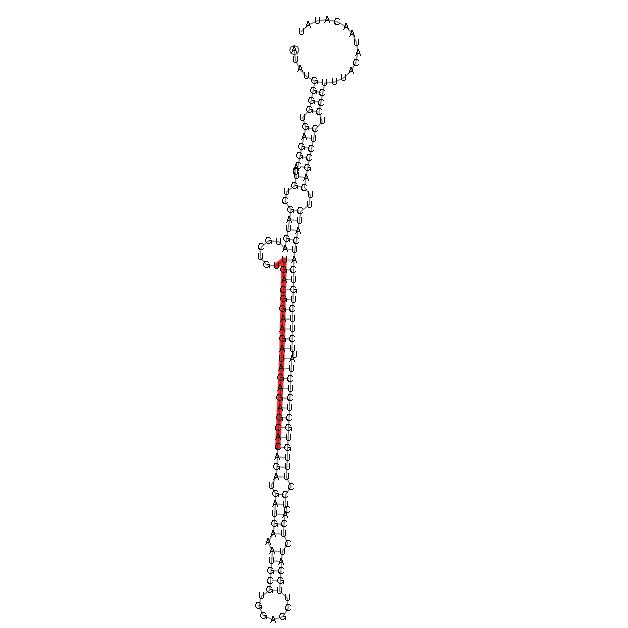

Supplement: Supplementary file 1 [file genes-13-01706-s001.zip › Figure S1. Known miRNAs Structure/csi-miR156g-5p_csi-MIR156g.jpg]

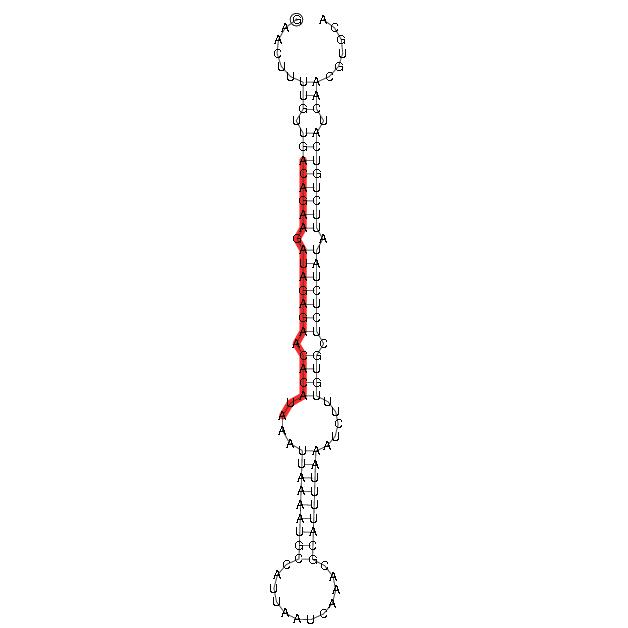

Supplement: Supplementary file 1 [file genes-13-01706-s001.zip › Figure S1. Known miRNAs Structure/csi-miR156h-5p_csi-MIR156h.jpg]

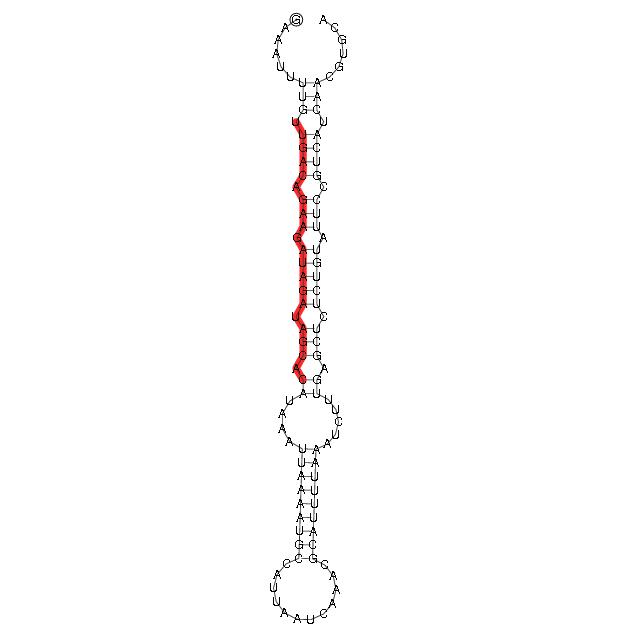

Supplement: Supplementary file 1 [file genes-13-01706-s001.zip › Figure S1. Known miRNAs Structure/csi-miR156i-5p_csi-MIR156i.jpg]

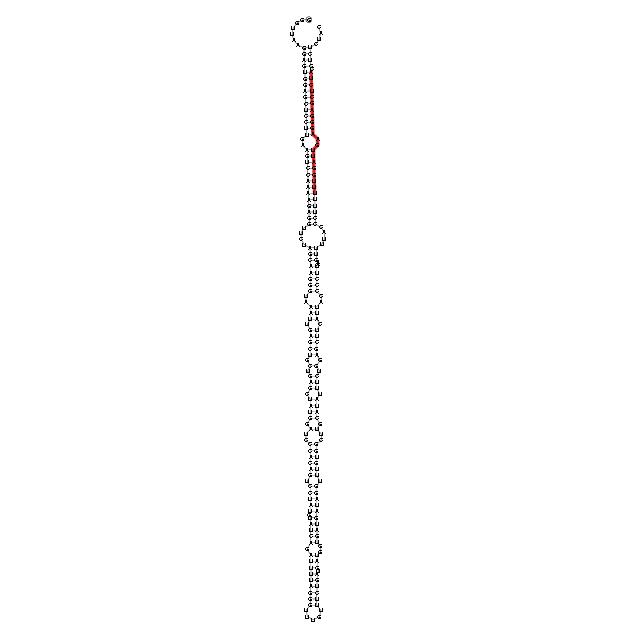

Supplement: Supplementary file 1 [file genes-13-01706-s001.zip › Figure S1. Known miRNAs Structure/csi-miR159a-3p_csi-MIR159a.jpg]

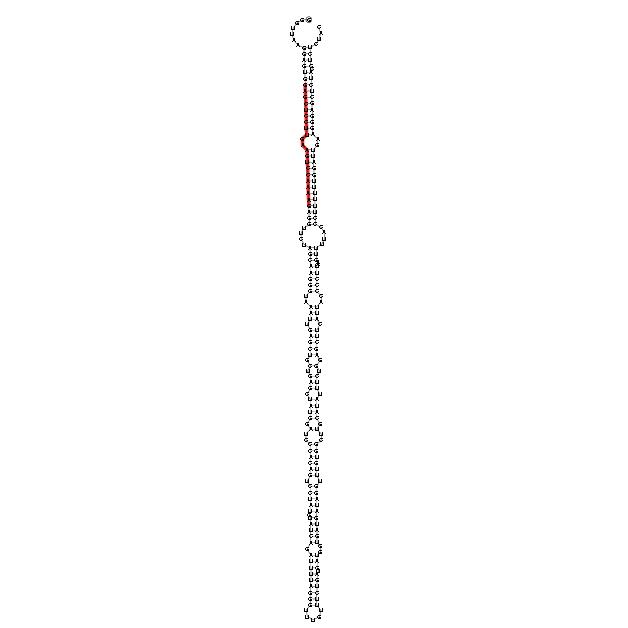

Supplement: Supplementary file 1 [file genes-13-01706-s001.zip › Figure S1. Known miRNAs Structure/csi-miR159a-5p_csi-MIR159a.jpg]

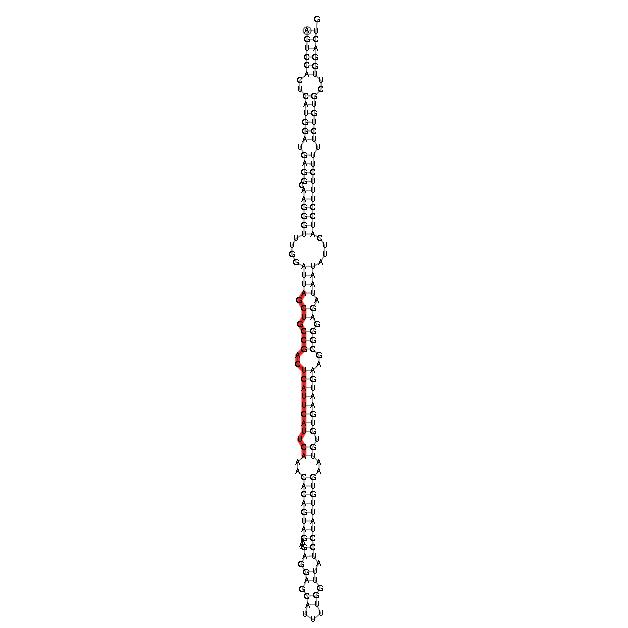

Supplement: Supplementary file 1 [file genes-13-01706-s001.zip › Figure S1. Known miRNAs Structure/csi-miR159b-5p_csi-MIR159b.jpg]

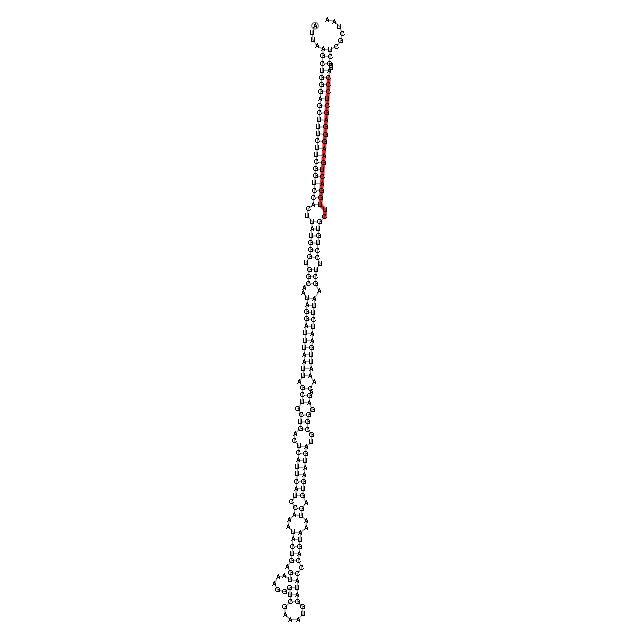

Supplement: Supplementary file 1 [file genes-13-01706-s001.zip › Figure S1. Known miRNAs Structure/csi-miR159c-3p_csi-MIR159c.jpg]

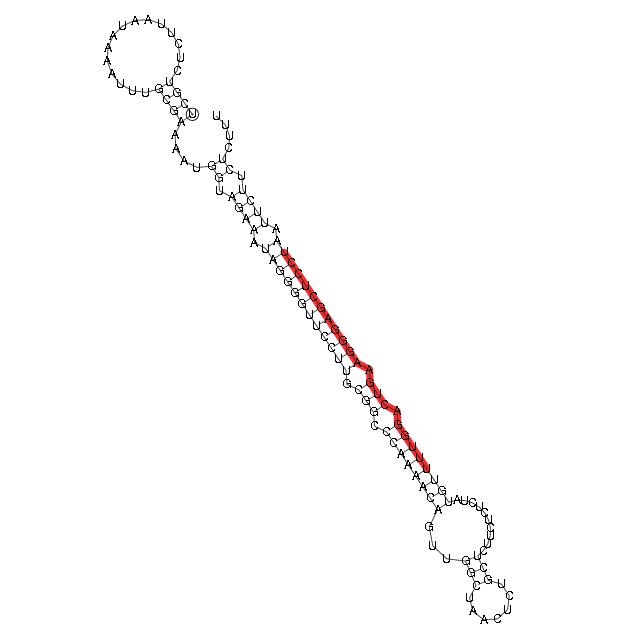

Supplement: Supplementary file 1 [file genes-13-01706-s001.zip › Figure S1. Known miRNAs Structure/csi-miR159d_csi-MIR159d.jpg]

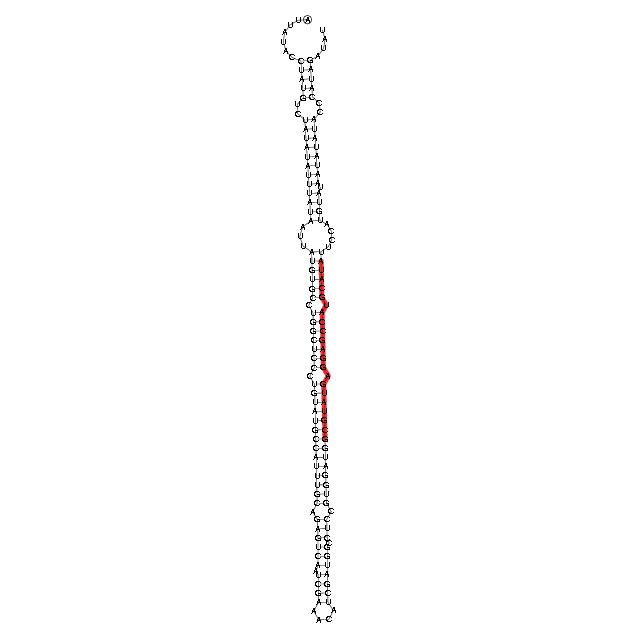

Supplement: Supplementary file 1 [file genes-13-01706-s001.zip › Figure S1. Known miRNAs Structure/csi-miR160a-3p_csi-MIR160a.jpg]

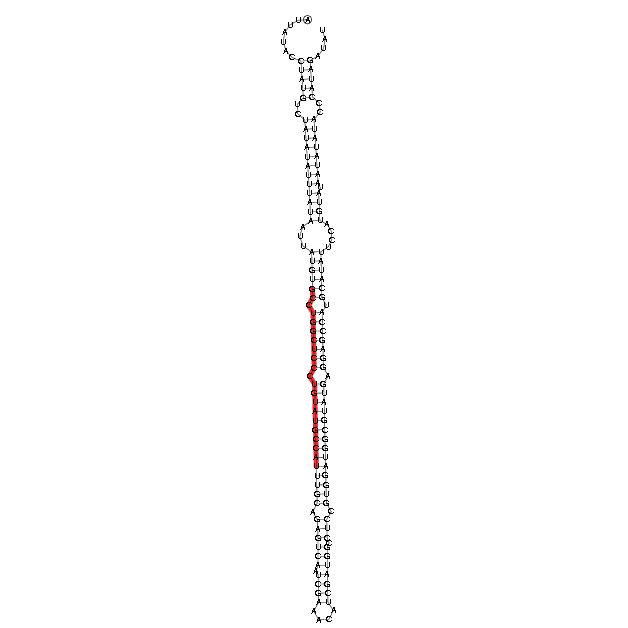

Supplement: Supplementary file 1 [file genes-13-01706-s001.zip › Figure S1. Known miRNAs Structure/csi-miR160a-5p_csi-MIR160a.jpg]

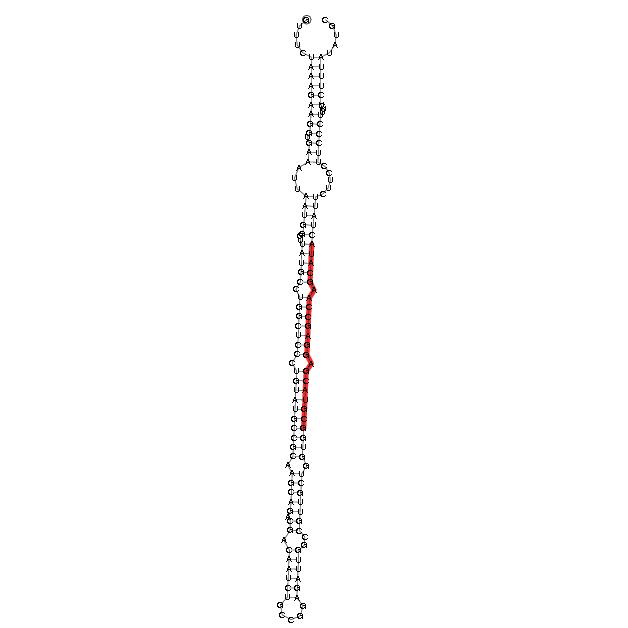

Supplement: Supplementary file 1 [file genes-13-01706-s001.zip › Figure S1. Known miRNAs Structure/csi-miR160b-3p_csi-MIR160b.jpg]

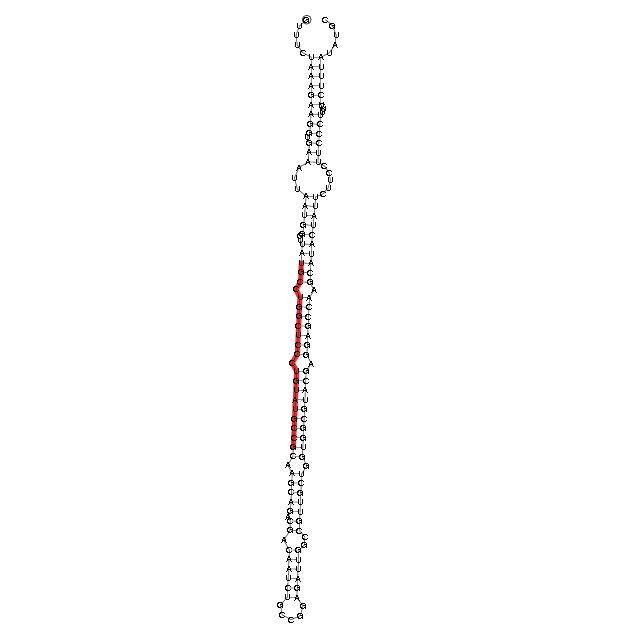

Supplement: Supplementary file 1 [file genes-13-01706-s001.zip › Figure S1. Known miRNAs Structure/csi-miR160b-5p_csi-MIR160b.jpg]

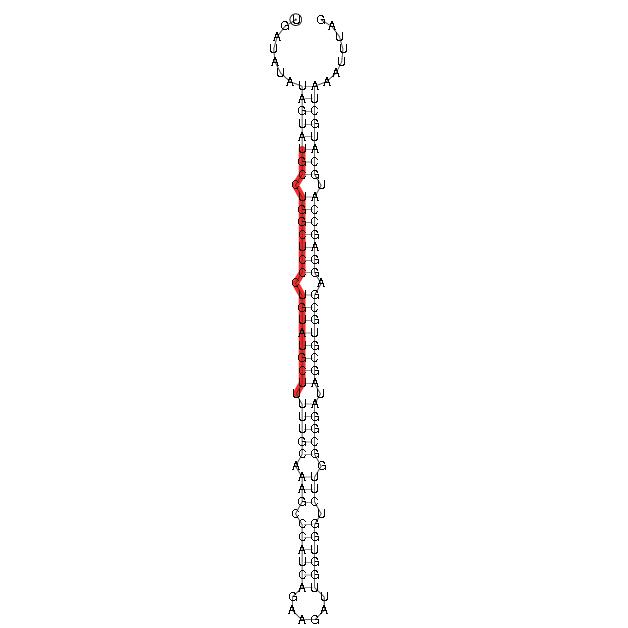

Supplement: Supplementary file 1 [file genes-13-01706-s001.zip › Figure S1. Known miRNAs Structure/csi-miR160c-5p_csi-MIR160c.jpg]

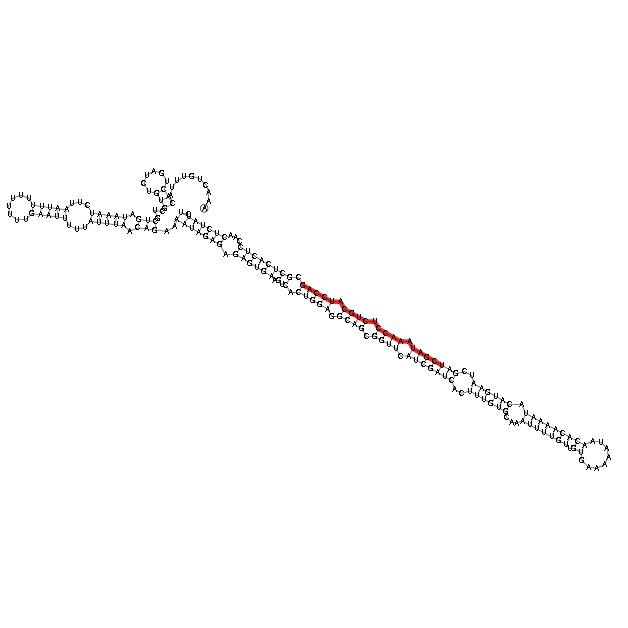

Supplement: Supplementary file 1 [file genes-13-01706-s001.zip › Figure S1. Known miRNAs Structure/csi-miR162-3p_csi-MIR162.jpg]

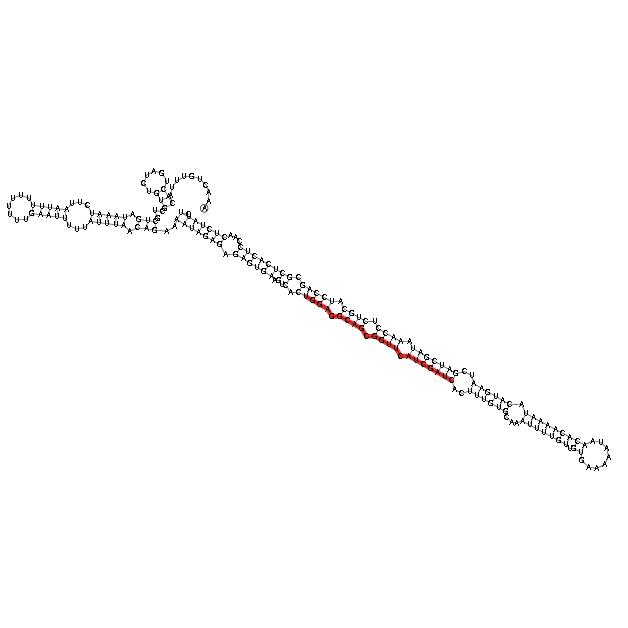

Supplement: Supplementary file 1 [file genes-13-01706-s001.zip › Figure S1. Known miRNAs Structure/csi-miR162-5p_csi-MIR162.jpg]

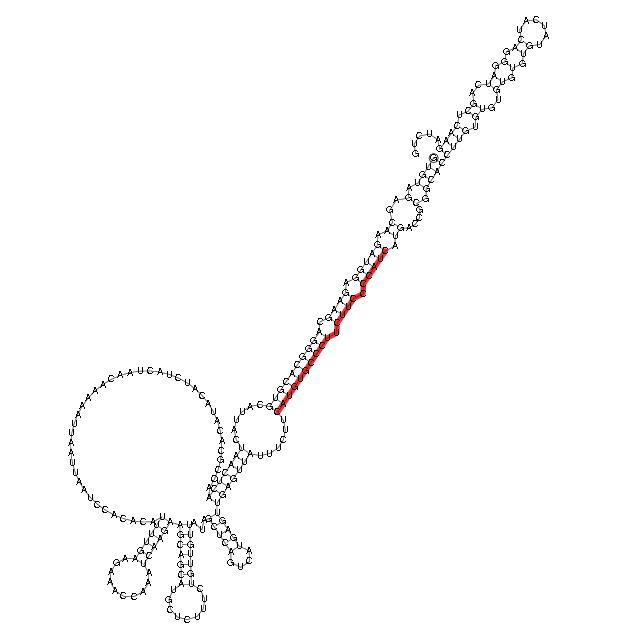

Supplement: Supplementary file 1 [file genes-13-01706-s001.zip › Figure S1. Known miRNAs Structure/csi-miR164a-3p_csi-MIR164a.jpg]

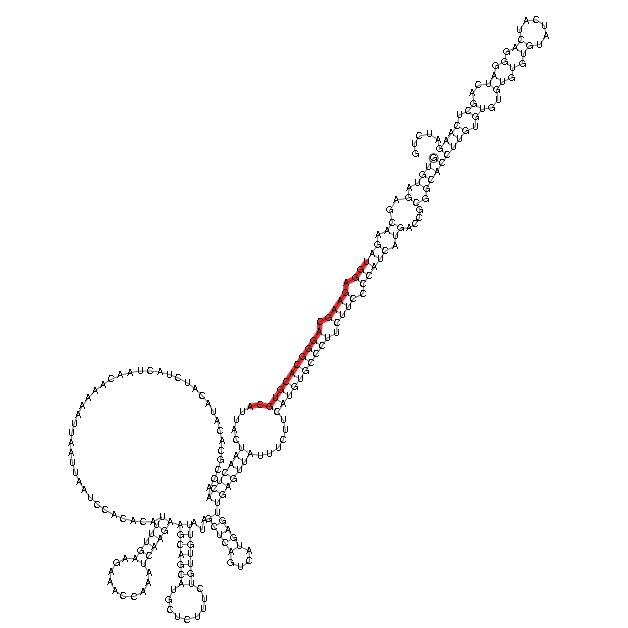

Supplement: Supplementary file 1 [file genes-13-01706-s001.zip › Figure S1. Known miRNAs Structure/csi-miR164a-5p_csi-MIR164a.jpg]

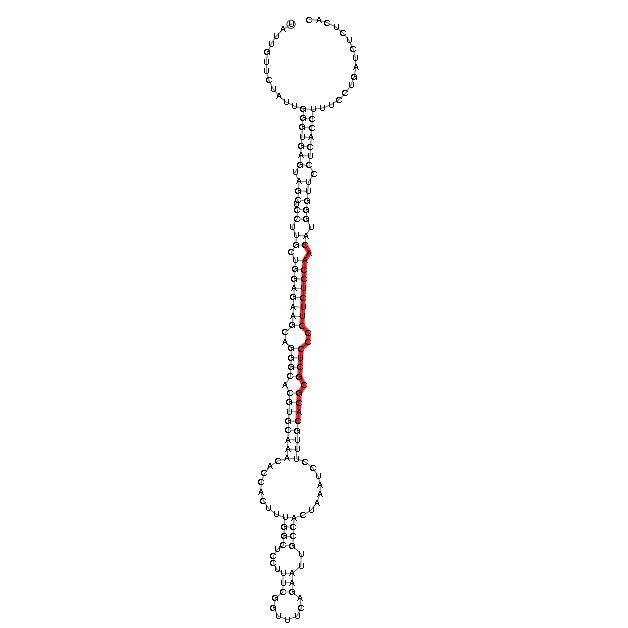

Supplement: Supplementary file 1 [file genes-13-01706-s001.zip › Figure S1. Known miRNAs Structure/csi-miR164b-3p_csi-MIR164b.jpg]

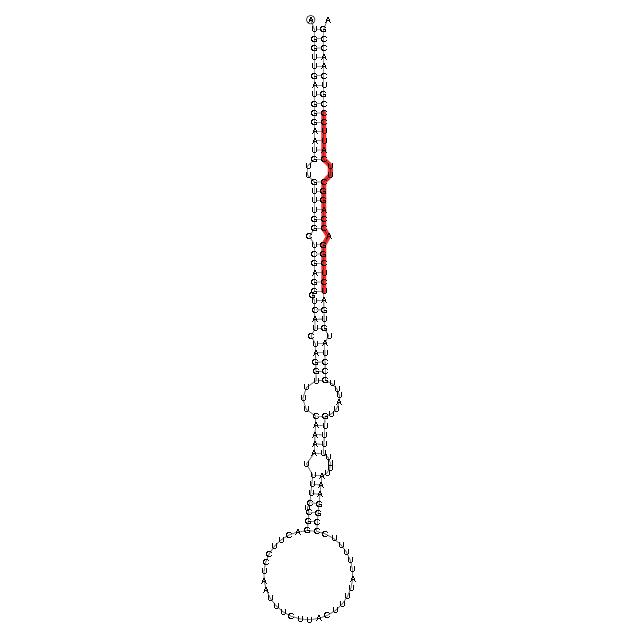

Supplement: Supplementary file 1 [file genes-13-01706-s001.zip › Figure S1. Known miRNAs Structure/csi-miR166b-3p_csi-MIR166b.jpg]

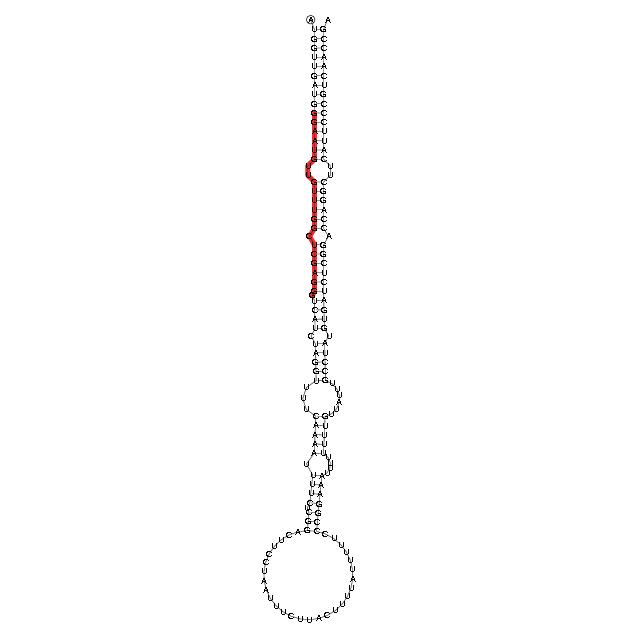

Supplement: Supplementary file 1 [file genes-13-01706-s001.zip › Figure S1. Known miRNAs Structure/csi-miR166b-5p_csi-MIR166b.jpg]

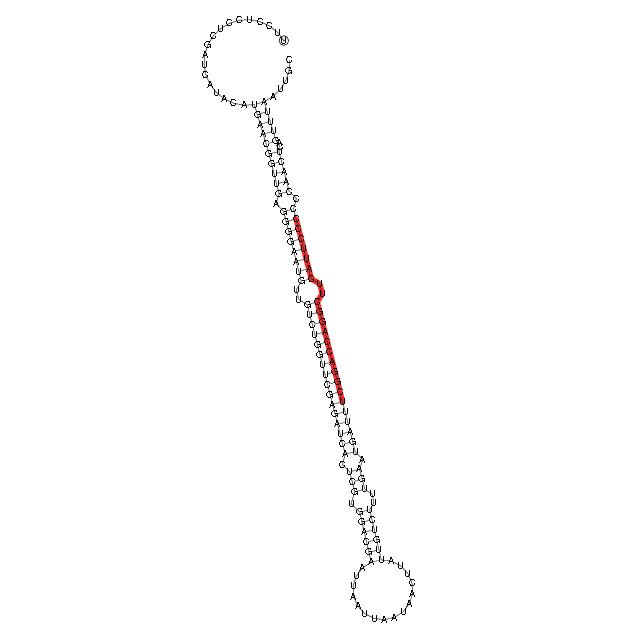

Supplement: Supplementary file 1 [file genes-13-01706-s001.zip › Figure S1. Known miRNAs Structure/csi-miR166c-3p_csi-MIR166c.jpg]

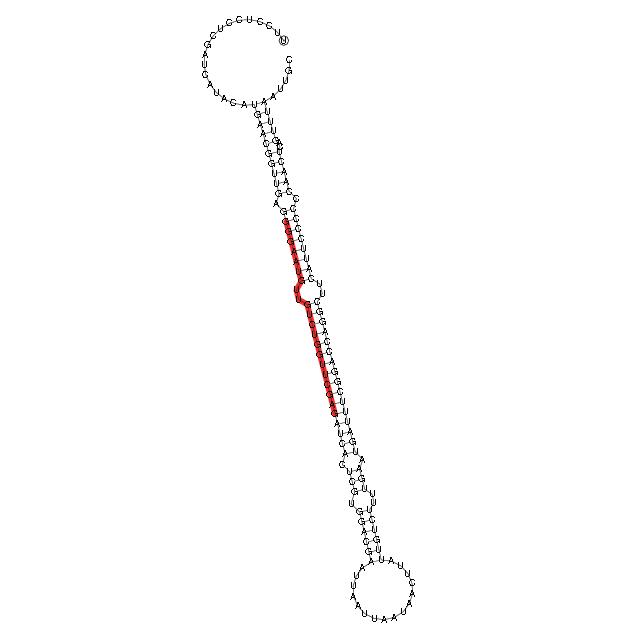

Supplement: Supplementary file 1 [file genes-13-01706-s001.zip › Figure S1. Known miRNAs Structure/csi-miR166c-5p_csi-MIR166c.jpg]

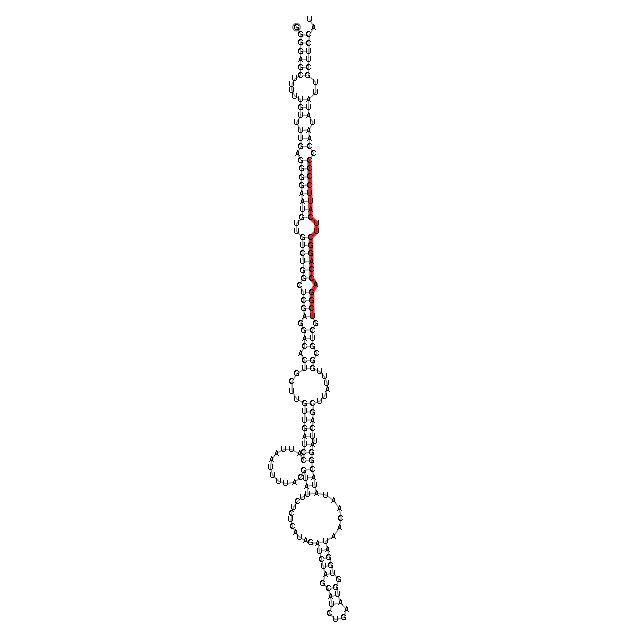

Supplement: Supplementary file 1 [file genes-13-01706-s001.zip › Figure S1. Known miRNAs Structure/csi-miR166e-3p_csi-MIR166e.jpg]

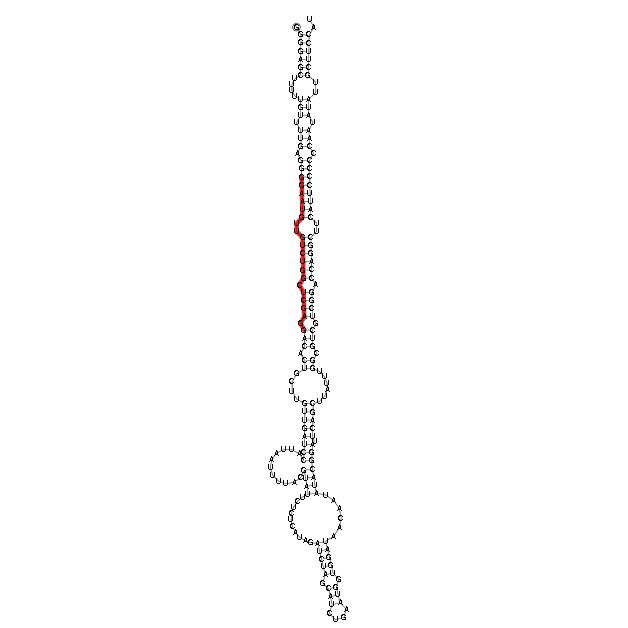

Supplement: Supplementary file 1 [file genes-13-01706-s001.zip › Figure S1. Known miRNAs Structure/csi-miR166e-5p_csi-MIR166e.jpg]

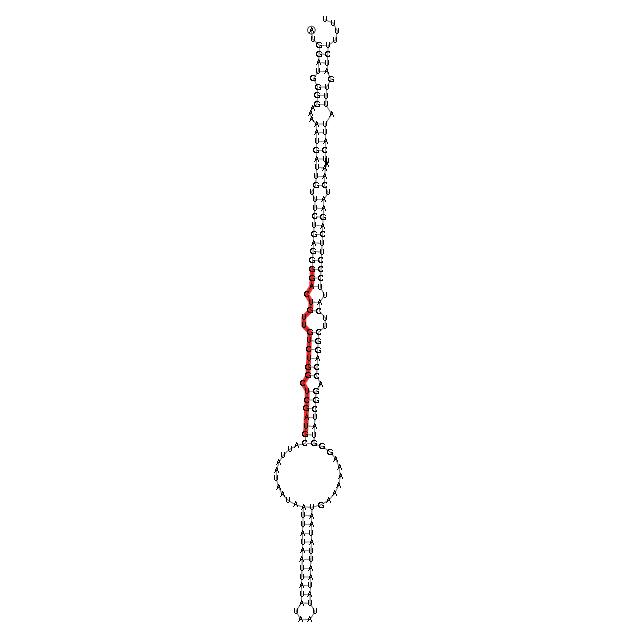

Supplement: Supplementary file 1 [file genes-13-01706-s001.zip › Figure S1. Known miRNAs Structure/csi-miR166f-5p_csi-MIR166f.jpg]

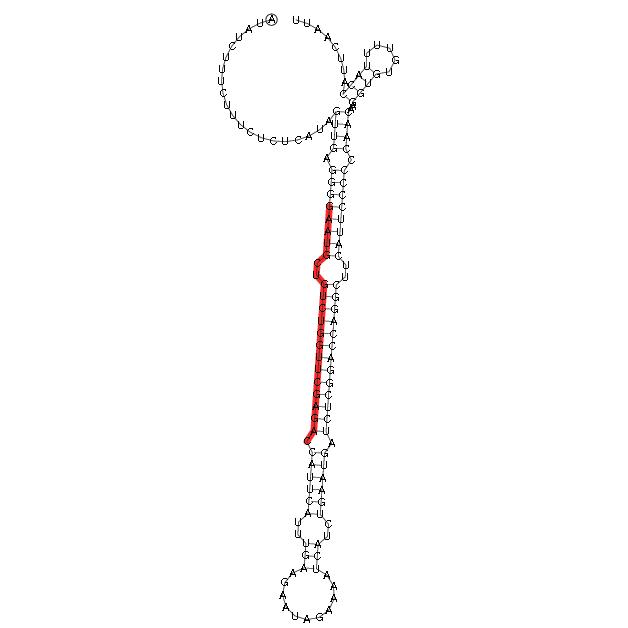

Supplement: Supplementary file 1 [file genes-13-01706-s001.zip › Figure S1. Known miRNAs Structure/csi-miR166g-5p_csi-MIR166g.jpg]

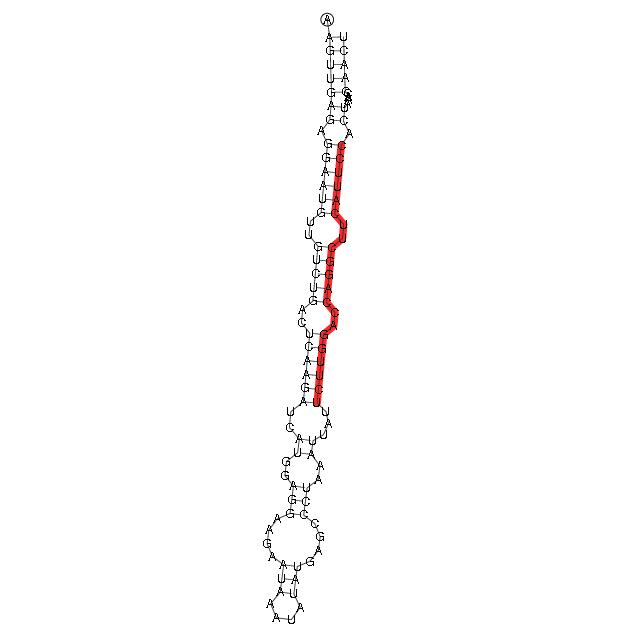

Supplement: Supplementary file 1 [file genes-13-01706-s001.zip › Figure S1. Known miRNAs Structure/csi-miR166i-3p_csi-MIR166i.jpg]

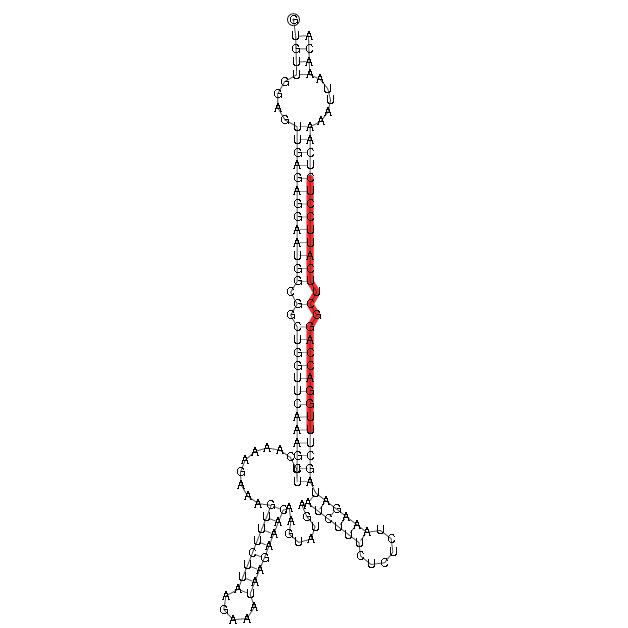

Supplement: Supplementary file 1 [file genes-13-01706-s001.zip › Figure S1. Known miRNAs Structure/csi-miR166j-3p_csi-MIR166j.jpg]

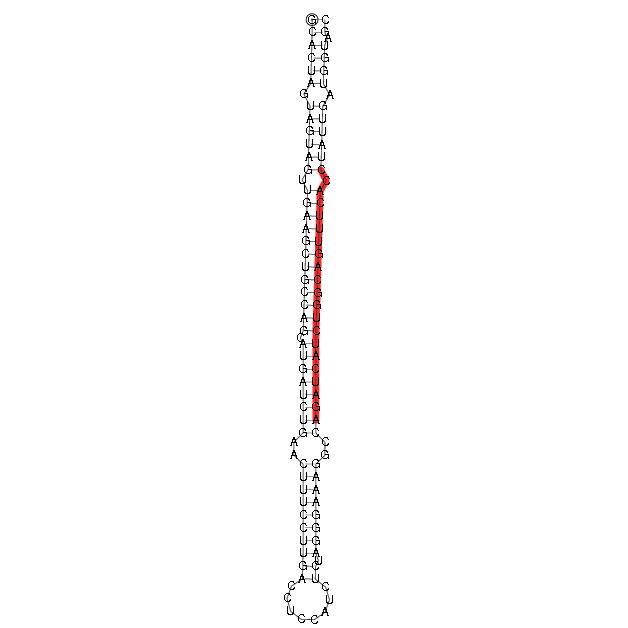

Supplement: Supplementary file 1 [file genes-13-01706-s001.zip › Figure S1. Known miRNAs Structure/csi-miR167a-3p_csi-MIR167a.jpg]

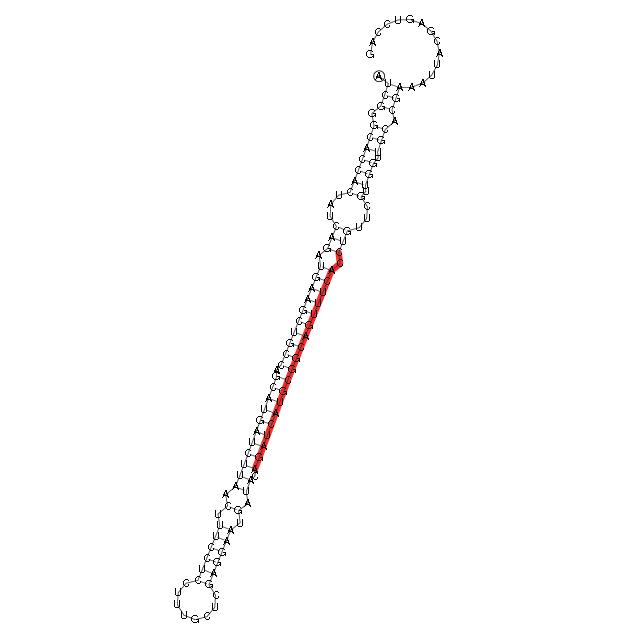

Supplement: Supplementary file 1 [file genes-13-01706-s001.zip › Figure S1. Known miRNAs Structure/csi-miR167b-3p_csi-MIR167b.jpg]

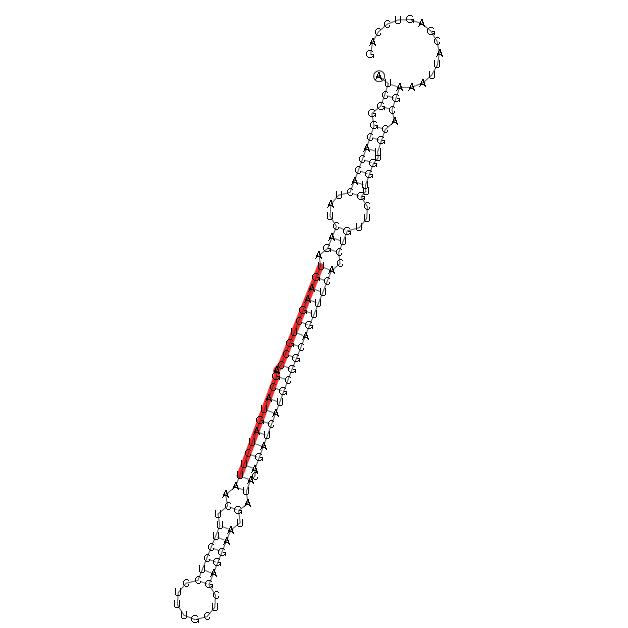

Supplement: Supplementary file 1 [file genes-13-01706-s001.zip › Figure S1. Known miRNAs Structure/csi-miR167b-5p_csi-MIR167b.jpg]

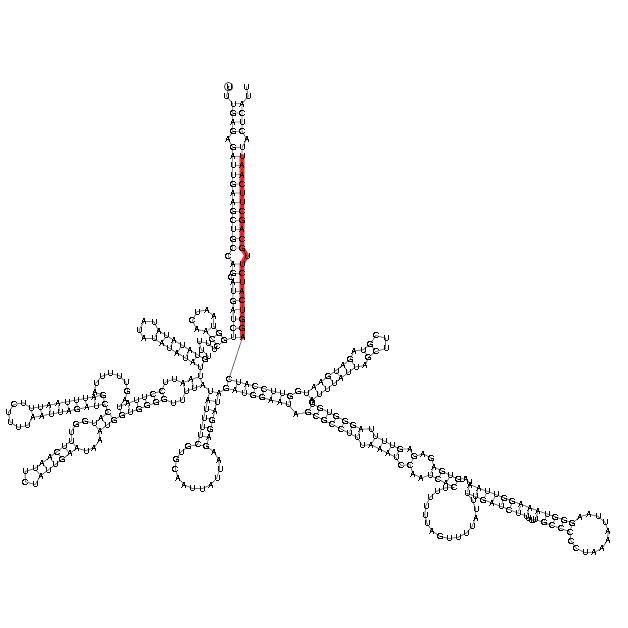

Supplement: Supplementary file 1 [file genes-13-01706-s001.zip › Figure S1. Known miRNAs Structure/csi-miR167c-3p_csi-MIR167c.jpg]

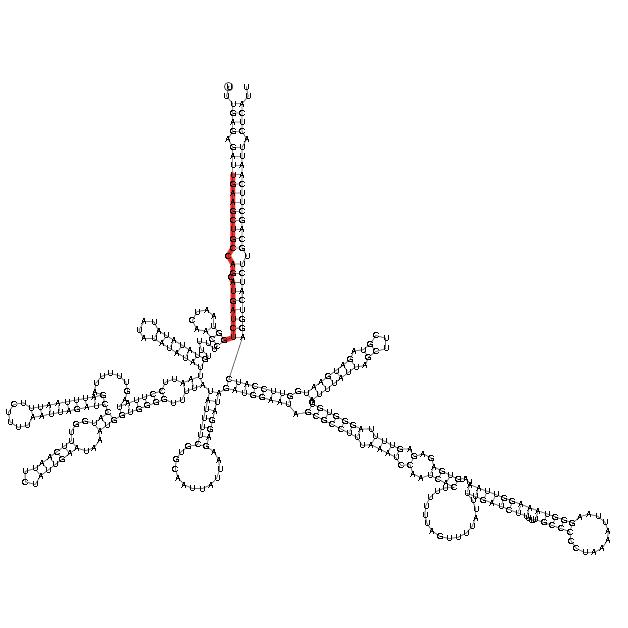

Supplement: Supplementary file 1 [file genes-13-01706-s001.zip › Figure S1. Known miRNAs Structure/csi-miR167c-5p_csi-MIR167c.jpg]

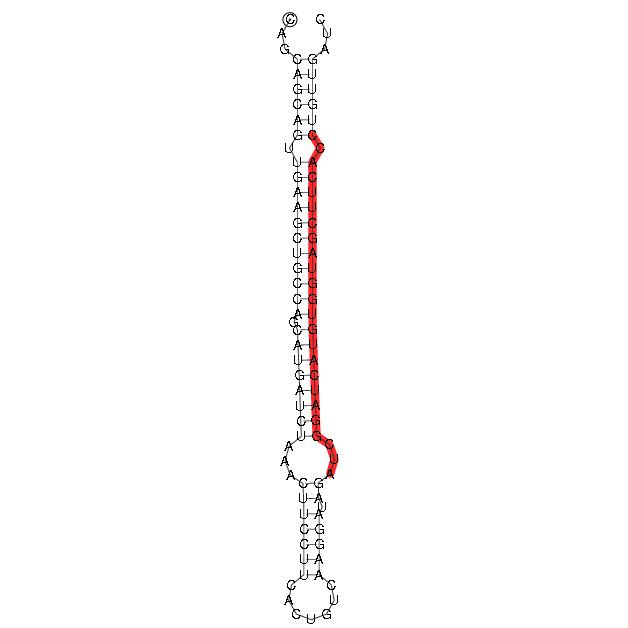

Supplement: Supplementary file 1 [file genes-13-01706-s001.zip › Figure S1. Known miRNAs Structure/csi-miR167d-3p_csi-MIR167d.jpg]

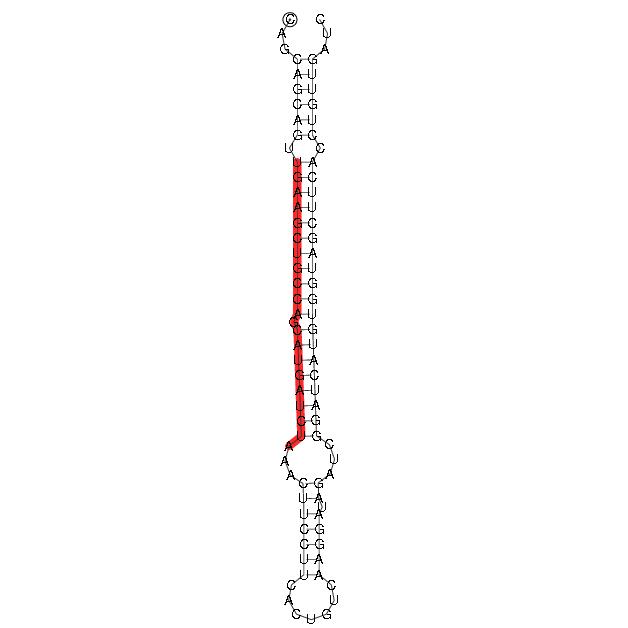

Supplement: Supplementary file 1 [file genes-13-01706-s001.zip › Figure S1. Known miRNAs Structure/csi-miR167d-5p_csi-MIR167d.jpg]

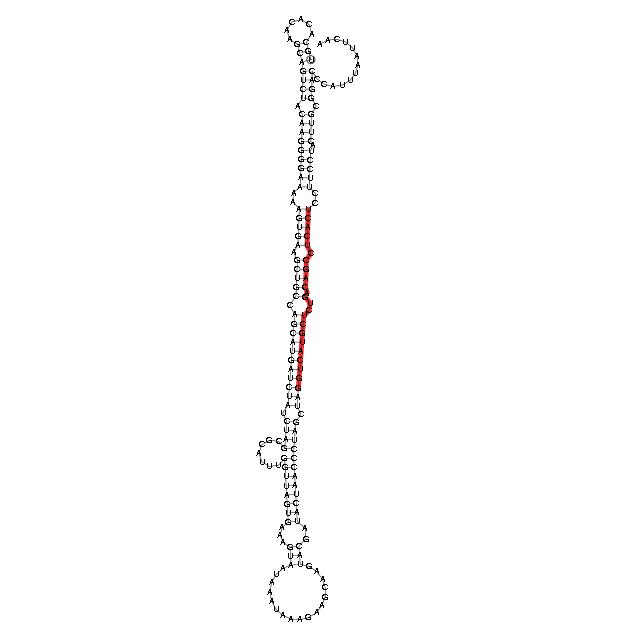

Supplement: Supplementary file 1 [file genes-13-01706-s001.zip › Figure S1. Known miRNAs Structure/csi-miR167e-3p_csi-MIR167e.jpg]

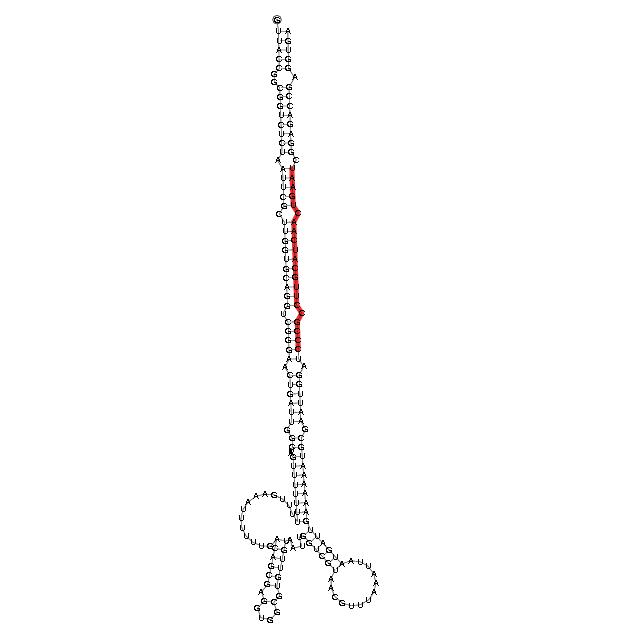

Supplement: Supplementary file 1 [file genes-13-01706-s001.zip › Figure S1. Known miRNAs Structure/csi-miR168-3p_csi-MIR168.jpg]

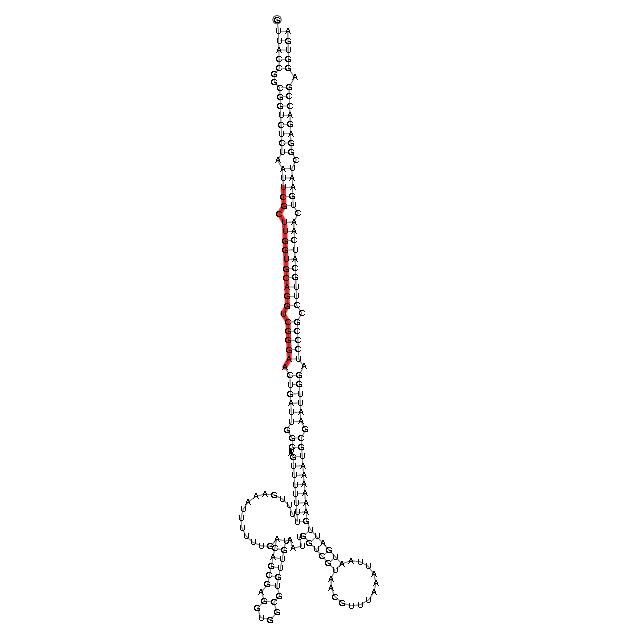

Supplement: Supplementary file 1 [file genes-13-01706-s001.zip › Figure S1. Known miRNAs Structure/csi-miR168-5p_csi-MIR168.jpg]

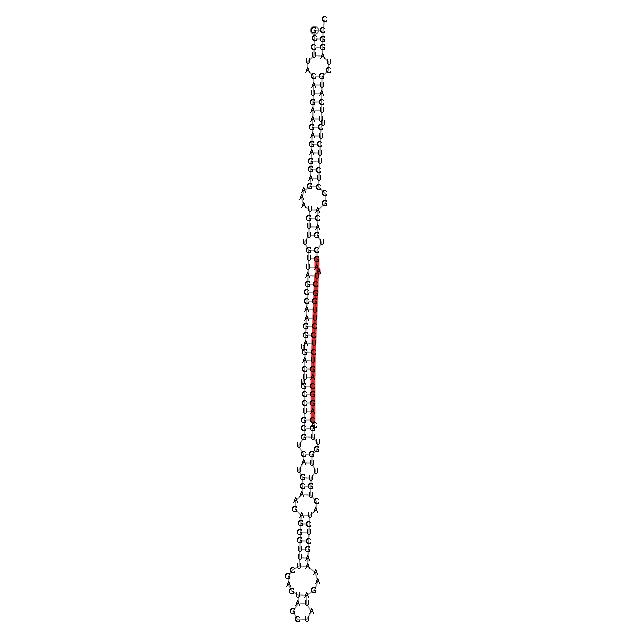

Supplement: Supplementary file 1 [file genes-13-01706-s001.zip › Figure S1. Known miRNAs Structure/csi-miR169c-3p_csi-MIR169c.jpg]

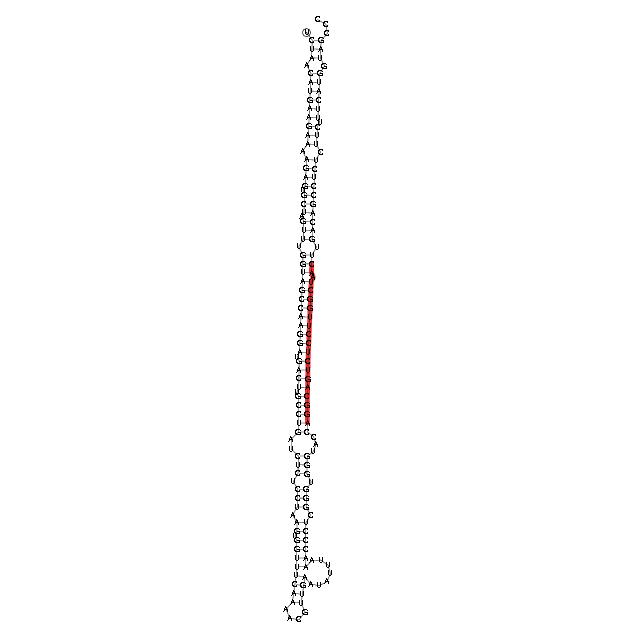

Supplement: Supplementary file 1 [file genes-13-01706-s001.zip › Figure S1. Known miRNAs Structure/csi-miR169l-3p_csi-MIR169l.jpg]

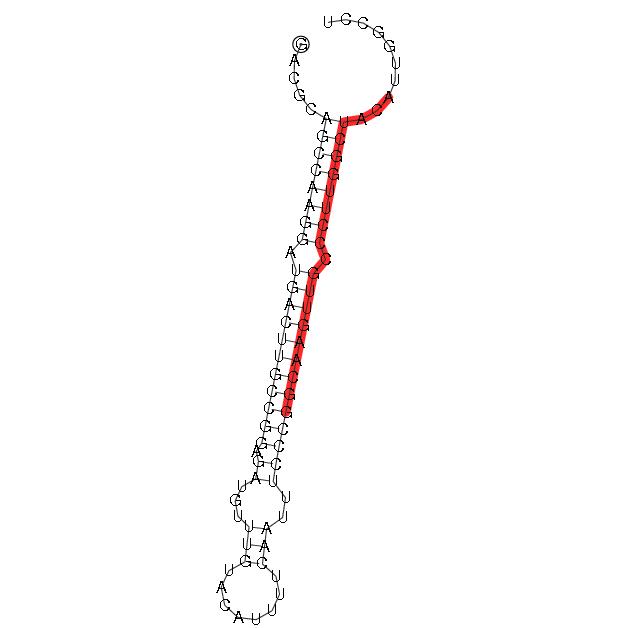

Supplement: Supplementary file 1 [file genes-13-01706-s001.zip › Figure S1. Known miRNAs Structure/csi-miR169n-3p_csi-MIR169n.jpg]

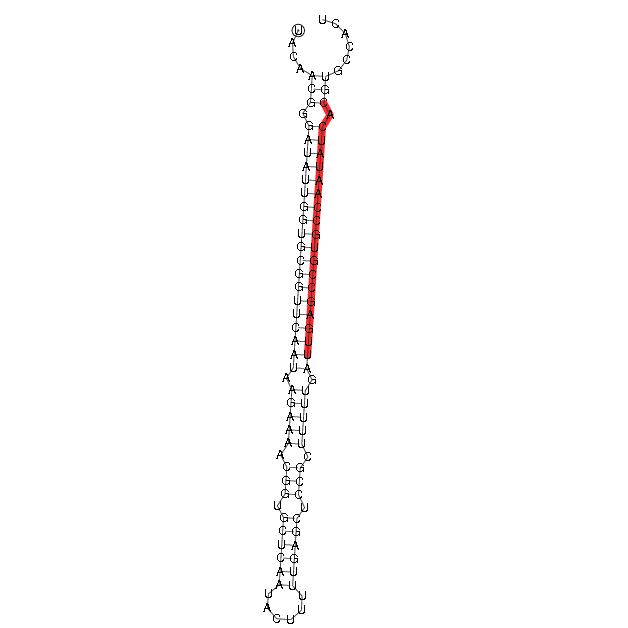

Supplement: Supplementary file 1 [file genes-13-01706-s001.zip › Figure S1. Known miRNAs Structure/csi-miR171a_csi-MIR171a.jpg]

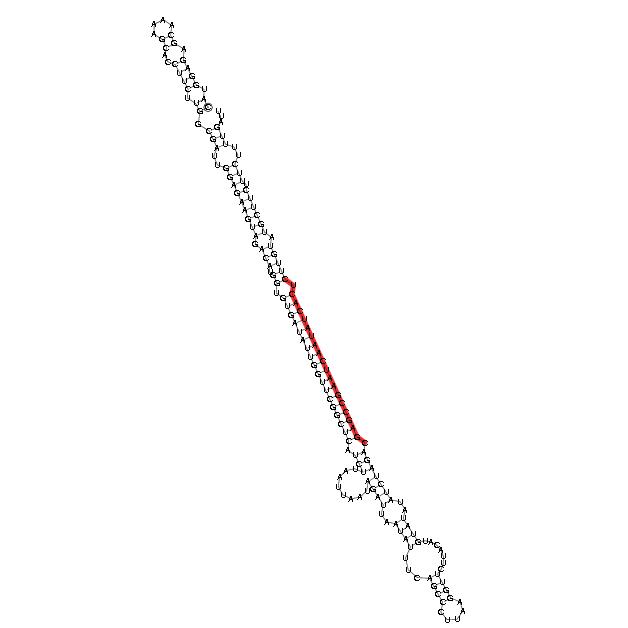

Supplement: Supplementary file 1 [file genes-13-01706-s001.zip › Figure S1. Known miRNAs Structure/csi-miR171b-3p_csi-MIR171b.jpg]

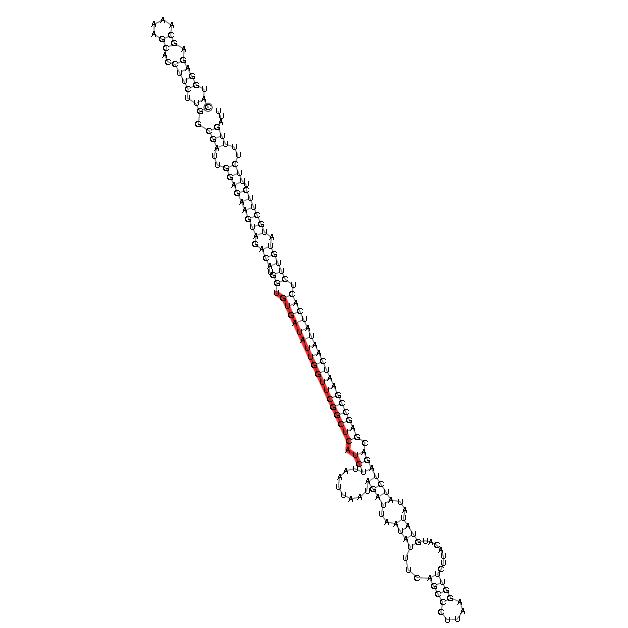

Supplement: Supplementary file 1 [file genes-13-01706-s001.zip › Figure S1. Known miRNAs Structure/csi-miR171b-5p_csi-MIR171b.jpg]

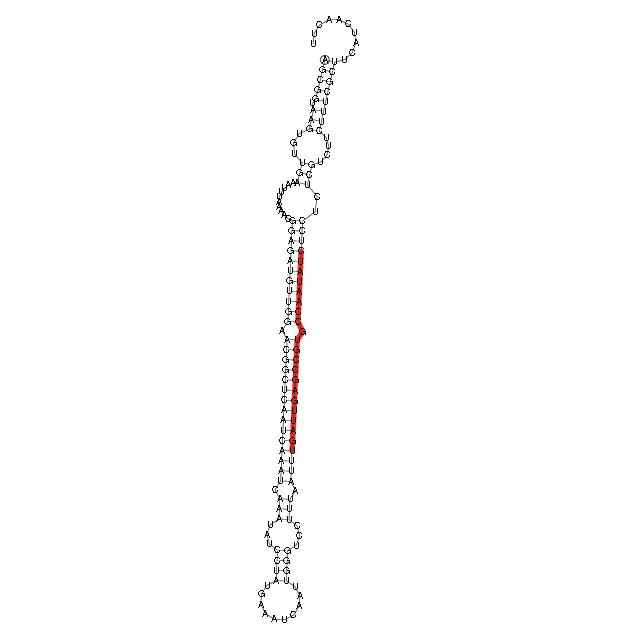

Supplement: Supplementary file 1 [file genes-13-01706-s001.zip › Figure S1. Known miRNAs Structure/csi-miR171c-3p_csi-MIR171c.jpg]

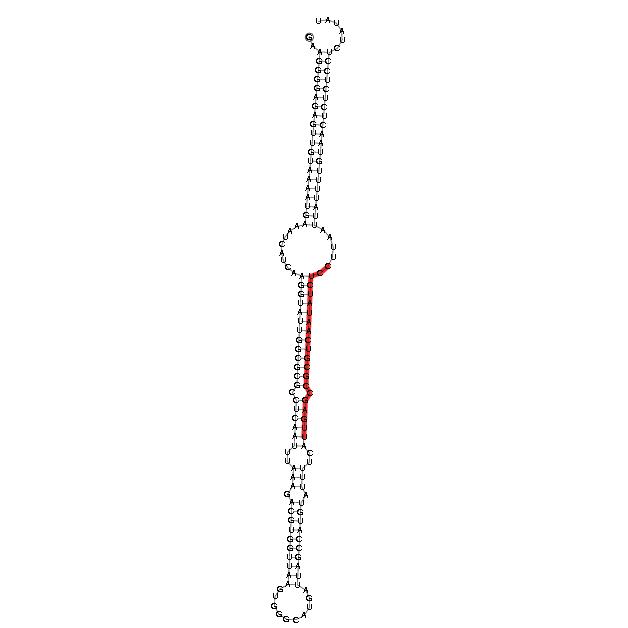

Supplement: Supplementary file 1 [file genes-13-01706-s001.zip › Figure S1. Known miRNAs Structure/csi-miR171e-3p_csi-MIR171e.jpg]

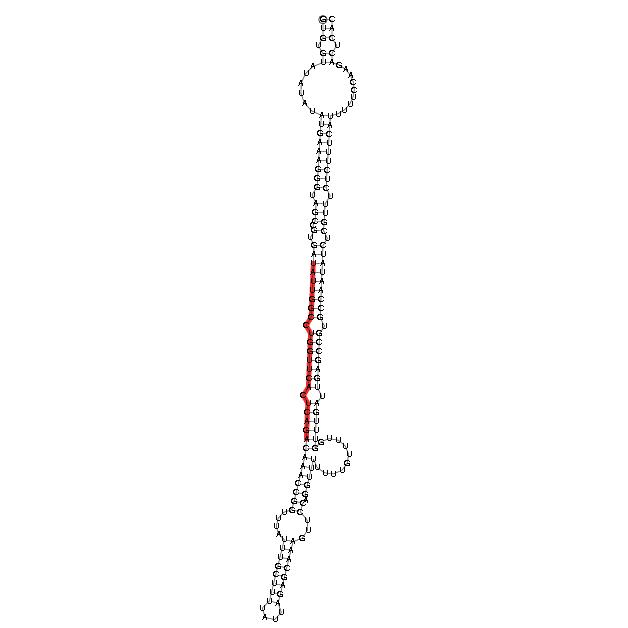

Supplement: Supplementary file 1 [file genes-13-01706-s001.zip › Figure S1. Known miRNAs Structure/csi-miR171f-5p_csi-MIR171f.jpg]

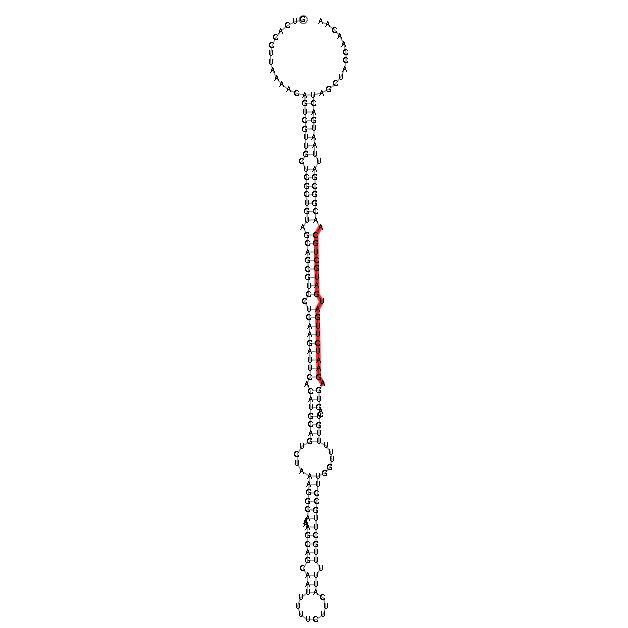

Supplement: Supplementary file 1 [file genes-13-01706-s001.zip › Figure S1. Known miRNAs Structure/csi-miR172a-3p_csi-MIR172a.jpg]

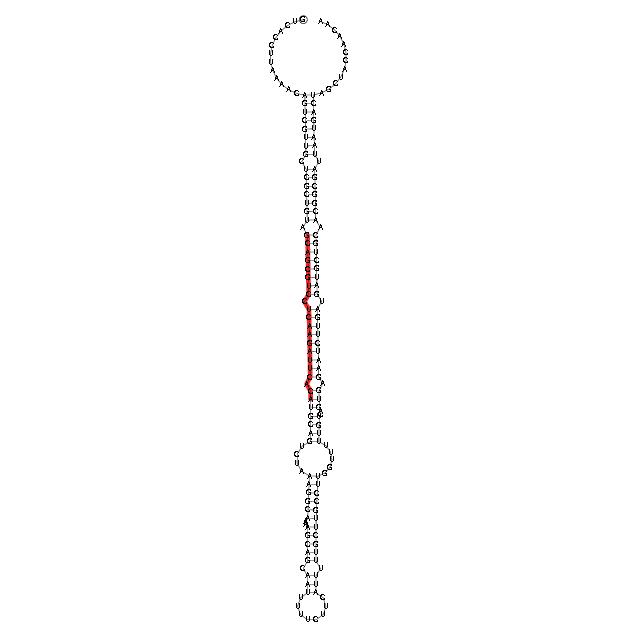

Supplement: Supplementary file 1 [file genes-13-01706-s001.zip › Figure S1. Known miRNAs Structure/csi-miR172a-5p_csi-MIR172a.jpg]

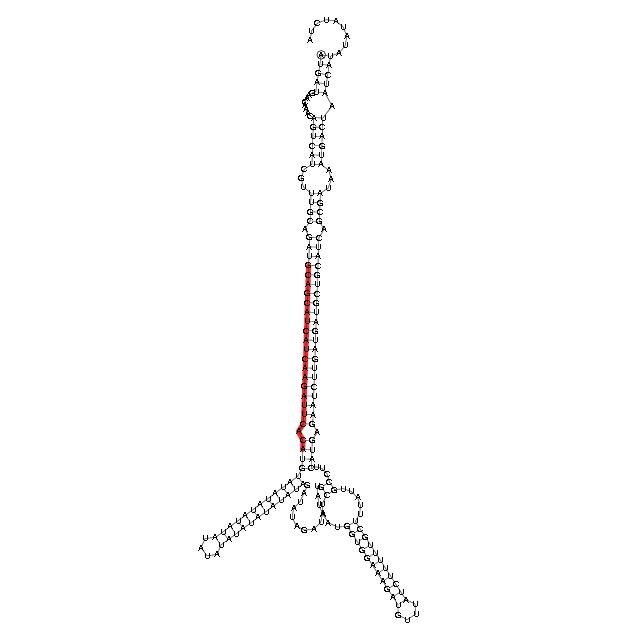

Supplement: Supplementary file 1 [file genes-13-01706-s001.zip › Figure S1. Known miRNAs Structure/csi-miR172b-5p_csi-MIR172b.jpg]

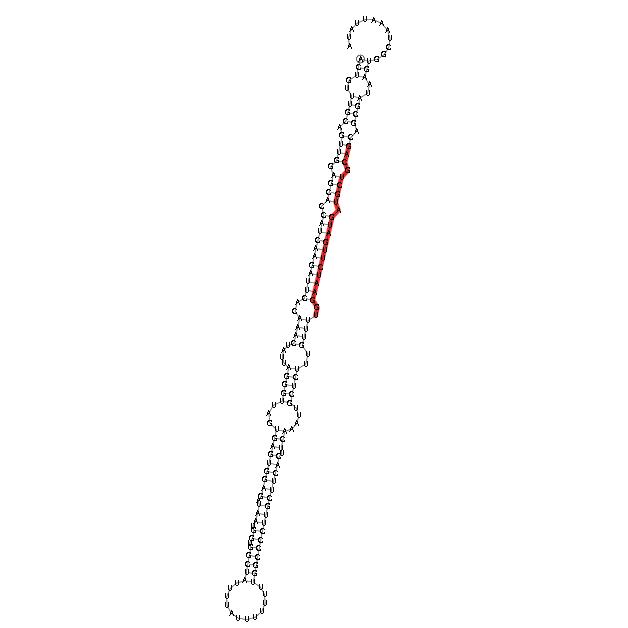

Supplement: Supplementary file 1 [file genes-13-01706-s001.zip › Figure S1. Known miRNAs Structure/csi-miR172c-3p_csi-MIR172c.jpg]

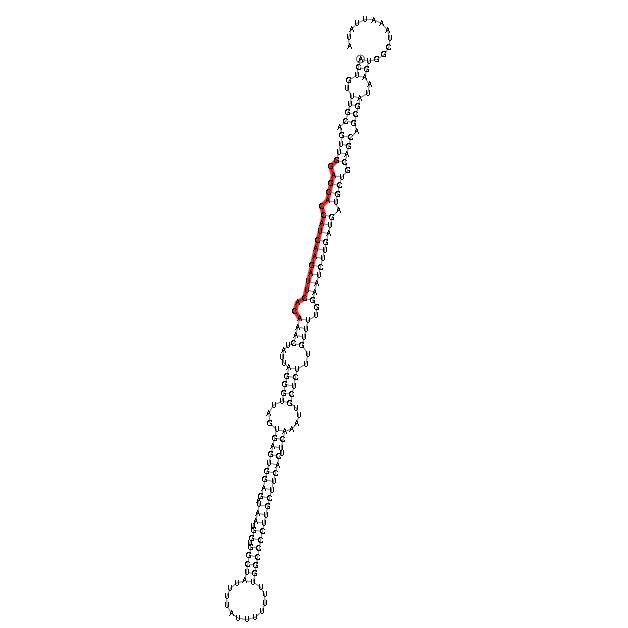

Supplement: Supplementary file 1 [file genes-13-01706-s001.zip › Figure S1. Known miRNAs Structure/csi-miR172c-5p_csi-MIR172c.jpg]

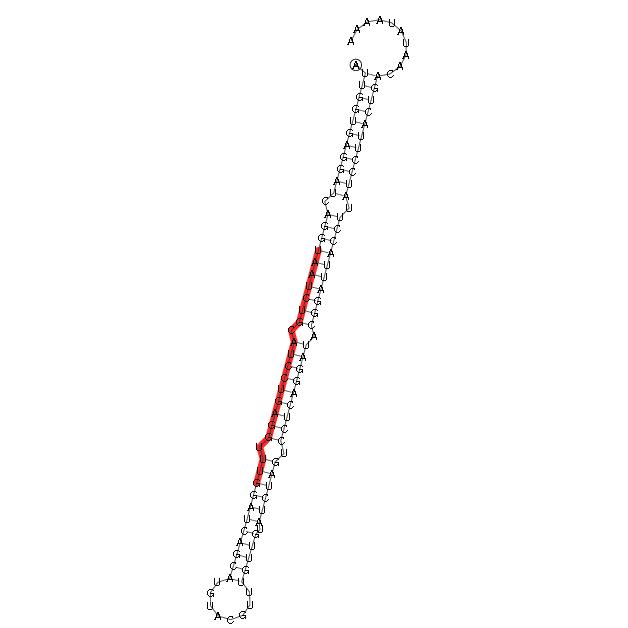

Supplement: Supplementary file 1 [file genes-13-01706-s001.zip › Figure S1. Known miRNAs Structure/csi-miR2111-5p_csi-MIR2111.jpg]

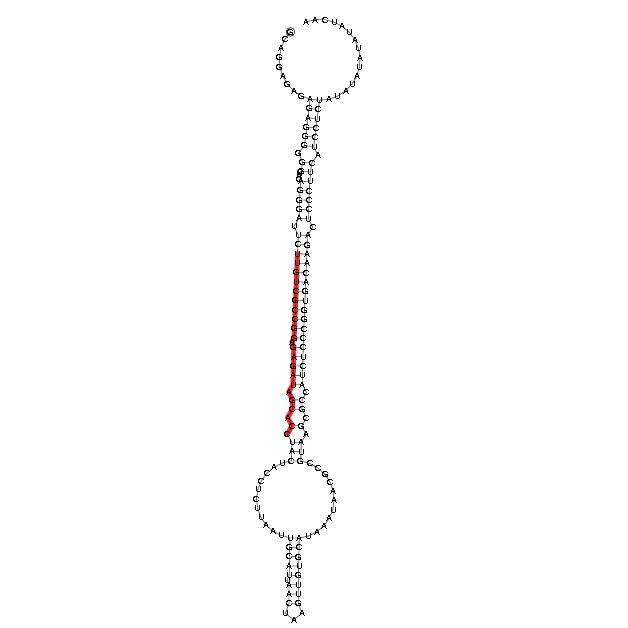

Supplement: Supplementary file 1 [file genes-13-01706-s001.zip › Figure S1. Known miRNAs Structure/csi-miR3627a-5p_csi-MIR3627a.jpg]

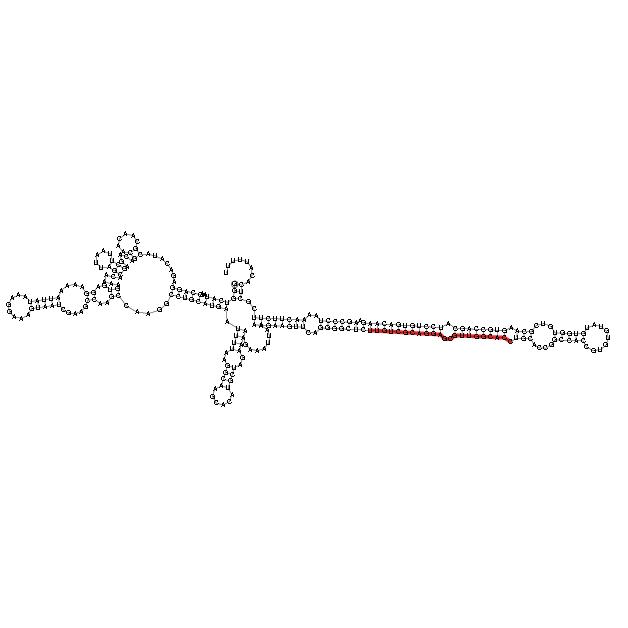

Supplement: Supplementary file 1 [file genes-13-01706-s001.zip › Figure S1. Known miRNAs Structure/csi-miR3627b-3p_csi-MIR3627b.jpg]

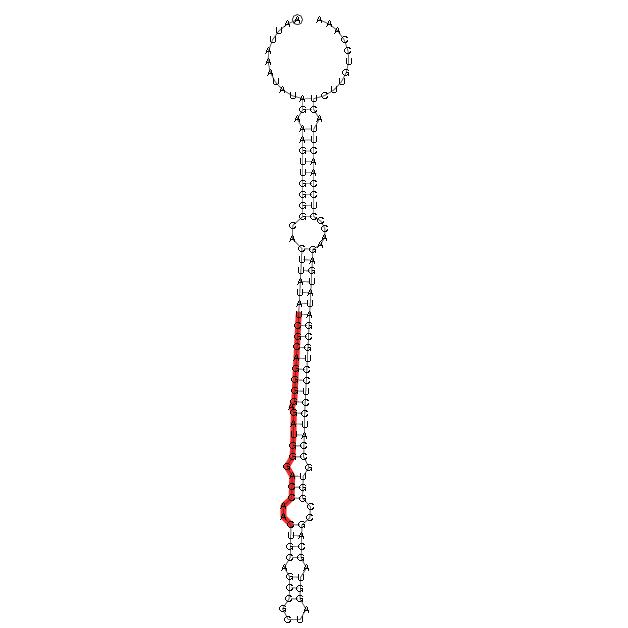

Supplement: Supplementary file 1 [file genes-13-01706-s001.zip › Figure S1. Known miRNAs Structure/csi-miR3627c-5p_csi-MIR3627c.jpg]

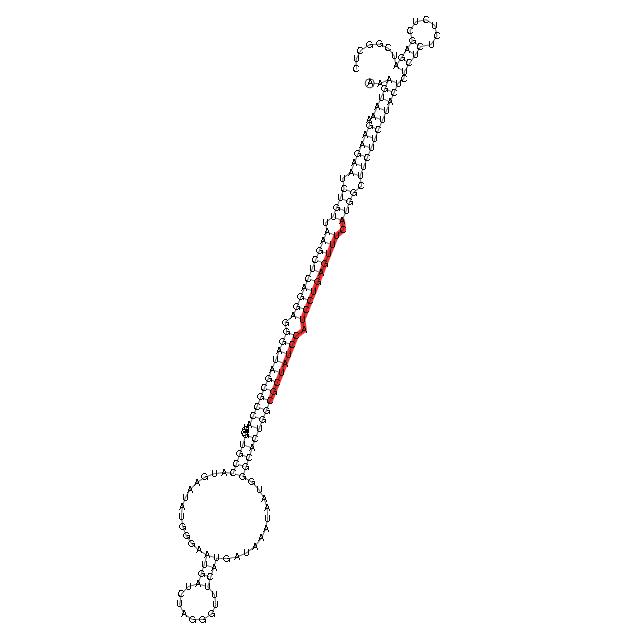

Supplement: Supplementary file 1 [file genes-13-01706-s001.zip › Figure S1. Known miRNAs Structure/csi-miR390a-3p_csi-MIR390a.jpg]

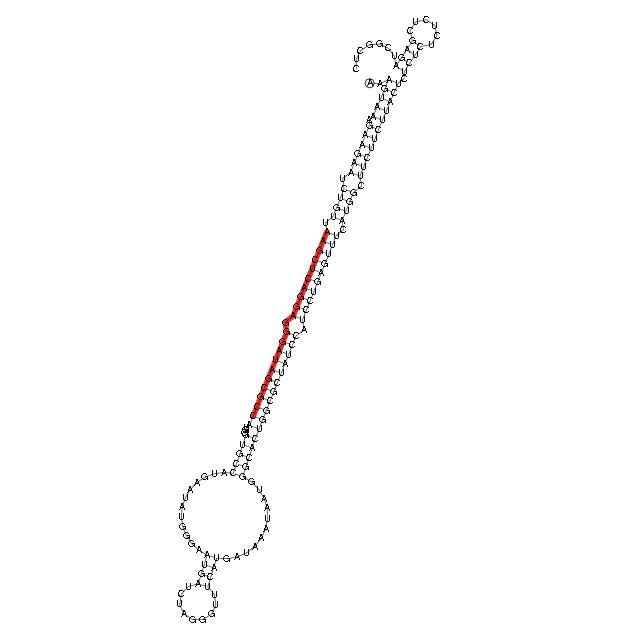

Supplement: Supplementary file 1 [file genes-13-01706-s001.zip › Figure S1. Known miRNAs Structure/csi-miR390a-5p_csi-MIR390a.jpg]

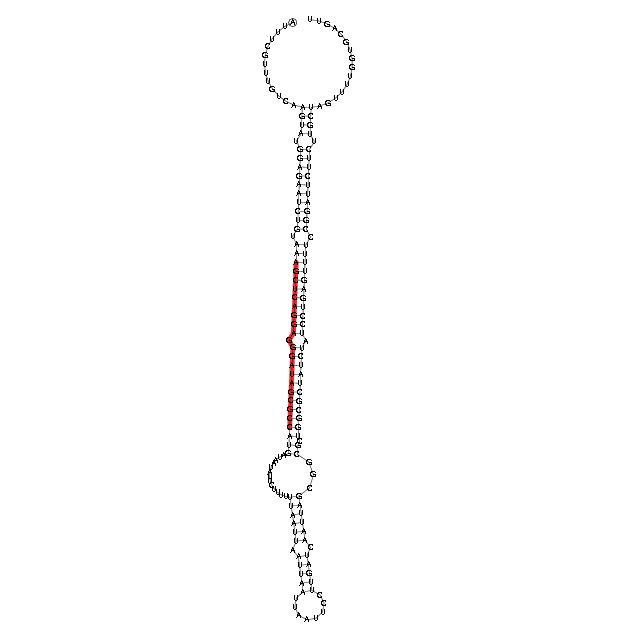

Supplement: Supplementary file 1 [file genes-13-01706-s001.zip › Figure S1. Known miRNAs Structure/csi-miR390b-5p_csi-MIR390b.jpg]

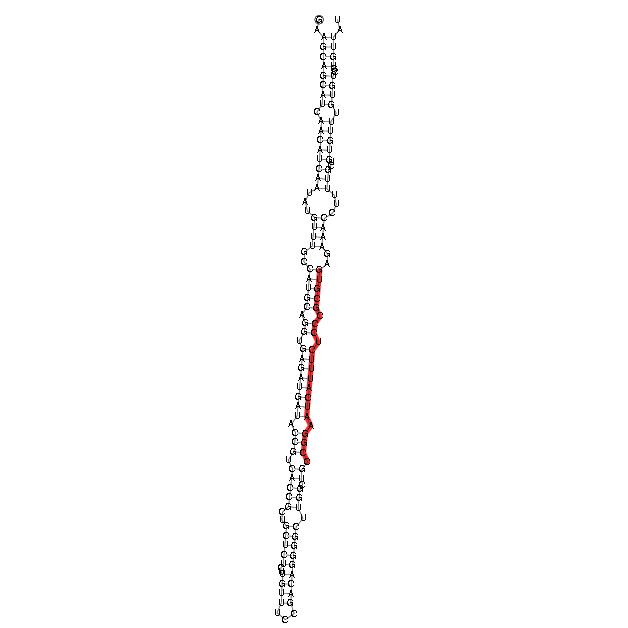

Supplement: Supplementary file 1 [file genes-13-01706-s001.zip › Figure S1. Known miRNAs Structure/csi-miR391-3p_csi-MIR391.jpg]

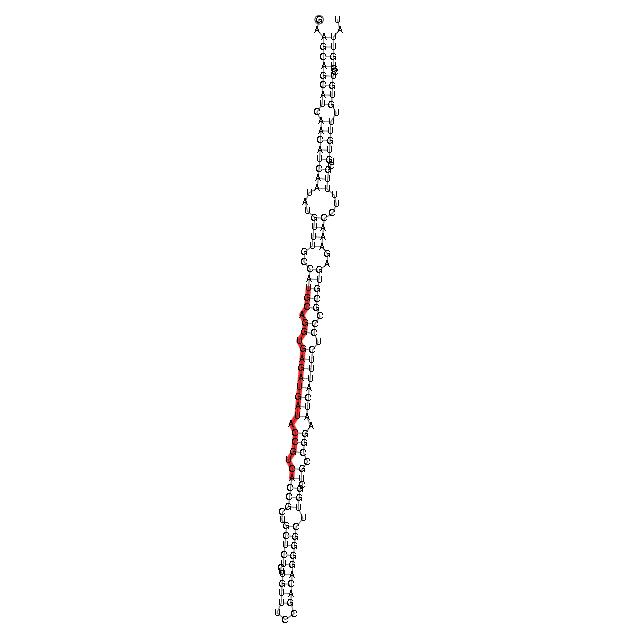

Supplement: Supplementary file 1 [file genes-13-01706-s001.zip › Figure S1. Known miRNAs Structure/csi-miR391-5p_csi-MIR391.jpg]

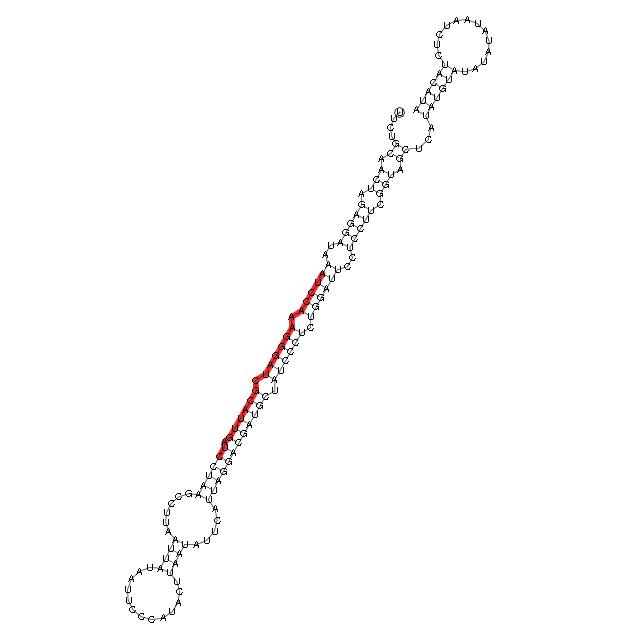

Supplement: Supplementary file 1 [file genes-13-01706-s001.zip › Figure S1. Known miRNAs Structure/csi-miR393a_csi-MIR393a.jpg]

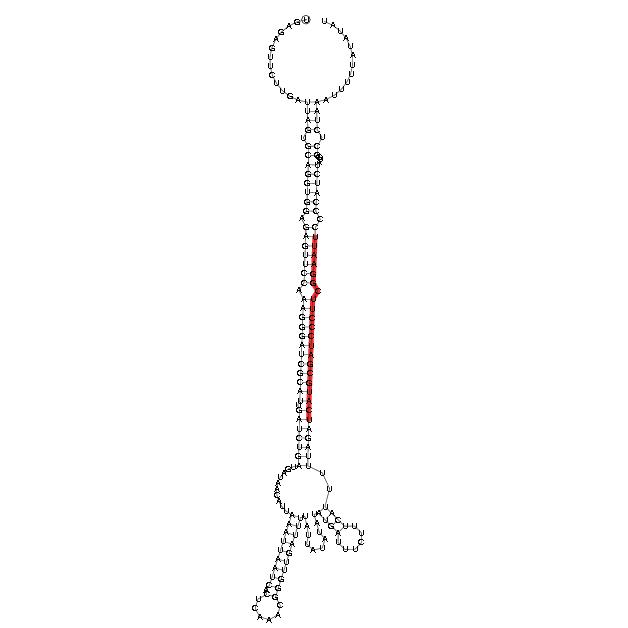

Supplement: Supplementary file 1 [file genes-13-01706-s001.zip › Figure S1. Known miRNAs Structure/csi-miR393b-3p_csi-MIR393b.jpg]

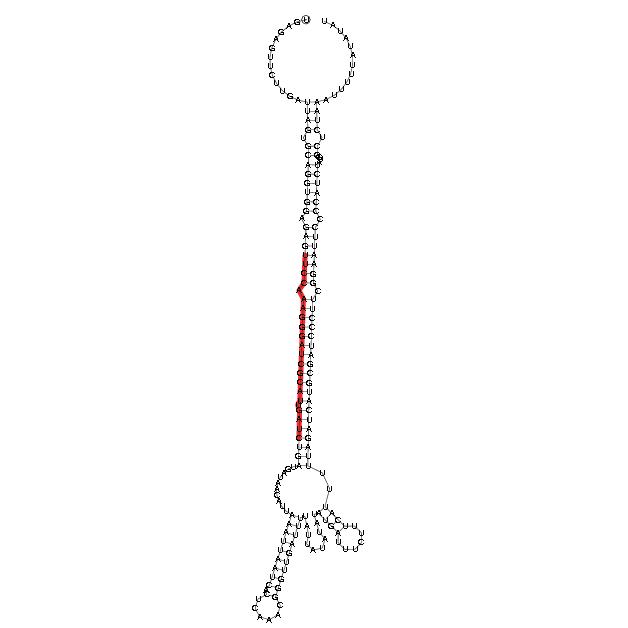

Supplement: Supplementary file 1 [file genes-13-01706-s001.zip › Figure S1. Known miRNAs Structure/csi-miR393b-5p_csi-MIR393b.jpg]

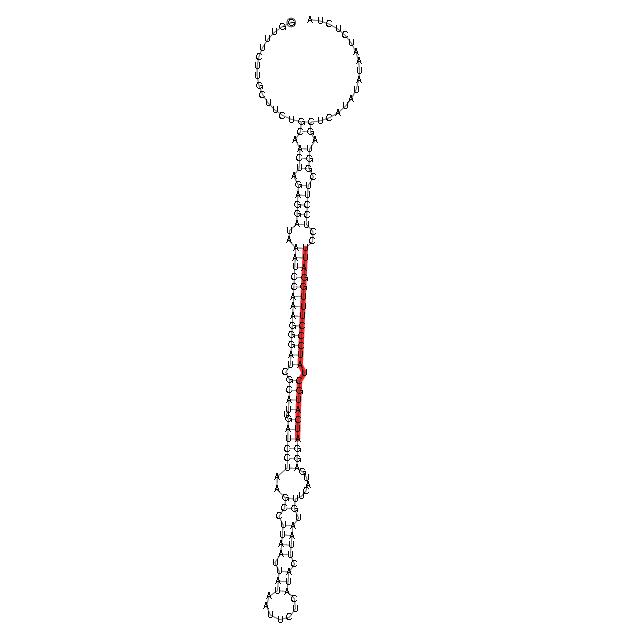

Supplement: Supplementary file 1 [file genes-13-01706-s001.zip › Figure S1. Known miRNAs Structure/csi-miR393c-3p_csi-MIR393c.jpg]

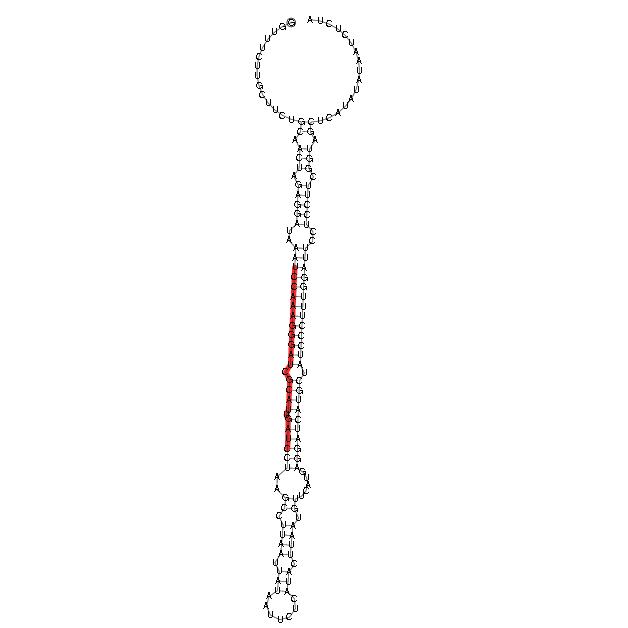

Supplement: Supplementary file 1 [file genes-13-01706-s001.zip › Figure S1. Known miRNAs Structure/csi-miR393c-5p_csi-MIR393c.jpg]

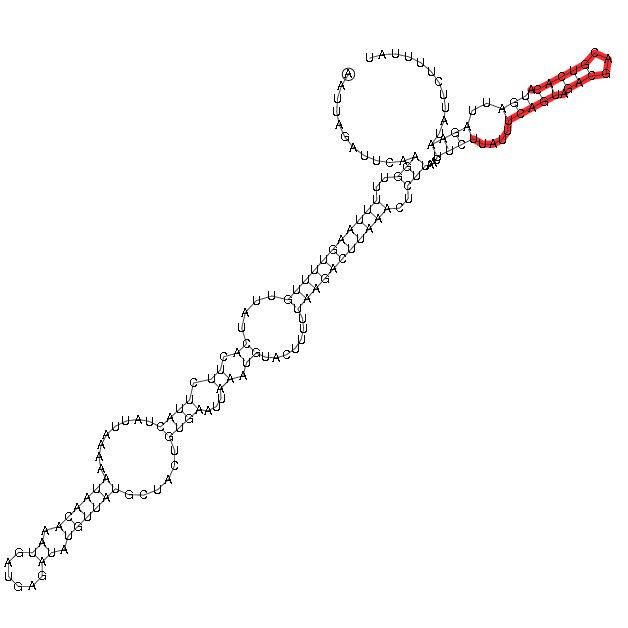

Supplement: Supplementary file 1 [file genes-13-01706-s001.zip › Figure S1. Known miRNAs Structure/csi-miR3947-5p_csi-MIR3947.jpg]

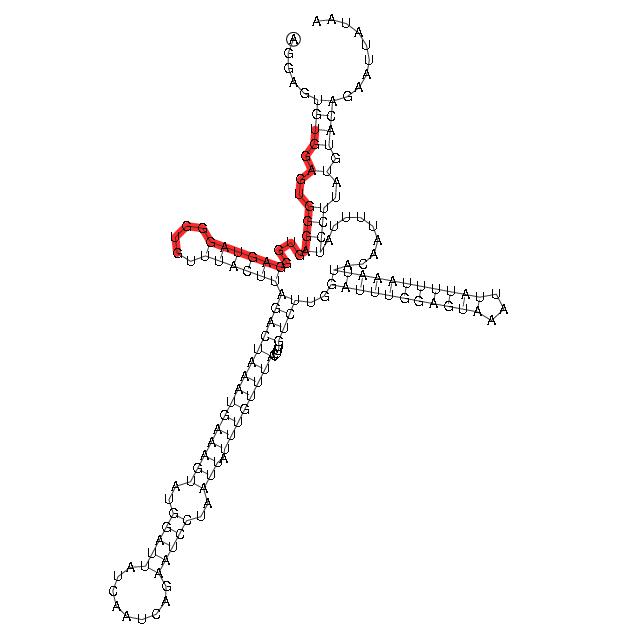

Supplement: Supplementary file 1 [file genes-13-01706-s001.zip › Figure S1. Known miRNAs Structure/csi-miR3948_csi-MIR3948.jpg]

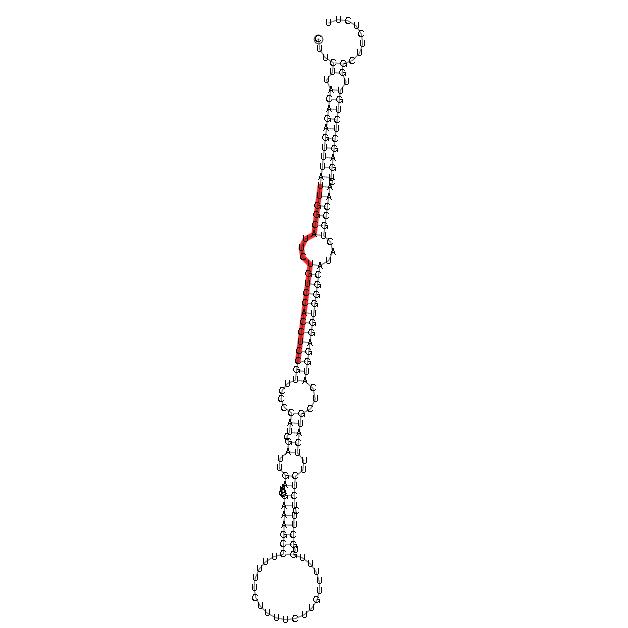

Supplement: Supplementary file 1 [file genes-13-01706-s001.zip › Figure S1. Known miRNAs Structure/csi-miR394a_csi-MIR394a.jpg]

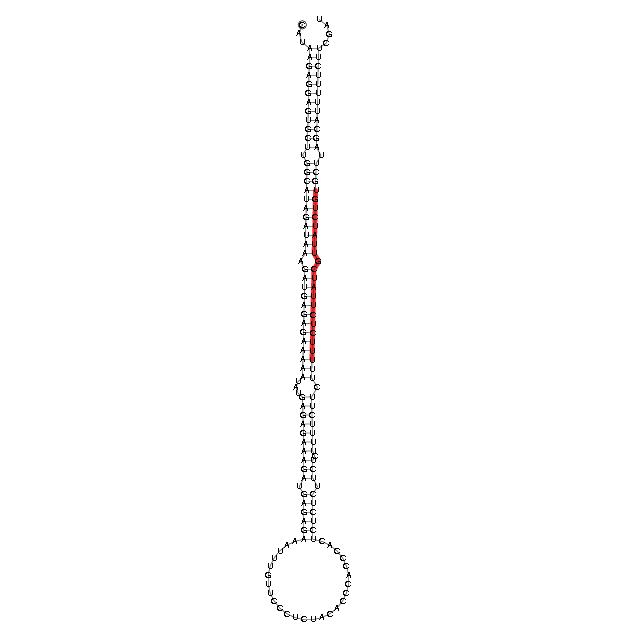

Supplement: Supplementary file 1 [file genes-13-01706-s001.zip › Figure S1. Known miRNAs Structure/csi-miR3951a-3p_csi-MIR3951a.jpg]

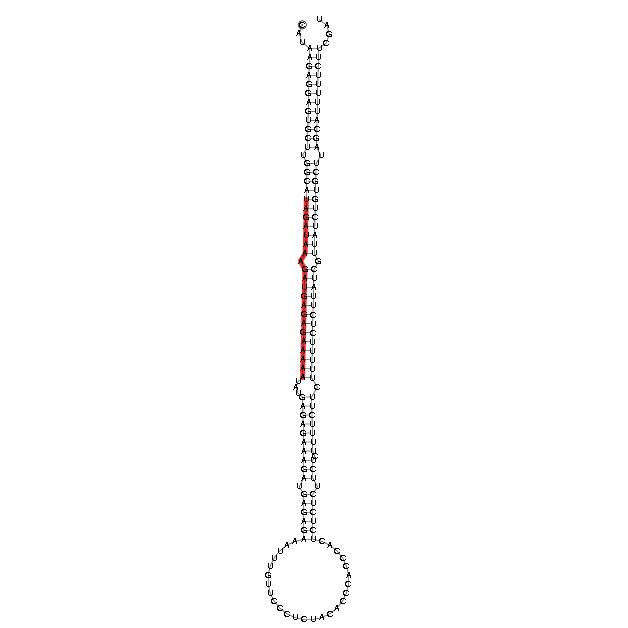

Supplement: Supplementary file 1 [file genes-13-01706-s001.zip › Figure S1. Known miRNAs Structure/csi-miR3951a-5p_csi-MIR3951a.jpg]

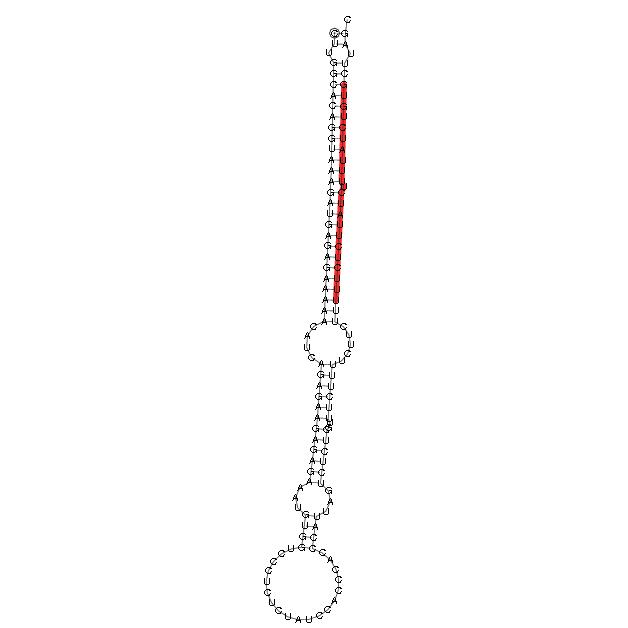

Supplement: Supplementary file 1 [file genes-13-01706-s001.zip › Figure S1. Known miRNAs Structure/csi-miR3951b-3p_csi-MIR3951b.jpg]

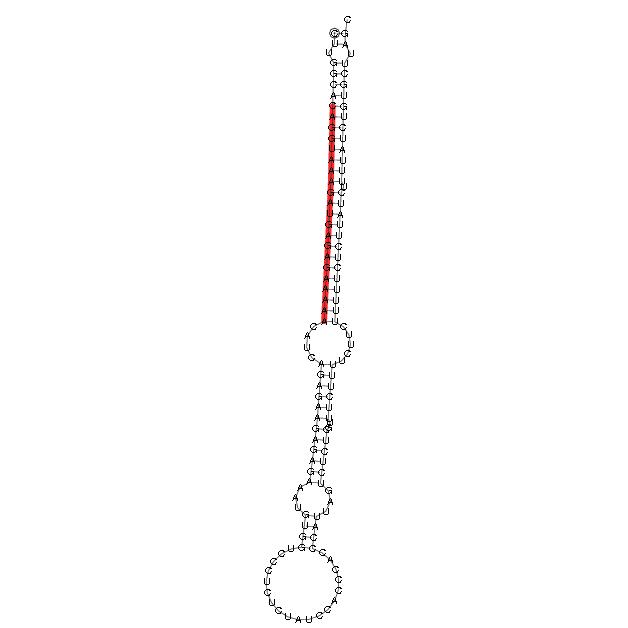

Supplement: Supplementary file 1 [file genes-13-01706-s001.zip › Figure S1. Known miRNAs Structure/csi-miR3951b-5p_csi-MIR3951b.jpg]

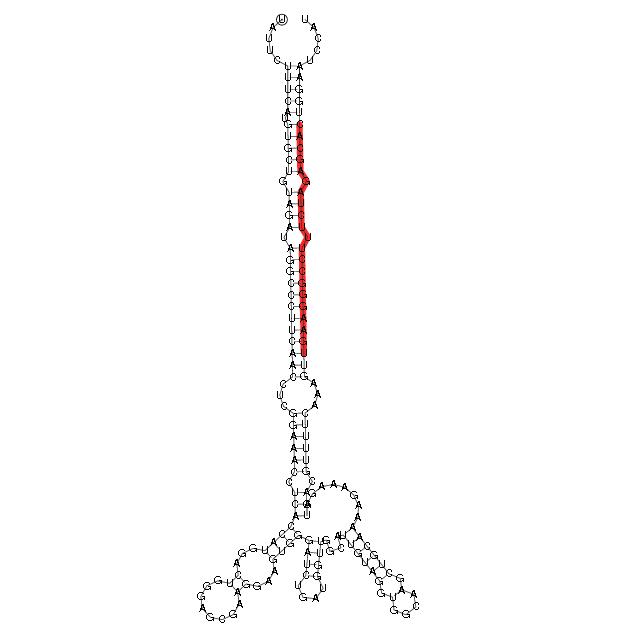

Supplement: Supplementary file 1 [file genes-13-01706-s001.zip › Figure S1. Known miRNAs Structure/csi-miR3952-3p_csi-MIR3952.jpg]

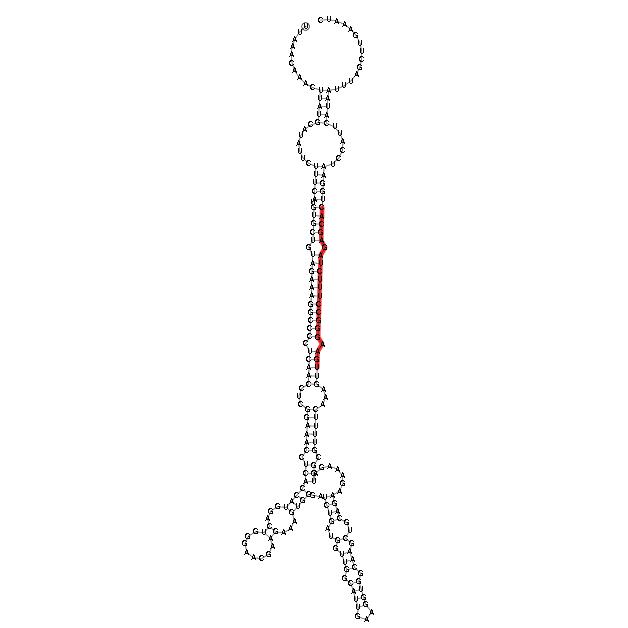

Supplement: Supplementary file 1 [file genes-13-01706-s001.zip › Figure S1. Known miRNAs Structure/csi-miR3952-3p_csi-MIR3952b.jpg]

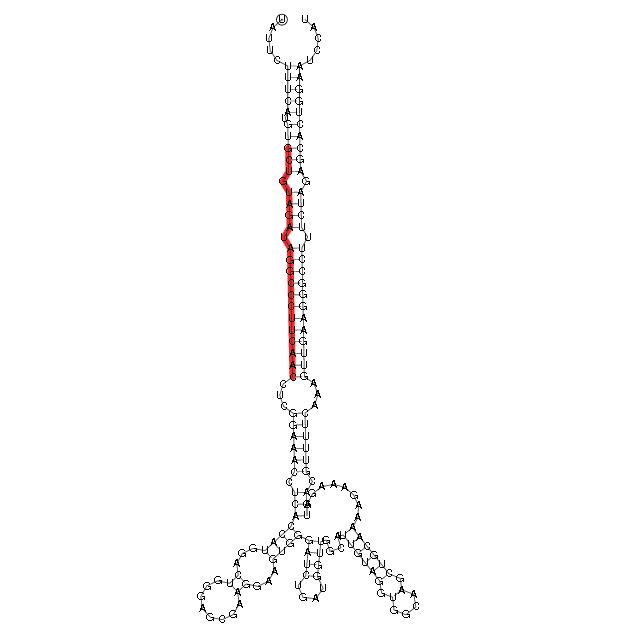

Supplement: Supplementary file 1 [file genes-13-01706-s001.zip › Figure S1. Known miRNAs Structure/csi-miR3952-5p_csi-MIR3952.jpg]

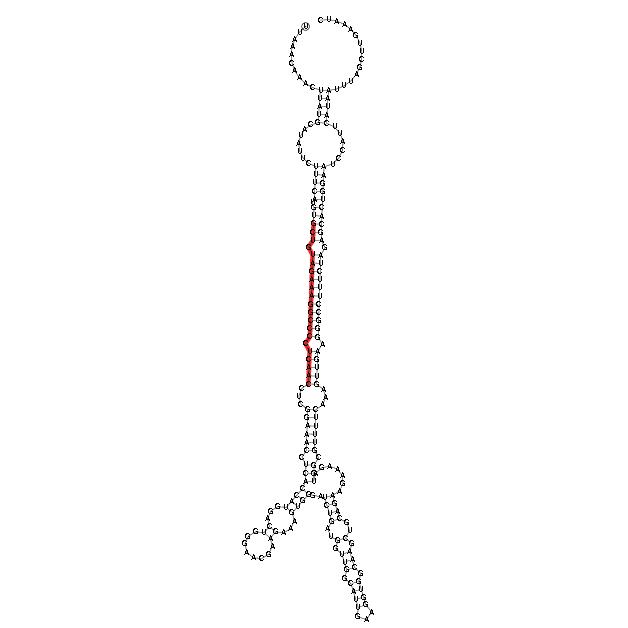

Supplement: Supplementary file 1 [file genes-13-01706-s001.zip › Figure S1. Known miRNAs Structure/csi-miR3952b-5p_csi-MIR3952b.jpg]

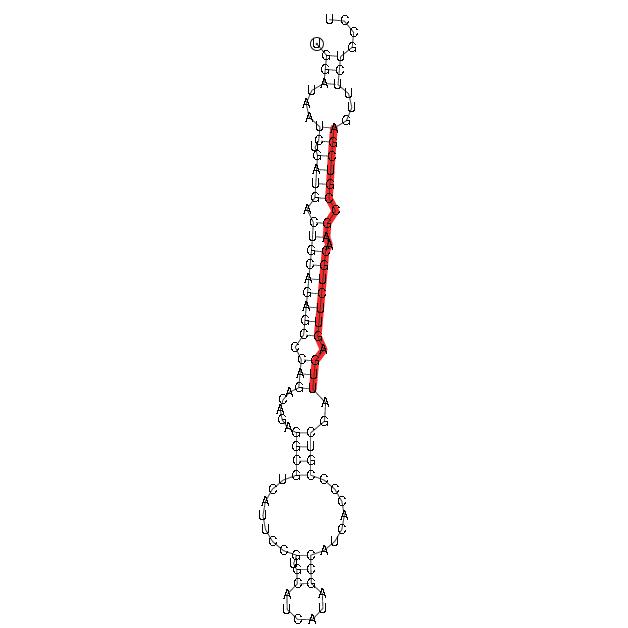

Supplement: Supplementary file 1 [file genes-13-01706-s001.zip › Figure S1. Known miRNAs Structure/csi-miR3953_csi-MIR3953.jpg]

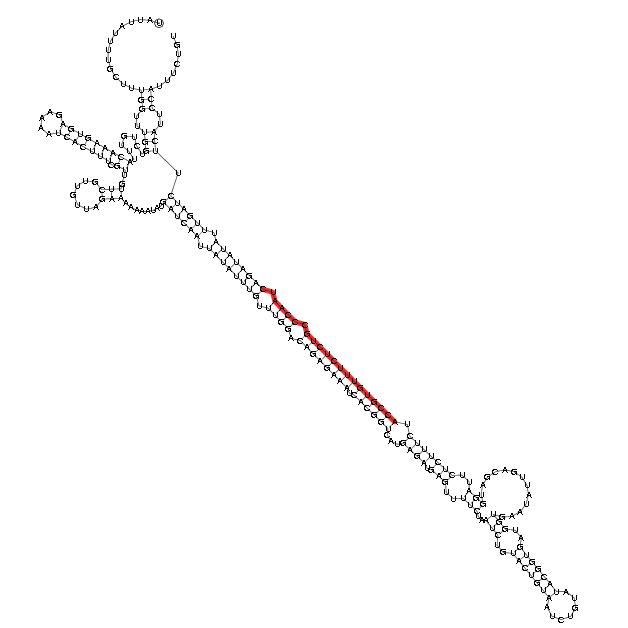

Supplement: Supplementary file 1 [file genes-13-01706-s001.zip › Figure S1. Known miRNAs Structure/csi-miR3954b-3p_csi-MIR3954b.jpg]
