# Supplementary material for: Comparative Transcriptome and sRNAome Analyses Reveal the Regulatory Mechanisms of Fruit Ripening in a Spontaneous Early-Ripening Navel Orange Mutant and Its Wild Type
Source: Genes (Basel). 2022 Sep 22;13(10):1706. doi: 10.3390/genes13101706 (PMC9601947; doi:10.3390/genes13101706)
Supplement: Supplementary file 1 [file genes-13-01706-s001.zip › Table S10 The primer sequences of target gene for qRT-PCR.pdf]

Table S10 The primer sequences of target gene for qRT-PCR

Gene name	Forward Primer 5′to3′	Reverse Primer 5′to3′
orange1.1t02597	ATTCACATTCCTGCAAGGCG	CTAGGCGGCATCATTTGGCT
Cs7g10990	ATTGGGGAATTGTGGCAGCA	GGCTGGCATTACTAGGCTGA
Cs1g03640	ACGAGTTGATGACTGGGAGA	CAATGGCTGCACGGTTTTCA
orange1.1t02265	GCTGGGACTGGTTTCTCTGG	TGACGGATTTGGTATGGGCA
Cs7g11770	GGCAACAAGAAACAACATAGGCT	CTAATCAGGGGCCTAGCCATT
Cs7g10830	ATGGCTGAGGTTCTTGACCA	GGCATGACAGCGTGAGGTAT
Cs8g16440	TGACGGCCAATTCACTATGC	GTGAACCGCTGCAAACTGG
Cs7g25670	CGTGACTAGCAGCCCCTTTT	GCTGAAAGCCCAGTGGAAAC
Cs6g11800	TATCCCCCTTCTCACCACCA	GAAACCAGCCGGAGACAGAC
Cs3g18940	ATTCAACAGTGGGGTGCCTG	CTCAGTGGACACACGTCTCA
Cs6g11860	GCCACTCTTCCCTTGCTCTG	GTATCGGCTTGGGGATTGGT
Cs6g07800	CAGGGAGGCTTTACATTGCC	GAGAGCAGGACATTGGTGGT
Cs6g07410	AACCTACACGCTCGAAGTGG	GGCTTTCAGCAAAACGTCCG
Cs7g23490	CGCAGCCAAAGACACTTTCA	TGGGGTGATGAGCACAACTTT
Cs6g07400	AGACCTCTATCCCTGCTCGG	TCAGTGTACCCAGCGTCAAC
Cs8g18800	TTCCGGGGCCAACATACAAT	GCTTCAACAACAGTGAGCGT
Cs7g31620	GTTTCAGATGCGCACACCAT	AGGGCTTAGTGTAGGATGAGTC
Cs7g27790	GGGCATGGCAAATGCACAAT	GCGGCAGATGTGTTTTGTGT
Cs6g04120	GGCAATTTGAAGCCCCTCTTG	TGATCGACTGGGTGTAACGG
orange1.1t04055	AGGCCTAAGCAGCAAGAAGC	GCTGGTGACTGAAGATGGGT
orange1.1t05093	CGTGGGGTTCAGATTCAAGC	TGAATCTCAGAATGCCGGGG
orange1.1t05587	ACTCTCTCACTCTTACTCTTACGTT	TAGCTCCCAAGGCTCAAAGC
Cs7g22510	TGGACAGTCTCACAGGCATTG	GTTGTTGTTGTCCTGACCGTTT
Cs7g22540	AGTCTCGTGGGGTTCAGATTCT	TGTTCCTCTATTTACCCGTCTACTC
Cs2g08080	ATCCCAGTAACAGCCACCCA	GGGTGGTGCAGTACCGATT
Cs3g06390	GGGTAGCCACACCCTTTCAA	GTCAAGTGTCCCAGTCGGTG
Cs8g16870	CCCGCTACCAGCTTCATTGAT	AGTAACCTGAACCTGCGGC
Cs2g15820	CAGAAACGAGAGGGACTGGC	GCCTGTTACTGTGCTGGTGA
orange1.1t03355	AAGATGGCGGAGTTGGTGTT	ACTAATGCCTCTGGGCACAC
Cs1g19320	CTGCTGCTGTGGAGACGAAA	GCTGCTTGAAGGTGGAGAATG
ACTB	CCAATTCTCTCTTGAACCTGTCCTT	TGACTGATGAGAACTGCCAGAAG
