# Supplementary material for: Comparative Transcriptome and sRNAome Analyses Reveal the Regulatory Mechanisms of Fruit Ripening in a Spontaneous Early-Ripening Navel Orange Mutant and Its Wild Type
Source: Genes (Basel). 2022 Sep 22;13(10):1706. doi: 10.3390/genes13101706 (PMC9601947; doi:10.3390/genes13101706)
Supplement: Supplementary file 1 [file genes-13-01706-s001.zip › Table S4 Family analysis of miRNAs.pdf]

# Table S4 Family analysis of miRNAs

| miRNA Family | Detected known and novel miRNAs | | | | |
|---|---|---|---|---|---|
| miR159 | novel_106 | novel_68 | novel_118 | csi-miR159 | |
| miR166 | csi-miR166b | csi-miR166c | csi-miR166d | csi-miR166e | csi-miR166a |
| miR167_1 | csi-miR167a | csi-miR167b | csi-miR167c | | |
| miR162_1 | csi-miR162 | | | | |
| miR171_1 | csi-miR171a | | | | |
| miR172 | csi-miR172a | csi-miR172b | csi-miR172c | | |
| miR171_2 | csi-miR171b | csi-miR479 | | | |
| miR396 | csi-miR396a | csi-miR396b | csi-miR396c | | |
| miR397 | csi-miR397 | | | | |
| miR399 | csi-miR399a | csi-miR399b | csi-miR399c | csi-miR399d | csi-miR399e |
| miR408 | csi-miR408 | | | | |
| miR477 | csi-miR477a | csi-miR477b | csi-miR477c | csi-miR473 | |
| miR482 | csi-miR482a | csi-miR482b | csi-miR482c | csi-miR472 | |
| miR530 | csi-miR530a | csi-miR530b | | | |
| miR827_3 | csi-miR827 | | | | |
