# Supplementary material for: Comparative Transcriptome and sRNAome Analyses Reveal the Regulatory Mechanisms of Fruit Ripening in a Spontaneous Early-Ripening Navel Orange Mutant and Its Wild Type
Source: Genes (Basel). 2022 Sep 22;13(10):1706. doi: 10.3390/genes13101706 (PMC9601947; doi:10.3390/genes13101706)
Supplement: Supplementary file 1 [file genes-13-01706-s001.zip › Table S8 The primer sequences of Key gene for qRT-PCR.pdf]

Table S8 The primer sequences of Key gene for qRT-PCR

Gene name	Forward Primer 5′to3′	Reverse Primer 5′to3′
Cs5g33420	GGCGGGTGTTTACGAGGAAT	CACGGCAAAAGTCGCAGAAT
Cs2g17070	ATAAAAGGAACGGTGGCCGA	AGCCACACCCTCTGAAATGG
orange1.1t02530	GTGCACTGCTGTACCGCTT	CCATCAGCGTTGGTACAGGA
orange1.1t02529	GACAGGCTTCGGGGGTTAAA	CCGATCCATCAGCGTTGGTA
Cs6g14340	TGGATCAAGCCCGACAACAA	TCCAGTGTCCGTCATTGCTC
Cs5g19060	TGGGCAGGGTAGGGTTCATA	TTCTCCCCTGGCCATCTGTA
Cs2g10900	TAGCTGCAACAGCCAGGAAA	ACACAATCACACCCTTGCCA
orange1.1t02108	GTAAGCCCTGCTGCTCTTGA	TCACCGGAACGCTCATTAGG
Cs5g14370	TTGGACAAGAATGCCACCGA	GTCGGCAGGAGTCATACAGG
Cs9g11260	AGCATCAAGACCCAGTTCGG	GTCACCTCACCAGTTCCCAG
Cs2g03270	TGCATGACACCTGCTGACTC	GCTGTTGCGATTCACCATCC
orange1.1t00416	ACGGGTGGAAGGCATTTGAT	TTGCCACGGCCTGTCTAAAT
Cs2g02500	CCATACTGATGCTGGTGGCA	ACGCGATGTAGCACACTCTT
Cs5g29870	GACTCAGAGGAAACGCTGCT	ATGTCTTGCCCGATGATCCC
Cs9g10650	GCATGGGATTAGGGTTTGGC	AGCATCCCTCCTCGGTGTTA
orange1.1t03771	AGGGACCCAACGAGCGA	TTGACTTCACAGTAATAACAGCCG
Cs1g23060	CTCCGGTAACTTCGGTGTGG	CCCACCGGATGCATATTCCA
Cs8g02900	ACATCATCCCTGCTCTTGCC	ATCGTGTACGTCCCTACCCA
Cs5g17900	AGGGGCAGATAGGGTGGATT	TCGCAGCAACTTCTTCTCCC
orange1.1t00550	AGAGGCGTGAGAAGCTGAAC	CACGGATGTGGTGGTGCTAT
Cs7g16980	AGGAGGACAAACATCACGG	TGGCAACCCAATCTCAAGC
Cs6g10710	CTGCCGTCCGTCATCTT	TCTCCATCGCCTCTTCC
Cs4g06710	TGTGAATAGCCTCGGTCTC	TATGTGCTGTTCTTTGGGT
Cs5g09220	GCAATCCGCCACTTTCC	TATCCTGCCGACCCATCT
Cs7g05690	TAGGCTTGCGTGGATTTG	ATAACGGAGATGTGGATGGG
orange1.1t03668	TTCCCGCTCCAATCTCC	CCCAGTGAACTTGGCAGAT
Cs5g26080	TAATACTTGTGGACCAGAGCCT	TGAACCCTTCTGCCAACCTC
Cs3g11040	TCCGCTCCATTTCCATC	AGGTCCCTCACCGCACA
Cs4g14850	TTGCGTAGCCGTCAAAGAA	CCACAACCAATCACCACCA
Cs9g19270	TAATCAACGCAGTTCCAGC	CAAACACCGTAATGCCAAGT
Cs5g34270	CGAGCGAGGTGATAAGATTG	AGCCAGATGGTTGGAGTT
Cs5g07700	GAGGGAAATCATCAACCACA	AGTTCACCACCAGCAGCAT
Cs8g19140	CGGGAGGGAGGGTAATC	CACAGACCGTCCGTTGC
Cs4g14980	GGCAAAGTCATACAGTGGAACG	CAAACCATCGCTGGCTAA
Cs5g03060	ATCACGGCTTGCCAGAG	GGTTGCTCCACCACTCA
Cs5g20590	GGGGTGTTTGTTCAGGT	TTGCCATCAAGTCTATTCC
Cs9g08850	GAAACCCAGAAAGTGAGCG	CACAGGACAATGAAGGCAAT
orange1.1t02185	TTGTTCGGCACTAAACTCGG	CCTTTGTGGTGGCGGTATT
orange1.1t00464	TCCTACGCCTTTAGAAACAGAG	CAGTTCCAGCATTCAGACCC
Cs1g17220	CGGGAAAGTGTTCGTCT	GCTATTGGCACCGTCTG

Cs4g06520	CGTCAACAACAACCACCAC	GCCTTCGGCTTCACTTC
Cs7g02820	GATTTCTTCGGCTTGGA	CGTAATCGGCTCTTCTTT
Cs4g07130	ATGGCGGACAAGTGATT	CATAGGAGGATAAGCGGTA
Cs1g17210	CGCCTCCAATGTGATGAA	ACTTGGTATGGTGCTCTTGC
orange1.1t01021	AATCAAGTGGTGGTGCTG	CTTCCATTTGCGGGTTT
Cs5g01450	AGGAAGAAAGCCAACAAGCG	ATGTAGGCAACAGCATCAGC
orange1.1t03769	AGAAATGGGCGAAGAAG	TGTAGTCGTGGACCGTAGAT
orange1.1t04376	GCCAAATGGTCCTGTCA	TTCAATCCGTCTGGTGTT
Cs1g17380	TACCCGTATGGTGGTTATG	ATGTATTTGGTCGCTTGTAG
Cs3g24230	AAACGGCGGCAAAGTGA	ATTGTAACGCTACGGGTGG
orange1.1t03727	GTAGATTGGTCACCTCCTCGTC	TAGGCTTCCGCCGTATTGA
Cs4g15220	CAGAAGATGGTGCTGATTTGG	TCAGGAAAGGATAGTCCGTGTA
Cs5g17880	GGGCAGATAGGGTTGGGATA	ATGGGCAGCAAACTGTGAGG
ACTB	CCAATTCTCTCTTGAACCTGTCCTT	TGACTGATGAGAACTGCCAGAAG
