# Supplementary material for: Comparative Transcriptome and sRNAome Analyses Reveal the Regulatory Mechanisms of Fruit Ripening in a Spontaneous Early-Ripening Navel Orange Mutant and Its Wild Type
Source: Genes (Basel). 2022 Sep 22;13(10):1706. doi: 10.3390/genes13101706 (PMC9601947; doi:10.3390/genes13101706)
Supplement: Supplementary file 1 [file genes-13-01706-s001.zip › Table S9 The primer sequences of miRNA for qRT-PCR.pdf]

Table S9 qRT-PCR primers for miRNA

Primers for qRT-PCR	Sequence（5′-3′）
csi-miR156f-5p -F	CGCGGCATGAGAGAGAGAAG
csi-miR160c-5p -F	CGGCGTTCGTATGTCCCTCG
csi-miR397-5p -F	GCGGTAGTTGCGACGTGA
csi-miR172a-3p -F	GCCGGCACGTCGTAGTAGTT
csi-miR3954 -F	GCGGCACTGGCACTAAAGAG
miRN106 -F	CGGCTCCTCGAGGGAAGTC
miRN127 -F	GCGGCATATAACTTCAAATTCG
miRN54 -F	GCTCCCATTTGCCTGTCTGT
common -R	ACTGCAGGGTCCGAGGTATT
U6-F	CTCGCTTCGGCAGCACA
U6-R	AACGCTTCACGAATTTGCGT
